# A function of Spalt proteins in heterochromatin organization and maintenance of genomic DNA integrity

**DOI:** 10.1242/dev.204258

**Published:** 2025-05-16

**Authors:** Cristina M. Ostalé, Natalia Azpiazu, Ana Peropadre, Mercedes Martín, Mireya Ruiz-Losada, Ana López-Varea, Rebecca R. Viales, Charles Girardot, Eileen E. M. Furlong, Jose F. de Celis

**Affiliations:** ^1^ Centro de Biología Molecular ‘Severo Ochoa’, Department of Tissue and Organ Homeostasis, CSIC and Universidad Autónoma de Madrid, Madrid 28049, Spain; ^2^ Department of Biology, Universidad Autónoma de Madrid, Madrid 28049, Spain; ^3^ European Molecular Biology Laboratory, Genome Biology Department, Heidelberg 69117, Germany

**Keywords:** Spalt proteins, Gene expression, Heterochromatin, Nuclear lamina, Nucleolus, *Drosophila*

## Abstract

The conserved Spalt proteins regulate gene expression and cell fate choices during multicellular development, generally acting as transcriptional repressors in different gene regulatory networks. In addition to their roles as DNA sequence-specific transcription factors, Spalt proteins show a consistent localization to heterochromatic regions. Vertebrate Spalt-like proteins can act through the nucleosome remodeling and deacetylase complex to promote closing of open chromatin domains, but their activities also rely on interactions with DNA methyltransferases or with the lysine-specific histone demethylase LSD1, suggesting that they participate in multiple regulatory mechanisms. Here, we describe several consequences of loss of Spalt function in *Drosophila* cells, including changes in chromatin accessibility, generation of DNA damage, alterations in the localization of chromosomes within the nucleus in the salivary glands and misexpression of transposable elements. We suggest that these effects are related to roles of Spalt proteins in the regulation of heterochromatin formation and chromatin organization. We propose that *Drosophila* Spalt proteins have two complementary functions, acting as sequence-specific transcriptional repressors on specific target genes and regulating more global gene silencing through the generation or maintenance of heterochromatic domains.

## Teaser

**Summary:** The *Drosophila* Spalt proteins regulate gene silencing through the maintenance of heterochromatic domains. Loss of Spalt alters chromatin accessibility, causes misexpression of transposable elements and generates DNA damage and alterations in the localization of chromosomes within the nucleus.

## INTRODUCTION

The Spalt proteins (Sal) regulate gene expression (de Celis and Barrio, 2009; Sweetman and Münsterberg, 2006), and present between seven and ten C2H2 zinc-finger domains, a poli-Q stretch and several sites of sumoylation (Sánchez et al., 2010, 2011). They play multiple roles during the development of vertebrates and invertebrates, including vulva formation and cell fate specification in *Caenorhabditis elegans* (Grant et al., 2000; Jarriault et al., 2008; Shen et al., 2017; Toker et al., 2003), and organ development in vertebrates (Álvarez et al., 2021; Sweetman and Münsterberg, 2006). The *Drosophila* sal genes, *spalt major* (*salm*) and *spalt-related* (*salr*), form part of a gene complex (Barrio et al., 1999) and participate in the acquisition of segmental identity, the development of the Johnston organ and dorsal trunk trachea, the specification of photoreceptor cells in the eye, and the growth and patterning of the imaginal wing disc (de Celis et al., 1996; Domingos et al., 2004; Eberl and Boekhoff-Falk, 2007; Kühnlein and Schuh, 1996; Kühnlein et al., 1994).

The diversity of roles played by Sal proteins is in part due to their participation in different transcriptional gene regulatory networks operating in different tissues and developmental times (Martín et al., 2016). Within these networks, Sal function impacts the transcriptional profile of cells, altering the expression of a multitude of genes in mutant conditions. This has been shown in murine kidneys (Basta et al., 2017), odontoblasts (Lin et al., 2021), embryonic stem cells (Miller et al., 2016; El Pantier et al., 2021), *Drosophila* wing discs (Organista et al., 2015), and during tumoral transformation (Yang et al., 2017). Human SALL1 and SALL4 are linked to the genetic syndromes Townes-Brocks (SALL1; Kohlhase, 2000; Kohlhase et al., 1999) and Okihiro (SALL4; Borozdin et al., 2004), which are characterized by malformations in the limbs and different internal organs. Additionally, human Sall genes are frequently misexpressed in cancers (Álvarez et al., 2021), which might be related to their roles in the maintenance of pluripotency (Sakaki-Yumoto et al., 2006; Tsubooka et al., 2009; Yang et al., 2008).

There is still not a unifying framework to understand the function of Sal proteins as transcriptional regulators. On the one hand, they directly regulate specific targets by binding to regulatory sequences. For example, *Drosophila* Salm/Salr repress *knirps* (*kni*) acting through enhancers located 11 Kb from the *kni* transcription start site (TSS; Ostalé et al., 2024). Likewise, the *C. elegans* Sal-like protein Sem-4 represses *egl-5* and *mec-3* during neuronal differentiation through the binding of Sem-4 to the promotors of these genes (Toker et al., 2003). Similarly, mouse SALL2 regulates cell proliferation in mouse embryonic fibroblasts, repressing *Ccnd1* and *Ccne1* (Hermosilla et al., 2018). Further examples of sequence-specific transcription factor activity have been reported for SALL4 on *HOXA9* expression during human hematopoiesis (Gao et al., 2013), on glycolytic enzymes in murine limbs (Kawakami et al., 2023), and for *pou5f3* repression by Sall1/Sall4 during *Xenopus* neural patterning (Exner et al., 2017). These results indicate that SAL proteins can act as transcriptional repressors, binding DNA in a sequence-specific manner.

On the other hand, Salm in *Drosophila* (Ostalé et al., 2024), and SALL1 and SALL4 in vertebrates (Netzer et al., 2001, 2006; Sakaki-Yumoto et al., 2006), preferentially locate to heterochromatic regions, and SALL1 interacts with several heterochromatic proteins (Netzer et al., 2001), suggesting an activity of Sal proteins in the organization of heterochromatin and the regulation of gene expression by epigenetic modifications (Netzer et al., 2001, 2006; Sakaki-Yumoto et al., 2006). In addition, SALL1 and SALL4 interact with the nucleosome remodeling and deacetylase (NuRD) complex, suggesting that NuRD is, in several cell contexts, a central facilitator of the repression mediated by these proteins (Kiefer et al., 2002; Lauberth and Rauchman, 2006; Lauberth et al., 2007; Wang et al., 2023; Miller et al., 2016). Finally, Sall4 function has also been linked to the recruitment of the lysine-specific histone demethylase LSD1 (also known as Kdm1a; Liu et al., 2013). The variety of Sal interaction partners suggests that Sal proteins recruit epigenetic complexes to either cell-type specific target gene promotors or chromatin domains to regulate gene expression or chromatin structure.

Here, we analyzed different *Drosophila* tissues expressing the *salm* and *salr* genes to investigate the possible contributions of Sal proteins to chromatin organization. We find that loss of *salm* and *salr* results in changes in chromatin accessibility in imaginal cells, but that these changes bear little relation to the changes in gene expression detected in a similar genetic background. In addition, these changes mostly affect pericentromeric heterochromatic regions, which are the regions preferentially bound by endogenous Salm proteins in imaginal cells (Ostalé et al., 2024). We also found an unexpected relationship between Sal function and the maintenance of genome integrity and transposable elements silencing. We suggest that these effects are due to defects in heterochromatin formation and/or maintenance in sal mutant cells. Altogether, our results suggest that diploid imaginal sal mutant cells display altered heterochromatin assembly and/or organization, suffer mitotic errors and DNA damage, trigger DNA-damage responses and fail to progress to mitosis, whereas sal mutant polyploid cells have defects in nuclear envelope morphology and nucleolar size accompanied by changes in the nuclear disposition of chromatin. We suggest that these functions of Sal proteins underly the majority of changes in gene expression observed in sal mutant cells, and define a global role for these proteins in heterochromatin maintenance that complements their activities as sequence-specific transcription factors.

## RESULTS

### Loss of Spalt function has a profound impact on chromatin accessibility

**Fig. 1.**
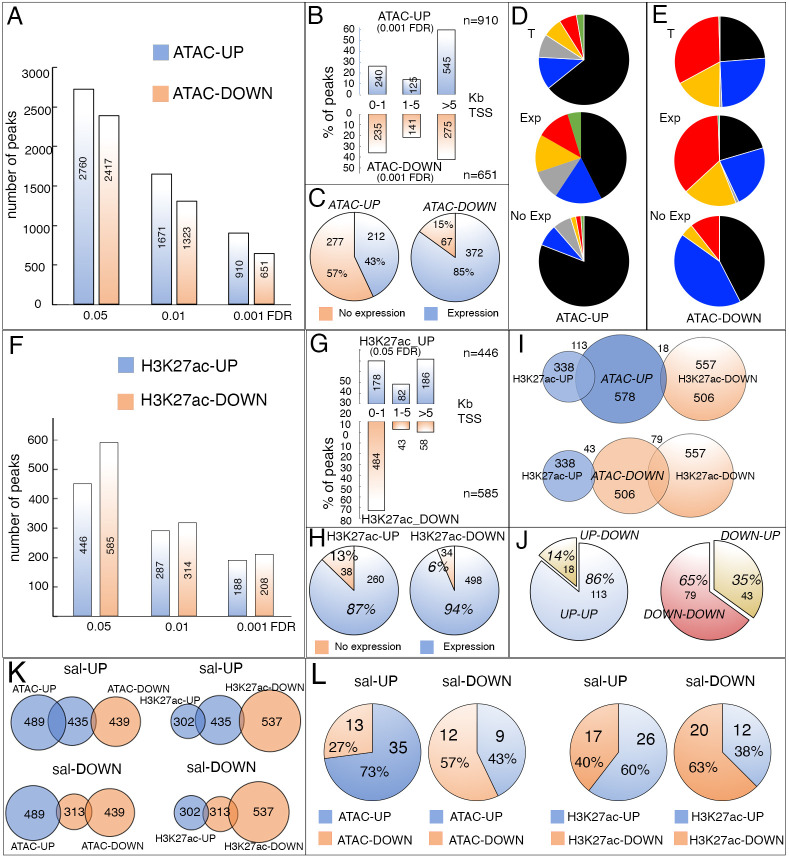
**Changes in chromatin accessibility in *salm/salr* knockdown imaginal discs.** (A) Number of genomic regions (peaks) showing enrichment (ATAC-UP; blue) or depletion (ATAC-DOWN; red) comparing ATAC-seq data from *salm/salr* RNAi knockdown (*sal^EPv^-Gal4/UAS-salm-RNAi; UAS-salr-RNAi/+*) and control (*sal^EPv^-Gal4 UAS-GFP/+*) wing discs. The DNA regions were identified at 0.05, 0.01 and 0.001 false discovery rates (FDR). (B) Percentage of peaks located 0-1, 1-5 and more than 5 Kb from the nearest transcription start site (TSS). ATAC-UP and ATAC-DOWN peaks are shown in blue and red, respectively. (C) Percentage of genes expressed (Expression; blue) or not expressed (No expression; red) in the wing disc in the ATAC-UP (left) and ATAC-DOWN (right) datasets. The percentage and absolute number of genes are indicated by numbers. (D,E) Percentage of sequences belonging to the ATAC-UP (D) and ATAC-DOWN (E) classes included in black, blue, green, yellow and red chromatin domain states (Filion et al., 2011). The black, blue and green states represent different types of heterochromatin, and the red and yellow states correspond to euchromatin. The gray sector represents sequences not associated to any of these stages. The data refer to the total number of genes analyzed (T; up), to genes expressed in wild-type imaginal discs (Exp; middle) and to genes not expressed in the wing disc (No Exp; bottom). (F) Number of genomic regions (peaks) showing enrichment (H3K27ac-UP; blue) or depletion (H3K27ac-DOWN; red) comparing the H3K27ac ChIP-seq data of *sal^EPv^-Gal4/UAS-salm-RNAi; UAS-salr-RNAi/+* and *sal^EPv^-Gal4 UAS-GFP/+* wing discs. The DNA regions were identified at 0.05, 0.01 and 0.001 FDR. (G) Percentage of H3K27ac peaks located 0-1, 1-5 and more than 5 Kb from the nearest TSS. The H3K27ac-UP and H3K27ac-DOWN peaks are shown in blue and red, respectively. (H) Percentage of genes expressed in the wild-type wing disc (Expression; blue) or not expressed in the wild-type wing disc (No expression; red) in the H3K27ac-UP (left) and H3K27ac-DOWN (right) datasets. (I) Comparison between genomic regions showing ATAC-seq enrichment or depletion (ATAC-UP: FDR 0.05, blue; ATAC-DOWN: FDR 0.05, red) with those enriched or depleted in the H3K27ac ChIP-seq dataset (H3K27ac-UP and H3K27ac-DOWN, respectively). (J) Frequencies of sequences included in both the ATAC and H3K27ac datasets: ATAC-UP and H3K27ac-UP (UP-UP) and ATAC-UP and H3K27ac-DOWN (UP-DOWN) are shown in the left graphic, ATAC-DOWN and H3K27ac-UP (DOWN-UP) and ATAC-DOWN and H3K27ac-DOWN (DOWN-DOWN) are shown in the right graphic. (K) Number of genes for which expression is augmented (Sal-UP) or reduced (Sal-DOWN) in *salm/salr* mutant discs included in the ATAC-UP (blue), ATAC-DOWN (red), H3K27ac-UP (blue) and H3K27ac-DOWN (red) datasets. (L) Frequency of genes included in both the *salm/salr* RNA-seq expression datasets (sal-UP and sal-DOWN) and the ATAC-UP (blue), ATAC-DOWN (red), H3K27ac-UP (blue) and H3K27ac-DOWN (red) datasets. The number of genes included in these calculations are indicated in each circle.

*Salm/salr* mutant wing discs display changes in the expression levels of a large set of genes (Organista et al., 2015). Here, we analyzed variations in chromatin accessibility in wild-type discs compared to *salm/salr* knockdown discs, aiming to find possible correlations between gene expression and chromatin accessibility. We carried out assay for transposase-accessible chromatin with sequencing (ATAC-seq) experiments in control discs (*sal^EPv^-Gal4 UAS-GFP/+*) and in *salm/salr* knockdown discs (*sal^EPv^-Gal4 UAS-GFP/UAS-salm-RNAi; UAS-salr-RNAi/+*) and detected considerable chromatin regions with accessibility changes (5177, 2994 and 1561 peaks at 0.05, 0.01 and 0.001 FDR, respectively; Fig. 1A and Table S1). The number of regions that increased or reduced their accessibility in *salm/salr* knock-down conditions compared to wild-type discs showed a ratio of 1.14, 1.26 and 1.39 at FDRs 0.05, 0.01 and 0.001, respectively (Fig. 1A; ATAC-UP/ATAC-DOWN values; Table S1).

We found 53% of sequences with accessibility changes located more than 5 Kb from the nearest TSS, including those located within large introns or between genes (Fig. 1B). The fraction of distal sequences associated to changes in accessibility is maximal for those showing increased accessibility (ATAC-UP; Fig. 1B). To relate the ATAC-seq data with gene expression, we associated the sequences with accessibility changes to their nearest TSS (Table S1), resulting in 623 and 506 candidate genes nearest to sequences for which accessibility increases or decreases, respectively (‘ATAC-UP’ and ‘ATAC-DOWN’ genes; Table S1), in *salm/salr* knockout wing discs. We defined wild-type expression for these genes in wild-type wing discs based on published results (Flegel et al., 2016; López-Varea et al., 2021), and found an enrichment for genes normally expressed in the wing disc in the ATAC-DOWN set, and an enrichment for genes normally not expressed in the wing disc in the ATAC-UP set (Fig. 1C). We also found a prominence of ATAC-UP and ATAC-DOWN sequences related to the chromatin states defined by Filion et al. (2011). Thus, ATAC-UP sequences are more likely found in repressed chromatin domains (black, blue and green states; Fig. 1D), whereas ATAC-DOWN sequences are more represented in regions of active chromatin (red and yellow states; Fig. 1E). This tendency is particularly clear when we consider whether the associated gene is or is not expressed in the wing disc (Fig. 1D,E). In this manner, ATAC-UP sequences are more likely found in distal regions to the TSS that prominently correspond to genes not expressed in the wing disc and placed in regions of ‘repressed chromatin’. In contrast, ATAC-DOWN sequences are preferentially associated to genes normally expressed in the wing disc placed in regions of active chromatin.

Active DNA regulatory regions are enriched for certain histone posttranslational modifications such as H3 acetylation in lysine 27 (H3K27ac) (Bonn et al., 2012; Fu et al., 2018). Loss of Sal function also has an impact on the presence of the H3K27ac mark in imaginal wing disc cells. Chromatin immunoprecipitation followed by sequencing (ChIP-seq) experiments with anti-H3K27ac antibodies in control and *salm/salr* knockdown discs reveal chromatin regions showing increased (446 peaks, FDR=0.05) or decreased (585 peaks, FDR=0.05) H3K27 acetylation (Fig. 1F; Table S1). The most obvious enrichment of H3K27ac ChIP sequences occurs for sequences showing reduced acetylation located at less than 1 Kb distance to the nearest TSS (Fig. 1G). In general, genes associated by proximity to sequences showing either increases or decreases in H3K27 acetylation are expressed in the wing disc (Fig. 1H). The overlap between changes in accessibility and changes in H3K27ac is low (Fig. 1I). For example, only 131 of the ChIP-seq H3K27ac regions (14.6%) are in the ATAC-UP class (Fig. 1I). Interestingly, 86% of these regions correspond to increases in H3K27 acetylation (Fig. 1J). Similarly, only 122 regions with modifications in H3K27ac are in the ATAC-DOWN class (Fig. 1I). In this case, most regions of overlap (65%) correspond to those showing reduced H3K27 acetylation (Fig. 1J).

The genes associated with changes in H3K27ac and/or changes in ATAC peaks are not correlated with those that change their expression in *salm/salr* knockdown. To do this comparison, we used a set of 435 and 313 genes with increased or decreased expression, respectively, after 24- or 48-h periods of *salm/salr* knockdown (Table S1; Organista et al., 2015). We found that only 144 of these genes were also present in either the differential H3K27ac or ATAC associated genes (Fig. 1K,L). The best overlaps between the three datasets include genes upregulated in *salm/salr* knockdown discs acquiring higher accessibility or enrichment in H3K27ac labeling (Fig. 1L). In conclusion, we find that a large fraction of changes in ATAC-seq and H3K27Ac binding are not correlated with the changes in gene expression observed in *salm/salr* knockdown wing imaginal discs, suggesting that these changes could be caused by indirect mechanisms triggered by *salm/salr* loss.

**Fig. 2.**
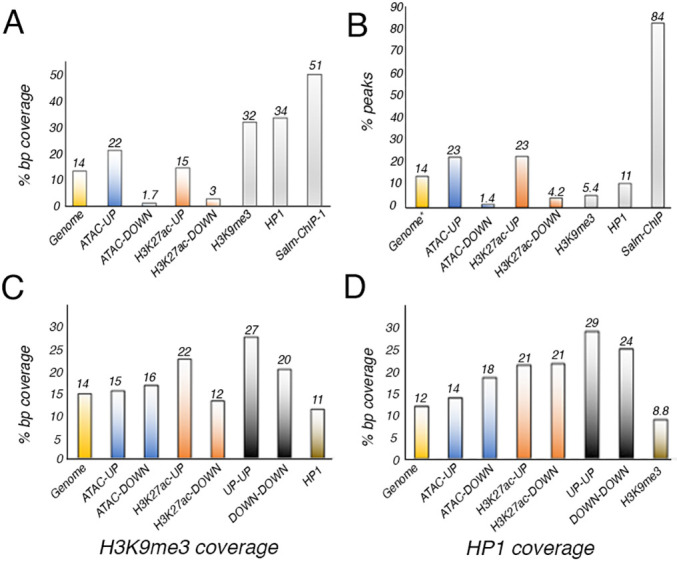
**Distribution of sequences identified in the ATAC and H3K27ac in relation to pericentromeric heterochromatin.** (A,B) Percentage of base pair coverage (% bp coverage; A) and overlapping peaks (% peaks; B) associated to pericentromeric heterochromatin in the ATAC (blue columns), H3K27ac (red columns), H3K9me3, HP1 and Salm ChIP experiments (gray columns). (C,D) Overlap between sequences identified in H3K9me3 ChIP-seq (C) and HP1 ChIP-seq (D) with all genomic sequences (yellow columns; Genome) or with sequences present in the ATAC (blue columns) or H3K27ac (red columns), and in both ATAC and H3K27ac datasets (UP-UP and DOWN-DOWN columns, gray). Sequences identified in H3K9me3 ChIP-seq present in the HP1 ChIP-seq experiments, as well as sequences identified in the HP1 ChIP-seq and present in the H3K9me3 ChIP-seq experiments are shown in the brown columns.

The Salm protein is preferentially bound to heterochromatic regions in Salm ChIP-seq experiments (Ostalé et al., 2024). Besides heterochromatin binding, we could only associate 113 genes with Salm binding to either promoters or distal regions (Ostalé et al., 2024; Table S1). Only 20% of these genes were associated to increased [*Tim23*, *CG17715*, *CG41520*, *UQCR-11*, *CG40228*, *Myo81F*, *Pzl*, *CR40454*, *CR43242*, *CR45864*, *DIP-lambda*, *AGO3*, *scro*, *l(3)80Fg*, *Cadps*, *CG17684*, *Hsp70Ba*, *side*, *bru3*, *flam*, *CG41562*, *CG14621*] or reduced (*tyn*, *Eip74EF*, *kirre* and *gpp*) chromatin accessibility (Table S1 and Fig. S2). The association between Salm binding and H3K27ac was even lower, and only 11% of genes associated to Salm binding display increased or reduced H3K27ac enrichment (Table S1). We also searched for the relationships between the sequences identified in ATAC-seq and H3K27ac ChIP-seq experiments with pericentromeric heterochromatic regions, which includes ∼14% of the *Drosophila* genome (He et al., 2012; Table S2). We only found a particular enrichment (*P*<0.05) in pericentromeric areas for DNA regions that gain accessibility in *salm/salr* knockdown discs (22.1% of total bp with increased accessibility, with a corrected *P*-value FDR 0.001; Fig. 2A,B). These results suggest a role for Sal proteins in pericentromeric chromatin organization, specifically in the maintenance of chromatin with low accessibility. Constitutive heterochromatin, present in telomeric and centromeric regions of the chromosomes, is characterized by epigenetic repression marks such as the trimethylation of H3K9 or the association of Su(var)205 (HP1a) (Elgin and Reuter, 2013; Swenson et al., 2016). More than 30% of the sequence coverage of H3K9me3 and HP1 ChIP-sequencing align to pericentromeric regions (*P*<0.001; Fig. 2A). We explored whether the regions that suffer chromatin activity and accessibility changes after *salm/salr* knockdown are usually bound by HP1 and/or are located in H3K9me3 modified landscapes. For all sets (ATAC-UP, ATAC-DOWN, H3K27ac-UP and H3K27ac-DOWN), the proportion of nucleotides sequenced in each set that is also sequenced after HP1 or H3K9me3 chromatin immunoprecipitation is higher than the 11.8% of bp bound by HP1 in the genome (Fig. 2D; Table S2). We observed a specific enrichment for those regions that increased both its accessibility and H3K27ac abundance after *salm/salr* silencing (*P*<0.001, UP-UP in Fig. 2C,D).

### Requirements of *salm/salr* in the maintenance of heterochromatic regions

**Fig. 3.**
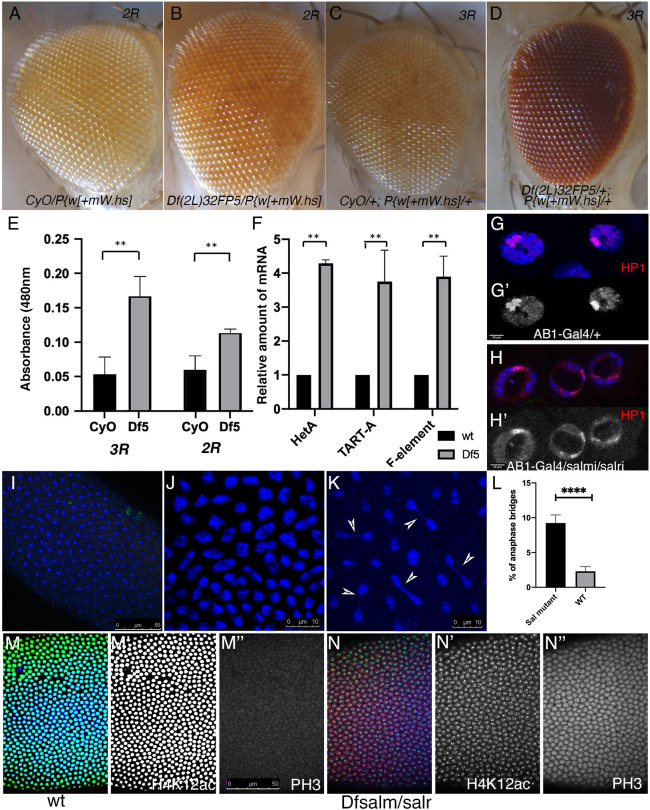
**Changes in gene expression in heterochromatic regions in *salm/salr* heterozygous flies and embryos.** (A,B) Eyes from 2-day old *w; CyO/P{w[+mW.hs]* (A) and *w; Df(2L)32FP5/P{w[+mW.hs]* (B) females. The insertion P{w[+mW.hs] is located in the telomere associated sequence (TAS) of the 2R chromosomic arm. (C,D) Eyes from 2-day old *w; CyO/+; P{w[+mW.hs]*/+ (C) and *w; Df(2L)32FP5/+; P{w[+mW.hs]/+* females (D). The insertion *P{w[+mW.hs]* is located in the TAS of the 3R chromosomic arm. (E) Eye pigment quantification (measured in absorbance at 480 nm) of *w; CyO/+; P{w[+mW.hs]*/+ and *w; Df(2L)32FP5/+; P{w[+mW.hs]/+* (two left columns) and *w; CyO/P{w[+mW.hs]*/CyO and *w; Df(2L)32FP5/P{w[+mW.hs]* (two right columns). (F) Expression (qPCR) of the retrotransposons HetA, TART-A and F-element in control embryos (wt; black columns) and in embryos obtained from *Df(2L)32FP5* heterozygous flies (Df5; gray columns). (G-H′) Distribution of HP1 (red) and DAPI (blue) in salivary gland nuclei from larvae of *AB1-Gal4 UAS-GFP/+* (G,G′) and *AB1-Gal UAS-GFP/UAS-salm-RNAi; UAS-salr-RNAi/+* (H,H′). The corresponding red channels are shown in G′ and H′. (I) Confocal images of mitotic chromosomes (DAPI, blue) of a syncytial blastoderm from heterozygous *Df(2L)32FP5* parents. (J,K) Higher magnification of independent fields showing defective mitotic figures such as asynchronic mitosis (J) and anaphase bridges (white arrowheads, K). (L) Frequency of anaphase bridges in blastoderms from heterozygous *Df(2L)32FP5* parents. (M-N″) Distribution of H4K12ac (green in M,N; white in M′,N′), Phospho-Histone3 (PH3, red in M,N; white in M″,N″) and DAPI (blue in M,N) in wild-type cycle 12 blastoderms (M-M″) and cycle 12 blastoderms from heterozygous *Df(2L)32FP5* flies (N-N″). The confocal settings were the same in all pictures shown in panels M-N″. **P<0.01, ****P<0.0001 (paired Student's *t*-test). Data are mean±s.d. Scale bars: 10 μm (G-H′, J,K); 50 μm (I,M-N″).

Salm binding to pericentromeric DNA as well as alterations in chromatin conformation affecting pericentromeric chromatin both suggest a role for Sal proteins in the generation and/or maintenance of heterochromatic regions that might lead to global effects in gene expression. Mutations in genes encoding proteins required for heterochromatin formation are often haplo-insufficient, and in heterozygous conditions increase the expression of genes placed into heterochromatic positions (Elgin and Reuter, 2013; Swenson et al., 2016). This effect has been studied mostly using the suppression of the white eye phenotype by transposable elements carrying *white^+^* insertions (Schotta et al., 2003). We compared the expression of *white* insertions present in the subtelomeric regions of the 2R and 3R chromosomic arms in *white* females heterozygous for a deficiency for the *salm* and *salr* genes [*Df(2L)32FP-5*; Barrio et al., 1999] and in their corresponding *CyO* siblings. For both subtelomeric insertions, we found a consistent increase in eye pigmentation (expression of the *white* gene present in *P{hsp26-pt-T}*) in *Df(2L)32FP-*5 heterozygous flies (Fig. 3A-E).

*Drosophila* telomers are maintained by retrotransposons, transcription of which is regulated by small RNAs and heterochromatin from adjacent telomeric associated sequences (TAS). Consequently, changes in heterochromatin organization in TAS are correlated with alterations in the expression of HeT-A and TART elements (Capkova Frydrychova et al., 2008). We found an average 4-fold increase in the expression of HeT-A and TART retrotransposon (Casacuberta and Pardue, 2005; Riddle et al., 2008) and F-element (localized in the heterochromatin of the fourth chromosome) in 0-2 h-old embryos in the progeny of *Df(2L)32FP-5* heterozygous flies compared with wild-type (*y w*) embryos (Fig. 3F).

We also looked at the nuclear localization of the heterochromatin associated protein HP1 (Kellum et al., 1995). HP1 mostly localizes in polyploid nuclei of salivary glands in a single spot that corresponds to the chromocenter (Cryderman et al., 2005), but also in chromosomal arms and the telomeres (Fig. 3G). The localization of HP1 in the nuclei of *salm/salr* knockdown salivary glands appears in several spots located in proximity to the nucleolus (Fig. 3H). Taken together, these results suggest that cells heterozygous for the *salm/salr* deficiency have defective silencing in the expression of retrotransposons and white^+^ insertions in telomeric regions, as well as alterations in the pattern of HP1 accumulation within the nucleus.

The formation of heterochromatin in pericentromeric and telomeric regions is a prerequisite for the correct segregation of chromosomes during mitosis. The *Drosophila* blastoderm is a convenient system to analyze mitotic defects, because in normal embryos nuclear divisions are synchronous (Foe, 1989). We found a variety of alterations in embryos arising from heterozygous flies for a *salm* and *salr* deficiency [*Df(2L)32FP-*5; Barrio et al., 1999], including the formation of anaphase bridges (Fig. 3I,K,L), loss of synchrony in mitotic figures (Fig. 3J), and a general disorganization of mitotic spindles (Fig. 3J). We also observed changes in the expression of histone modifications (phospho-Histone3 and H4K12ac) in 0-2 h-old embryos from *Df(2L)32FP-5/CyO* mothers (Fig. 3M-N″). The H4K12ac modification, which interferes with PH3 deposition (Boros, 2012), is expressed at very low levels in *salm/salr* mutant blastoderms (Fig. 3M,M′,N,N′). As a consequence, PH3 is detected at higher-than-normal levels in all *salm/salr* mutant preblastodermal nuclei in interphase (Fig. 3M-N″). All these data indicate that in early blastoderms the reduction in Salm/Salr protein levels is associated with changes in chromatin organization, histone modifications and the appearance of mitotic errors.

### Salm/Salr function is required for genome integrity

**Fig. 4.**
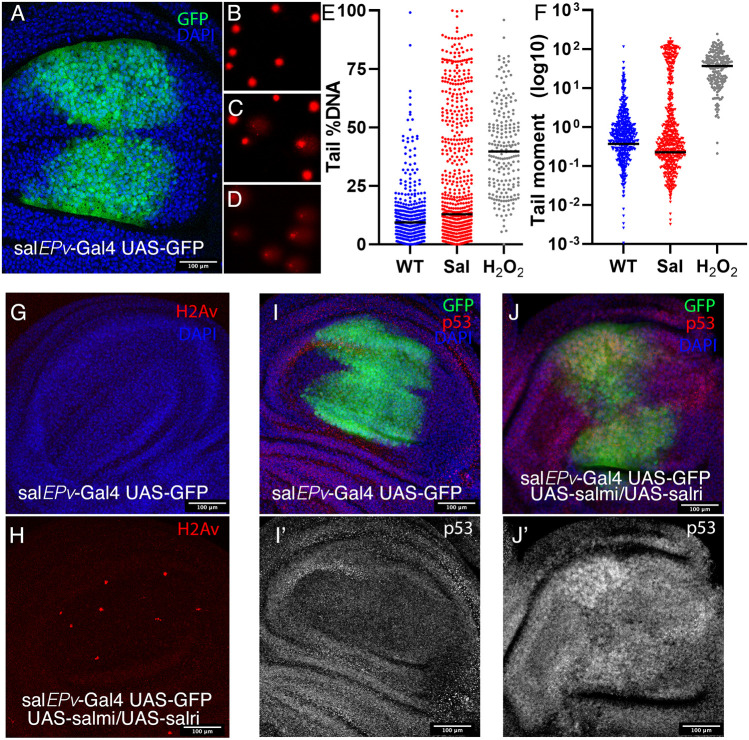
**DNA damage in *salm/salr* mutant wing imaginal discs.** (A) Expression of GFP (green) in the wing blade of *sal^EPv^-Gal4 UAS-GFP/+* late third instar wing disc. DAPI staining is shown in blue. (B-D) Representative examples of wing imaginal disc nuclei obtained from *sal^EPv^-Gal4 UAS-GFP/+* (B), *sal^EPv^-Gal UAS-GFP/UAS-salm-RNAi; UAS-salr-RNAi/+* (C) and *sal^EPv^-Gal4 UAS-GFP/+* dissociated discs treated for 10 min with H_2_O_2_ 50 µM (D). (E,F) Percentage of tail DNA (E) and value of tail moment (F) in the single cell electrophoresis assay from *sal^EPv^-Gal4 UAS-GFP/+* (blue dots), *sal^EPv^-Gal UAS-GFP/UAS-salm-RNAi; UAS-salr-RNAi/+* (red dots) and *sal^EPv^-Gal4 UAS-GFP/+* nuclei exposed to H_2_O_2_ (gray dots). Horizontal lines indicate median. (G,H) Expression of phosphorylated H2Av (red) in control (*sal^EPv^-Gal UAS-GFP/+*; G) and *sal^EPv^-Gal UAS-GFP/UAS-salm-RNAi; UAS-salr-RNAi/+* discs (H). (I-J′) Expression of p53 (red in I,J) in *sal^EPv^-Gal4 UAS-GFP/+* (I,I′) and *sal^EPv^-Gal UAS-GFP/UAS-salm-RNAi; UAS-salr-RNAi/+* discs (J,J′). GFP is in green (I,J) and DAPI staining is in blue (I,J). Individual red channels (p53) are shown in I′,J′. The confocal settings were the same in all pictures shown in panels I-J′.

Loss of heterochromatin silencing leads to DNA damage and activation of DNA replication checkpoints, altering the progression of the cell cycle (Fortuny and Polo, 2018). The wing disc is a proliferative epithelial tissue that exhibits a canonical response to DNA damage caused by irradiation (Baonza et al., 2022). Interestingly, *salm/salr* mutant wing discs show accumulation of cells in the G2 phase of the cell cycle and loss of mitotic cells (Organista and de Celis, 2013), which is compatible with DNA damage. To determine the existence of DNA damage in imaginal cells deficient for the *salm/salr* genes, we used the single cell electrophoresis assay to detect single- and double-strand DNA breaks in dissociated wing imaginal discs of the *sal^EPv^-Gal4/UAS-salm-RNAi; UAS-salr-RNAi/+* genotype. The expression of *sal^EPv^-Gal4* is restricted to the central domain of the wing blade (Fig. 4A). Consequently, cells from these discs contain *salm/salr* knockdown cells (wing cells from the domain of *salm/salr* expression), normal wing cells lacking expression of the *salm/salr* genes, and cells from the wing hinge and thorax that express *salm/salr* but do not express the corresponding RNAis. In these experiments we used wild-type discs (*sal^EPv^-Gal4 UAS-GFP/+*) as a control of background levels of DNA damage. As a positive control we used wing disc cells extracted from *sal^EPv^-Gal4 UAS-GFP/+* larvae treated with 50 µM H_2_O_2_ for 10 min before the electrophoresis, as H_2_O_2_ induces DNA double- and single-strand breaks (Driessens et al., 2009). Fluorescent imaging of DNA from single nuclei showed cells displaying DNA damage that are present in *salm/salr* knockdown discs but not in control *sal^EPv^-Gal4/UAS-GFP* discs (Fig. 4B,C). In contrast, wing disc cells treated with H_2_O_2_ showed a unique population of cells with considerable DNA damage (Fig. 4D). The quantification of the percentage of DNA in the tails (Fig. 4E), as well as the values of tail moment (Fig. 4F), a measure that incorporates the amount and the extent of DNA damage in cells, confirm the existence of DNA damage in *salm/salr* mutant discs (Fig. 4E,F). The incorporation of γ-H2AX [H2Av (His2Av) in *Drosophila*] to chromatin is a primary response to DNA double-strand breaks (Rogakou et al., 1998). We found that *salm/salr* knockdown wing imaginal discs display ectopic P-H2Av in a small fraction of wing cells (8±2 cells per wing disc), a situation never detected in wild-type wing discs of the same age (Fig. 4G,H). Because the results of the comet assay show extensive DNA damage, we suggest that most of these alterations correspond to single-strand DNA breaks that are not detected by H2Av accumulation.

A key player in the DNA damage response is the tumor suppressor p53, protein levels of which increase as a response to DNA damage (Hafner et al., 2019). Once activated by phosphorylation, p53 regulates the expression of genes related to cell division, cell death and DNA repair (Hafner et al., 2019). The expression of p53 is detected at similar levels in all cells of the wild-type wing disc (average intensity levels/area of 13.9±3.1; Fig. 4I,I′). In contrast, we found that the levels of p53 protein are increased in the central region of the wing blade in *salm/salr* mutant discs (average intensity levels/area of 20.0±4.7; Fig. 4J,J′). Taken together, our results suggest that loss of *salm/salr* function in the wing disc generates DNA damage, which could be linked to the defects in cell cycle progression observed in *salm/salr* mutant cells, and to a role of these genes in the generation or maintenance of heterochromatin.

### Loss of *salm/salr* compromises nuclear envelope morphology and results in increased nucleolar size in salivary gland polyploid cells

**Fig. 5.**
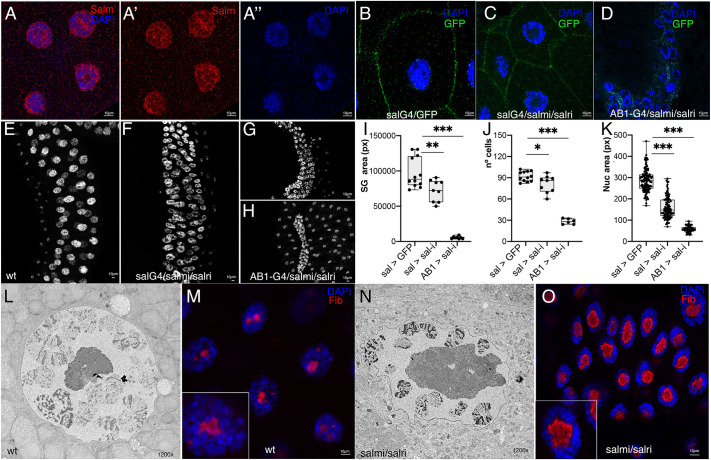
**Salivary gland phenotypes of *salm/salr* knockdown larvae.** (A-A″) Expression of Salm (red in A,A′) and DAPI staining (blue in A and A″) in wild-type salivary glands from late third instar larvae. (B-D) *sal^EPv^-Gal4 UAS-GFP/+* (B), *sal^EPv^-Gal4 UAS-GFP/UAS-salm-RNAi; UAS-salr-RNAi/+* (C) and *UAS-salm-RNAi/+; AB1-Gal4/UAS-salr-RNAi* (D) salivary gland cells (40×) showing the expression of GFP (green) and DAPI staining (blue). (E-H) Lower magnification pictures (25×) of *sal^EPv^-Gal4 UAS-GFP/+* (E), *sal^EPv^-Gal4 UAS-GFP/UAS-salm-RNAi; UAS-salr-RNAi/+* (F), *UAS-salm-RNAi/+; AB1-Gal4/UAS-salr-RNAi* female (G) and *UAS-salm-RNAi/+; AB1-Gal4/UAS-salr-RNAi* male (H). DAPI staining is in white. (I-K) Salivary gland area (I), number of cells (J) and nuclear area (K) from *sal^EPv^-Gal4 UAS-GFP/+* (sal>GFP), *sal^EPv^-Gal4 UAS-GFP/UAS-salm-RNAi; UAS-salr-RNAi/+* (sal>sal-i) and *UAS-salm-RNAi/+; AB1-Gal4/UAS-salr-RNAi* females (AB1>sal-i). *P<0.05, **P<0.01, ***P<0.001 (paired Student's *t*-test). Box plots show median values (middle bars) and first to third interquartile ranges (boxes). (L,N) TEM images of the nucleus (1200×) in *sal^EPv^-Gal4 UAS-GFP/+* (L) and *sal^EPv^-Gal4 UAS-GFP/UAS-salm-RNAi; UAS-salr-RNAi/+* (N) salivary glands. (M,O) Expression of the nucleolar marker fibrillarin (Fib; red) and DAPI staining (blue) in *sal^EPv^-Gal4 UAS-GFP/+* (M) and *sal^EPv^-Gal4 UAS-GFP/UAS-salm-RNAi; UAS-salr-RNAi/+* (O) salivary glands. The insets in M and O are magnifications (3×) showing single nuclei. Scale bars: 10 μm (A-H,M,O).

Heterochromatic regions appear to be mostly associated with the inner nuclear envelope and the periphery of the nucleolus (Bizhanova and Kaufman, 2021). In fact, heterochromatin packing contributes to the stiffness of the nuclear envelope and nucleolus morphology (Ballmer et al., 2023; Bustin and Misteli, 2016; Shevelyov, 2023). To analyze in more detail the possible effects of *salm/salr* functions on chromatin, we studied the consequences of *salm/salr* knockdowns in the salivary gland, a secretory epithelium formed by large polyploid cells (Denisa et al., 2021; Haberman et al., 2003). The spalt genes are expressed in the nuclei of all salivary gland cells (Fig. 5A), and loss of *salm/salr* function in these cells causes a variety of phenotypes, ranging from rudimentary salivary glands to morphologically normal but smaller glands showing alterations in nucleolar size and nuclear envelope morphology (Fig. 5). The combination *AB1-Gal4/UAS-salm-RNAi; UAS-salr-RNAi/+* causes the stronger phenotype, in which salivary glands are very small and formed by fewer and smaller cells than normal glands (Fig. 5B,D-F,I-K). A weaker phenotype is observed in the combination *sal^EPv^-Gal4/UAS-salm-RNAi; UAS-salr-RNAi/+* (Fig. 5B,C,E,F,I-K). We focused our analysis on the salivary glands of this last genotype, because they have an overall morphology similar to wild-type glands. Interestingly, the nuclear envelope is undulated and presents numerous indentations (Fig. 5N). In contrast, normal salivary gland nuclei display a straight nuclear envelope without major indentations (Fig. 5L). In addition, the size of the nucleolus is much larger in *salm/salr* mutants compared to normal glands, and the chromosomes have an abnormal disposition within the nuclei (Fig. 5B-D,M,O). These defects are very similar to those found in prothoracic glands mutant for *salm*/*salr* (Ostalé et al., 2024), suggesting a requirement of Salm/Salr function, at least in polyploid cells, to maintain the normal disposition of chromatin within the nucleus and to regulate morphological aspects of the nuclear envelope and nucleolus. These requirements could correspond to what has been defined as non-genetic aspects of the genome, through which chromatin physical and mechanical properties affect morphological and functional aspects of the nucleus (Bustin and Misteli, 2016).

### Irradiation and loss of Salm/Salr cause overlapping changes in gene expression in the wing disc

**Fig. 6.**
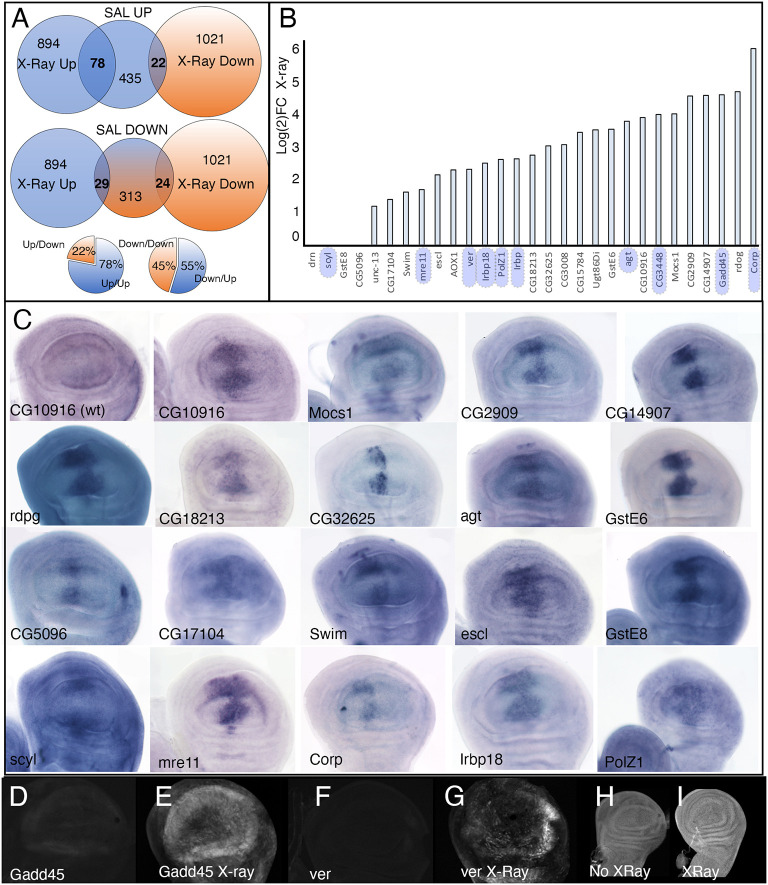
**Gene expression in *salm/salr* knockdown and irradiated wing imaginal discs.** (A) Venn diagrams showing the overlap in the genes showing expression changes in *salm/salr* knockdown discs (Organista et al., 2015) and in irradiated wing discs (van Bergeijk et al., 2012). UP and DOWN indicates genes for which expression is increased (UP) or decreased (DOWN). The percentage of overlapping genes is shown in the smaller bottom diagrams (Up/Down, Up/Up; left, and Down/Down and Down/Up; right). (B) Log(2)FC values after irradiation of wild-type wing imaginal discs (van Bergeijk et al., 2012) for genes showing ectopic expression in the central domain of *salm/salr* mutant discs. All genes within blue boxes are related to DNA damage responses. (C) Late third instar wing discs showing mRNA expression in a *sal^EPv^-Gal4/UAS-salm-RNAi; UAS-salr-RNAi* genetic background (Organista et al., 2015). Only the expression of CG10916 in wild-type wing discs is shown in the first panel. All other genes are either not expressed or expressed in all cells at low levels in wild-type wing discs. (D-G) Expression of the regulatory region of *Gadd45* (D,E) and *ver* (F,G) in third instar wing imaginal discs of *Gadd45 pHP-Dest-eGFP* and *ver pHP-Dest-eGFP* genotypes. The larvae were grown in normal conditions (D,F) or irradiated with 3000 R 2 h before dissection (E,G). (H,I) Expression of p53 in wild-type wing discs (H) and in wild-type wing discs irradiated with 3000 R 2 h before dissection (I). The confocal settings were the same in the pictures shown in panels D-G and H,I.

Our results suggest a function of Salm/Salr related to the establishment and/or maintenance of heterochromatic regions, and that a major consequence of Salm/Salr loss is the generation of DNA damage. We searched for links between the transcriptional changes observed in *salm/salr* knockdown discs [FDR 0.05, log(2)FC<−0.4 and >0.4; Organista et al., 2015] and those occurring after irradiation (van Bergeijk et al., 2012). We noticed a particular overlap for genes for which expression increases both in *salm/salr* knockdown wing discs and after irradiation. Thus 18% of genes with increased expression in *salm/salr* knockdown discs were also identified as displaying increased expression after irradiation (Fig. 6A and Table S3). We also searched for changes in expression levels after irradiation of a set of genes overexpressed in the central region of *salm/salr* mutant discs (Organista et al., 2015). Most of these genes (86%; 25/29) are also overexpressed after irradiation, with log(2)FCs in the range of 1 to 6 (Fig. 6B,C). Interestingly, many of these genes (9/25) encode proteins involved in DNA repair (Fig. 6B). The enhancer regions of two of these genes, *Gadd45* and *ver*, are active upon loss of Salm/Salr function (Ostalé et al., 2024). We found that the same enhancers are also activated after irradiation (Fig. 6D-G). The increase in expression levels observed after irradiation depends on the transcriptional regulator p53 (van Bergeijk et al., 2012), which is expressed in all wing cells at low levels even upon irradiation (Fig. 6H,I).

## DISCUSSION

We have identified cellular and genetic alterations in cells mutant for the *Drosophila salm* and *salr* genes that suggest a novel aspect of their function as transcriptional regulators. We focused mostly on the wing imaginal disc, a tissue where *salm/salr* promote cell viability, epithelial integrity and the cell cycle transition from G2 to mitosis (Organista and de Celis, 2013). It is assumed that these phenotypes are caused by misregulation of Sal target genes, but only some aspects of Sal function have been linked to the regulation of specific target genes (Milán et al., 2002; Martín et al., 2017; Ostalé et al., 2024). No candidate genes have been identified mediating the functions of Salm/Salr required to promote cell cycle progression and cell viability in the wing blade. Loss of Salm/Salr function generates a major change in the transcriptional landscape of the wing disc (Organista et al., 2015), but the relation between these changes and the functional requirements of Salm/Salr is not apparent. It is also unknown to what extent the changes in gene expression of *salm/salr* mutant discs are a consequence of Salm/Salr acting as canonical sequence-specific transcription factors.

### Loss of Salm/Salr function alters DNA accessibility in wing disc cells

New insights into the transcriptional effects of Salm/Salr loss-of-function conditions came from the analysis of chromatin accessibility and the characteristics of the Salm binding pattern to the DNA. We found that the knockdown of *salm* and *salr* genes in wing discs leads to massive changes in chromatin accessibility, which affect DNA regions preferentially located more than 5 Kb from TSSs. We also detected changes in the presence of the H3K27ac histone mark in *salm* and *salr* knockdown wing imaginal discs, but these were less numerous than alterations in chromatin accessibility, suggesting that remodeling of chromatin in enhancer sequences could be a less determining event in the transcriptional response to Salm/Salr. The overlap between the two datasets was enriched for regions that simultaneously increased in both chromatin accessibility and H3K27ac modifications, as well as for those showing the opposite change, less accessibility and less acetylation of H3K27. Consistently, we recently found that Salm binding to the DNA is enriched in pericentromeric heterochromatin, as ∼80% of chromatin bound by Salm belongs to this class (Ostalé et al., 2024). Altogether, these observations indicate that a prominent function of Salm/Salr might be related to the formation and/or maintenance of heterochromatic regions, at least in the wing imaginal disc. This aspect of *Drosophila* Sal proteins is likely conserved with their vertebrate counterparts, as human and mouse SALL1 and SALL4 proteins are preferentially bound to pericentromeric heterochromatin (Sakaki-Yumoto et al., 2006; Yamashita et al., 2007). We also found that changes in chromatin accessibility are poorly correlated to the changes in gene expression detected in *salm/salr* mutant wing discs. This observation mirrors the very limited overlap between DNA binding and mRNA expression in the case of human SALL4 (Yang et al., 2017), and the lack of correlation between changes in gene expression and chromatin accessibility in odontoblast progenitors mutant for *Sall1* during mouse tooth development (Lin et al., 2021). The poor correlations observed between global gene expression and changes in chromatin accessibility in *salm/salr* knockdown conditions suggest that loss of Sal function indirectly triggers a multitude of gene expression alterations that are not caused by direct regulation of target genes by Salm/Salr binding. A caveat to this conclusion is that our analyses have been carried out in knockdown conditions, which likely represent a partial loss of gene function, as well as the criterion of proximity to the nearest TSS used to associate the sequences identified in the ATAC and H3K27ac immunoprecipitation experiments to the affected transcript.

### Salm/Salr function and heterochromatin organization

**Fig. 7.**
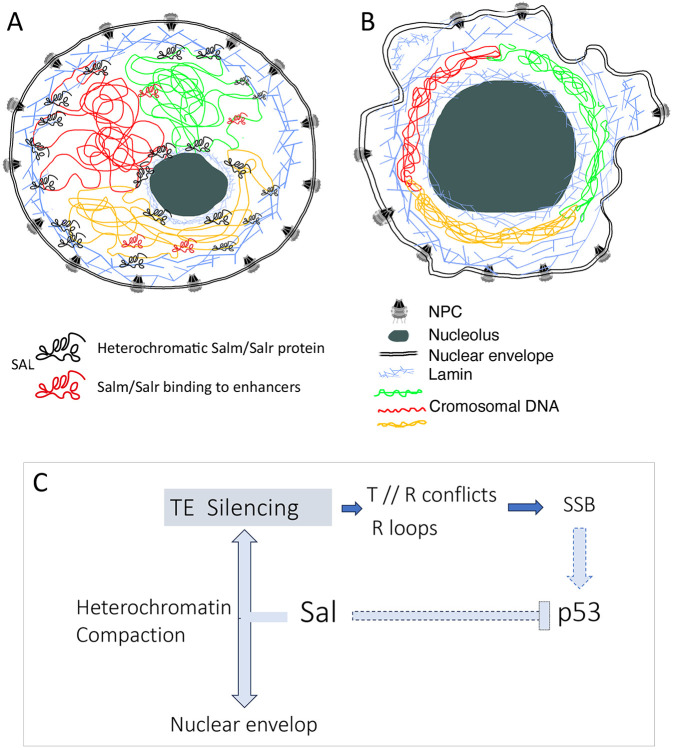
**Proposed model for Salm/Salr function.** (A,B) Cartoon representing wild-type (A) and Salm/Salr mutant (B) nuclei. The nuclear envelope is drawn as a double line showing the nuclear pore complexes (NPC). Lamins are drawn as crisscross blue lines surrounding the nuclear envelope and the nucleolus (gray round shape). Chromosomal DNA is represented by red, green and yellow lines. The Salm/Salr proteins are represented as black and red shapes, indication Salm/Salr binding to euchromatic regions and acting as a canonical sequence-specific transcription factor (red) or as a heterochromatic binding protein (black). The main alterations observed in *salm/salr* mutant polyploid cells are shown in B, including wiggly nuclear envelope, enlarged nucleolus and reorganization of nuclear DNA around the periphery of the nucleus. (C) Working hypothesis to account for the variety of genomic and cellular changes observed in *salm/salr* knockdowns or mutant conditions. We propose that loss of Salm/Salr function affects the formation or maintenance of heterochromatic regions, leading to abnormal expression of transposable elements and other heterochromatic sequences, generating transcription-replication conflicts (T//R) and/or R-loops leading to single-strand DNA breaks (SSB) and the activation of a p53 DNA damage response. We also indicate the possibility of direct repression of p53 by Salm/Salr proteins.

The formation and maintenance of heterochromatin in eukaryotic genomes is a fundamental aspect of the structural and functional organization of chromosomes within the nucleus. Constitutive heterochromatin is localized at subtelomeric regions and close to the centromeric DNA, is characterized by epigenetic marks (H3K9me2/3 and H4K36me2) and remains mostly transcriptionally inactive (Grewal, 2023; Noma et al., 2001). Facultative heterochromatin comprises genomic regions that interact with the nuclear envelope and nucleolus, where gene silencing is regulated (Fortuny and Polo, 2018). Heterochromatin is required for genome integrity, transcriptional silencing of repetitive DNA sequences and nuclear membrane stiffness (Janssen et al., 2018). Some of the alterations we found in *salm/salr* mutant cells, such as the augmented expression of *white^+^* subtelomeric insertions, can be directly ascribed to faulty heterochromatin assembly. In addition, the appearance of mitotic defects, increased expression of retrotransposons and alterations in H3 phosphorylation observed in early blastoderms derived from *sal* heterozygous flies suggest a role for Salm/Salr in the initial establishment of centromeric heterochromatin regions. Other *Drosophila* transcription factors, such as Eyegone (Eyg) and Homothorax (Hth) have been shown to participate in the regulation of heterochromatic assembly in early *Drosophila* blastoderms (Hth; Salvany et al., 2009; Zaballos et al., 2015) or in heterochromatic gene silencing (Eyg; Salvany et al., 2012; Blom-Dahl and Azpiazu, 2018). Although the variety of genetic and developmental alterations detected in diploid and polyploid *salm/salr* mutant cells points to a role of the corresponding proteins in heterochromatin formation, the molecular events linking chromatin organization and Salm/Salr function are still unexplored. However, we can envision several possible roles of Salm/Salr promoting heterochromatin formation. First, this function could be mediated by interactions with the NuRD complex. Thus, the activities of NuRD and vertebrate Sall proteins have been linked in multiple cellular contexts (Basta et al., 2017; Wang et al., 2023; Ma et al., 2018), and a function of NuRD in the assembly of pericentromeric heterochromatin has already been suggested in B cells and in *Schizosaccharomyces pombe* (Creamer et al., 2014; Chadwick et al., 2009; Sims and Wade, 2011). Furthermore, Sall4 promotes reprogramming of mouse embryonic fibroblast by recruiting NuRD to close open chromatin (Wang et al., 2023), further suggesting a relationship between Sal function and NuRD regulating chromosomal architecture. In this context, the function of Salm/Salr would imply the recruitment to genomic targets of NuRD complexes to promote heterochromatin compaction and gene silencing. Also, the function of Salm/Salr might involve interactions with lamins, which constitute the main component of the internal nuclear envelope and are also located in the nucleolus periphery. In this model, which has already been suggested for the DNA-binding protein Oct1/POU2F1 (Meuleman et al., 2013; Wang et al., 2023), Salm/Salr would participate in tethering heterochromatic DNA to lamin in both the nuclear envelope and the nucleolus periphery. In fact, nuclear lamina-associated DNA domains are regions enriched in A/T sequences (Meuleman et al., 2013), the most prominent DNA binding signature of Sal proteins (Kong et al., 2021; Yamashita et al., 2007). In both scenarios; we expect that loss of Salm/Salr activity leads to de-repression of genes, retrotransposon expression and the appearance of DNA damage (Chen et al., 2016; Nikolov and Taddei, 2015). Similarly, the changes we found in the size of the nucleolus and the circularity of the nuclear envelope in salivary glands could also be caused by defects in heterochromatin formation, or in the proper attachment of heterochromatin to the nuclear envelope or nucleolus periphery (Fig. 7).

### A function of Salm/Salr preserving genome integrity

The organization of DNA in chromatin domains has a broad impact on the maintenance of genome integrity, influencing both the susceptibility to DNA damage and the efficiency of DNA repair (Ortega et al., 2021). In fact, changes to the heterochromatin landscape can be associated with mitotic errors, aberrant chromosomal structures, replication stress and replication-transcription conflicts associated with increased transposable elements transcription (Janssen et al., 2018). *Salm/salr* mutant cells display low levels of apoptosis and are preferentially stalled in the G2 phase of the cell cycle (Organista and de Celis, 2013), two cellular phenotypes that we can now relate to the generation of DNA damage in these cells. Thus, we were able to visualize DNA damage in *salm/salr* mutant cells, and also detected increased expression of p53 in these cells. Consistently, there is a considerable overlap between the transcriptional response to irradiation and the changes in gene expression observed in *salm/salr* mutant wing discs. We have not explored the mechanistic details leading to the activation of a p53-dependent DNA damage response in *salm/salr* mutant cells. However, considering other aspects of the *salm/salr* phenotype, we suggest that DNA damage occurs as a consequence of the function of Salm/Salr proteins in the maintenance of heterochromatic domains. Interestingly, several links between Sal function and DNA damage have already been identified, including the induction by Sall4 of resistance to irradiation in a p53-dependent manner (Nie et al., 2019). The interrelationships between DNA integrity and Sal proteins could be a determining factor in the function of these genes in the development of embryonic stem cells and human cancers associated with altered expression of human Sall genes (Nie et al., 2019; Xiong et al., 2015).

The previously unreported functions we are suggesting for *Drosophila* Sal proteins in the maintenance of heterochromatic domains and genome stability are likely required in all cells. However, the expression of the sal genes in the wing disc is restricted to particular areas, such as the central region of the wing pouch, the wing hinge and the presumptive lateral thorax. This raises an intriguing caveat, related to how the functions of Sal proteins are exerted in cells where the corresponding genes are not expressed. At this time, we can only speculate that perhaps other DNA binding proteins might be able to perform similar molecular functions related to chromatin biology in cells where Sal proteins are not expressed.

## MATERIALS AND METHODS

### *Drosophila melanogaster* cultures

Flies were kept at 17°C or 25°C in temperature controlled rooms with 55% humidity. We used standard fly food containing glucose, agar, yeast, propionic acid and flour. We used the Gal4/UAS system (Brand and Perrimon, 1993) to express *salm* and *salr* RNAi (*UAS-salm-RNAi*; VDRC3029 and *UAS-salr-RNAi*; VDRC28386, Vienna *Drosophila* Resource Center) in the wing disc (*sal^EPv^-Gal4*; Cruz et al., 2009) and the salivary gland drivers *AB1-Gal4* (BL1824 from the Bloomington Drosophila Stock Center) and *sal^EPv^-Gal4* (Cruz et al., 2009). The expression of *UAS-salm-RNAi* strongly reduces Salm expression (Ostalé et al., 2024), and the knockdown of *salm* and *salr* in the wing disc results in wing phenotypes similar to those associated to a *salm/salr* deficiency (Organista and de Celis, 2013), indicating that these lines are very effective to generate *salm* and *salr* knockdowns. We also used UAS-mCD8-GFP (BL5137 from the Bloomington Drosophila Stock Center) and the 2R and 3L mini *w^+^* subtelomeric insertions P[+mW.hs] (BL44260 and BL44261 from the Bloomington Drosophila Stock Center)*.* We used a chromosomal deficiency for the *salm* and *salr* genes [Df*(2L)32FP5*; Barrio et al., 1999]. This deficiency is late embryonic and early L1 larval lethal in homozygosity (Ferreiro et al., 2012). We also used the reporter constructs *Gadd45 pHP-Dest-eGFP* and *ver pHP-Dest-eGFP* (Ostalé et al., 2024).

### Irradiation of third instar larvae

*Gadd45 pHP-Dest-eGFP*, *ver pHP-Dest-eGFP* and wild-type third instar larvae were irradiated (4000R) in a Phillips MG102 X-ray machine, the wing discs were dissected 2 h after irradiation, and the expression of GFP and p53 was visualized in the confocal microscope with the same settings of intensity levels and pinhole for both control and irradiated discs.

### Gene expression analyses

We selected a dataset of 748 genes for which expression changes [±1.5 log (2)FC; *P*<0.003], comparing imaginal wing discs of *sal^EPv^-Gal4 UAS-GFP; tub-Gal80^ts^/UAS-GFP* and *sal^EPv^-Gal4 UAS-GFP/UAS-salm-i; tub-Gal80^ts^/UAS-salr-i* genotypes grown for 24 or 48 h at 29°C before dissection in the third larval instar (Organista et al., 2015). The expression of these genes was either increased (435 genes) or decreased (313 genes) at both time points (152 and 153, respectively) or only after 24 h (33 and 51) or 48 h (250 and 109) (Table S1). Wing imaginal disc *in situ* hybridization data in wild type and *sal^EPv^-Gal4 UAS-GFP/UAS-salm-i; UAS-salr-i*/+ genotype were obtained from Organista et al. (2015; figures S3 to S13).

We also selected a set of genes for which expression in the wing disc changes ±1.5-fold [*P*<0.005; ±0.585 log (2) FC] 2 or 18 h after 4000R irradiation (van Bergeijk et al., 2012). This set of genes was taken from Table S1 of van Bergeijk et al. (2012), and was kindly provided by Dr Tin Tin Su. We selected 894 and 1021 genes as overexpressed or repressed, respectively, after irradiation (Table S2). The changes in gene expression observed after irradiation for ∼80% of the genes depended on the presence of *p53* (van Bergeijk et al., 2012). Finally, to determine gene expression in wild-type wing discs, we used RNA-seq data from wing imaginal discs expressing Gal4/GFP (run SRR3478156; Flegel et al., 2016) quantified using Sailfish 0.7.6.0 running on the Galaxy platform. All these datasets (*salm/salr* knockdown RNA-seq, X-ray RNA-seq and the *salm/salr* knockdowns ATAC and H3K27ac ChIP) were compared with each other looking for similarities, even though they were extracted in different experimental settings.

### Transposable elements expression analysis

We used real time PCR, performed with a Bio-Rad CFX Opus 384 machine, to quantify the amount of *F-element*, *TART-A*, *Het-A* and *Rpl32* mRNA in normal and *salm/salr* deficient embryos. RNA from 0-2 h-old embryos from Df*(2L)32FP5*/CyO and wild-type parents was extracted using the NZYTotal RNA isolation kit (MB13402). We used wild-type embryos as controls, as there is no likely effect of balancer chromosomes (CyO) on the expression of retrotransposons (Ghavi-Helm et al., 2020). Then 1 µg of total RNA was treated with DNAaseI (from the NZYTotal RNA isolation kit) at 37°C for 30 min and retrotranscribed with the Super Script III Reverse Transcriptase (Invitrogen).

The primers we used were: F-Element Fwd, 5′-AGATCCGGCAGACATTCAG-3′; F-Element Rv, 5′-ACTTGACCATGTTTCCCCC-3′; TART-A Fwd, 5′-TCTCCTCAGACATGTCCCTCCCAT-3′; TART-A Rv, 5′-TCTTGTAGCGGCAGTTGCTAGTGT-5′; Het-A Fwd, 5′-TTGTCTTCTCCTCCGTCCACC-3′; Het-A Rv, 5′-GAGCTGAGATTTTTCTCTATGCTACTG-3′; Rpl32 Fwd, 5′-AGCATACAGGCCCAAGATCG-3′; Rpl32 Rv, 3′-TGTTGTCGATACCCTTGGGC-3′.

### Eye color assays and quantification of eye pigment

To analyze gene expression in heterochromatic regions we monitored the expression of the gene *white* (*w*) present in *P(w+)* subtelomeric insertions located in the 2R and 3R chromosomes. *w-; Df(2L)32FP-5/CyO* flies are white-eyed. When crossed with *w; P(w+)* flies, the red eye color in the w; *Df(2L)32FP-5/*P*(w+)* or w; *CyO/*P*(w+)* progeny comes exclusively from the *white* mini gene expressed from the *P(w+)* insertion. This means that the intensity of red color observed in each genotype depends on the levels of *white* expressed from the P element. We measured pigments from 15 female heads of each genotype. The heads were frozen at −20 for 5 days and then homogenized in methanol acidified with 0.1 M HCl (1:9) to extract the pigment. The extracted pigment was measured at 480 nm. Three independent extractions were performed for each genotype.

### ATAC-seq

For ATAC-seq experiments, two replicates of 20 imaginal wing discs from late third instar larvae were used per sample: the larvae were of *sal^EPV^-Gal4 UAS-GFP/UAS-salmi-; UAS-salr-i/+* and *sal^EPV^-Gal4 UAS-GFP/+* genotypes. The discs were dissected in pre-cooled PBS and pipetted in PBT (PBS with 0.1% Triton X-100) containing 1× protease inhibitors (cOmplete™ Protease Inhibitor Cocktail tablets, Roche, 11873580001). The disks were incubated in PBT with 1× protease inhibitors and 0.4% IGEPAL CO-630 (Sigma-Aldrich, 542334) for 30 m on a nutator at 4°C. The suspension was centrifuged for 5 m at 4°C at 3200 ***g***. The pellet was resuspended in transposition solution containing 5 µl TDE1 (Illumina, 15027865), 25 µl 2× buffer (Illumina, 15027866), 20 µl nuclease-free H_2_O and incubated for 30 m at 37°C. We then added 50 µl of stop solution [50 mM Tris-HCl (pH 8.0), 100 mM NaCl, 0.1% SDS, 100 mM EDTA (pH 8.0)] and 5 µl of RNAse at 1 mg/ml, followed by an incubation of 10 m at 55°C. Finally, we added 3 µl of Proteinase K (20 mg/ml) and incubated for 1 h at 65°C. The DNA was purified using the DNA clean and concentrator kit (Zymo, D4014) and the columns were eluted twice in 12 µl of 10 mM Tris-Cl buffer (pH 8.5). The DNA concentration was quantified with Qubit (Invitrogen). The DNA was amplified by PCR using the Nextera DNA sample preparation and indexing kits (Illumina, FC-121-1030, FC-121-1011) as follows: 20 ng of DNA, 2.5 µl of i5, 2.5 µl of i7 oligonucleotides, 2.5 µl Nextera cocktail and 7.5 µl of Nextera PCR mastermix (NPM, Illumina, 15027920) in a total volume of 25 µl. The PCR amplification program was 72°C 3 min, 98°C 30 s, then 12 cycles of 98°C 10 s, 63°C 30 s, 72°C 3 min. The DNA fragments were size-selected with AMPure XP beads using a double-sided size selection with a right-side clean-up ratio of 0.5×, and a left-side clean-up ratio of 1.8× (Beckman Coulter, A63881). The two step clean-up was performed as described by the manufacturer. DNA was eluted in a final volume of 15 µl. The samples were sequenced with Illumina NextSeq500, 75 bp paired-end to a depth of 50 M-90 M reads at the EMBL GeneCore (Fig. S1). The correlation matrix of ATAC-seq samples, the overall enrichment of sequences in *sal^EPv^-Gal4 UAS-GFP/UAS-salm-i; UAS-salr-i/+* versus *sal^EPv^-Gal4 UAS-GFP/*+, as well as the sequence alignments of all ATAC-seq and H3K27ac Ch IP-seq experiments are shown in Fig. S1.

### H3K27ac chromatin immunoprecipitation

For ChIP-seq experiments, 120 imaginal discs from late L3 larvae were used per sample. The larval genotypes were *sal^EPV^-Gal4 UAS-GFP/UAS-salmi-; UAS-salr-i/+* and *sal^EPV^-Gal4 UAS-GFP/+*. Discs were fixed in 1.8% formaldehyde, snap frozen and stored at −80 until required.

#### Chromatin preparation from frozen *Drosophila* imaginal wing discs

Frozen disks were thawed on ice and added to 500 µl RIPA buffer [140 mM NaCl, 10 mM Tris-HCl (pH 8.0), 1 mM EDTA, 1% Triton X-100, 0.1% SDS, 0.1% Na-deoxycholate] and 1× Roche cOmplete protease inhibitors. The samples were then sonicated in the Bioruptor Pico using 1.5 ml sonication tubes (Diagenode, C30010016) according to the manufacturer's instructions for 12 cycles (30 s on/30 s off). The supernatant was transferred into 1.5 ml low binding tubes (Eppendorf, 0030108051) and centrifuged at 20,000 ***g*** for 10 m at 4°C. For quality control and quantification of the chromatin, an aliquot of 10 µl was taken out and processed as described below. The rest of the supernatant was snap-frozen in liquid N2 and stored at −80°C until the ChIP was performed. For quality control of the chromatin, one aliquot of 10 µl was RNase treated (50 µg/ml final) at 37°C for 30 m and reverse cross-linked using a final concentration of 0.5% SDS and 0.5 mg/ml Proteinase K at 37°C for 10 h and 65°C for 8 h on a thermomixer with interval shaking. The next day, the DNA was purified with phenol-chloroform purification and precipitated with ethanol, sodium acetate (pH 5.3) and glycogen to obtain pure DNA. The size distribution was assessed by gel electrophoresis through running the DNA on a 1.2% agarose TAE gel. The majority of the DNA was concentrated between 250 and 500 bp. The concentration was measured using Qubit hs DNA (Thermo Fisher Scientific, Q33230).

#### H3K27ac ChIP-seq

ChIP-seq was performed as described in Bonn et al. (2012). We incubated 1 µl of H3K27ac antibody (AbCam, ab4729, 1:100) overnight with the chromatin in RIPA buffer in a total volume of 900 µl. The next day, 25 µl of magnetic protein A/G beads (Dynabeads, Invitrogen, 10002D and 10004D) were washed with 1 ml of RIPA buffer and added to the immunoprecipitations for an additional 3 h incubation on the rotating wheel at 4°C. The ChIPs were then washed for 10 min on the rotating wheel with 1×1 ml RIPA, 4×1 ml RIPA-500 [500 mM NaCl, 10 mM Tris-HCl (pH 8.0), 1 mM EDTA, 1% Triton X-100, 0.1% SDS, 0.1% Na-deoxycholate], 1×1 ml LiCL buffer [250 mM LiCl, 10 mM Tris-HCl (pH 8.0), 1 mM EDTA, 0.5% IGEPAL CA-630 CA-630, 0.5% Na-deoxycholate] and 2×1 ml TE buffer [10 mM Tris (pH 8.0), 1 mM EDTA] in the cold room. The chromatin was then RNase-treated and reverse cross-linked as previously described for the quality check of the chromatin. The molecular barcoded ChIP-seq library was prepared with all ChIPed-DNA obtained using the NEBNext UltraII DNA library Prep kit (New England Biolabs, E7645S). The quality of the libraries was assessed on a Bioanalyzer (Agilent), and libraries displayed a peak at around 350-600 bp. ChIP-seq libraries were paired-end sequenced with 75 bp paired-end reads using Illumina NextSeq 500 platform at the EMBL Genomics Core Facility.

### Determination of pericentromeric regions

The extent of pericentromeric regions within the entire *Drosophila* genome was based on the epigenetic boundaries defined by Riddle et al. (2011) between euchromatin and heterochromatin, according to the locations of abrupt transitions in H3K9 di-methylation (H3K9me2) on the X chromosome and autosomes 1, 2 and 3. We considered the entire fourth chromosome as formed by pericentromeric chromatin throughout its length. For the 3R chromosome arm, which lacks an H3K9me2 epigenetic barrier, we used the cytological barrier established by Hoskins et al. (2007). Given that the annotated length for these heterochromatin sequences (annotation release 6, dm6) is ∼19 million bases (19 Mb), and the total genomic length with annotated sequences is ∼134 Mb (133,880,608 bp, excluding the Y chromosome as we did not distinguish by sex in the experimental design), we determined that 14.1% of the total genome length corresponds to pericentromeric chromatin.

### Calculation of the overlap between the ATAC and H3K27ac datasets with pericentromeric chromatin and with HP1a and H3K9me3 ChIP datasets

The coordinates for the sequences bound to HP1 or modified with H3K9me3 were obtained from ChIP experiments conducted by G. Karpen (Brigham and Women's Hospital, Harvard Medical School, Boston, MA, USA; modENCODE ID 4936 and 4952, respectively). We used the total sequences obtained in these experiments to establish the percentage of the total sequenced genome that is bound to HP1 (11.8%) or modified with H3K9me3 (14.5%), the sequences and peaks included within pericentromeric heterochromatin (Table S2) and the overlap between the ATAC and H3K27ac datasets with the HP1 and H3K9me3 datasets (Table S2).

### Transmission electron microscopy

Third instar larvae of *sal^EPv^-Gal4 UAS-GFP/+* (25°C) and *sal^EPv^-Gal4 UAS-GFP/UAS-salm-i; UAS-salr-i*/+ (25°C) genotypes were fixed in formaldehyde:glutaraldehyde (4%:0.04%) for 2 h at room temperature and then kept at 4°C for at least 4 days. The ring gland and the salivary gland were placed in epoxy resin, sectioned with an ultramicrotome, and the images taken using a JEOL JEM1010 transmission electron microscope at 80 kV with a TemCam F416 camera and EMenu software.

### Immunohistochemistry

Imaginal discs and salivary glands were dissected in PBS, fixed for 20 min in PBS/Triton X-100 0.3%, pre-absorbed for 2 h in PBS/Triton X-100 0.3%/bovine serum albumin 0.5% and incubated overnight in primary antibodies. We used rabbit anti-Salm (de Celis et al., 1996; 1:50), mouse anti-Fibrilarin (Sigma-Aldrich, 1:1000), anti-HP1 (c1a9, ID: AB_528276; 1:500) and anti-H2Av (Clone ID UNC93-5.2.1, ID: AB_2618077; 1:500) from the Hybridoma Bank at Iowa University, anti-p53 (Santa Cruz Biotechnology, sc-25767, 1:100), anti-pH3 (Merck, 06-570, 1:500), anti-H4K12ac (Active Motif, 61527, 1:100) and DAPI (Merck). Secondary antibodies were from Thermo Fisher Scientific (used at 1/200 dilution). Confocal images were captured using a LSM710 confocal microscope. All images were processed with the program ImageJ2 v2.3.0/1.53q (National Institutes of Health) and Adobe Photoshop 24.7.0.

### Comet assay for wing imaginal disc cells

A total of 40 wing imaginal discs per replicate were dissected in pre-cooled PBS and incubated for 20 min in TrypLE™ (Thermo Fisher Scientific) at 37°C. We added PBS (1:1) and centrifugated at 200 ***g*** for 10 min. The discs were resuspended and washed in PBS twice and stored at −80°C in a citrate buffer pH 6.0 (Merck, C9999-100ML) containing 10% DMSO until use. Approximately 10^4^ cells were embedded in low melting agarose (0.6%) and deposited on precoated slides with 1% agarose. After agarose solidification (10 min on ice), the samples were incubated overnight at 4°C in a lysis buffer containing 2.5 M NaCl, 100 mM EDTA, 10 mM Tris, 1% Triton X-100 (pH 10). The DNA was allowed to unwind for 40 min in alkaline buffer [300 mM NaOH, 1 mM EDTA (pH>13)] and the electrophoresis was carried at 0.73 V/cm. Slides were neutralized in PBS and stained with GelRed (Thermo Fisher Scientific). Samples were examined using an Olympus BX-61 microscope equipped with an Olympus DP70 camara to capture 20-25 field images that were scored with the free CometScore 2.0 software (TriTek) to obtain the percentage of tail DNA of at least 200 nucleoids per mini-gel. We represented DNA damage using both tail intensity (percentage of DNA in comet tails) and tail moment (the product of tail length and tail intensity), to provide a comprehensive assessment of the changes observed in DNA migration.

We compared control discs (*sal^EPv^-Gal4 UAS-GFP/+*) with sal mutant discs (*sal^EPv^-Gal4 UAS-GFP/UAS-salm-RNAi; UAS-salr-RNAi/+*) and used as positive controls slides of gel-embedded *sal^EPv^-Gal4 UAS-GFP/+* wing disc nuclei submerged into H_2_O_2_ 50 µM for 10 min. In these conditions, H_2_O_2_ induces strand breaks to create DNA comet tails of suitable size. Because loss of Sal function in the wing blade causes a low level of apoptosis (Organista and de Celis, 2013), we conducted preliminary Trypan Blue assays to confirm that the proportion of positive cells did not exceed 10%. Additionally, comets showing ‘hedgehogs’, ‘clouds’ or ‘ghosts’, which are generally associated with dying cells (Olive et al., 1993), were routinely excluded during scoring to prevent biases related to cell death. We conducted two independent experiments that included all analyzed conditions, with each sample containing at least 40 wing imaginal discs. Two technical replicates were performed per sample, scoring a minimum of 100 comets per gel, for a total of 200 comets per slide.

### Bioinformatic analyses

The analysis of H3K27ac and ATAC was performed using the Galaxy platform. Paired-end reads were cleaned for Nextera adaptors using Trimmomatic (Galaxy Version 0.36.6, with options ILLUMINACLIP: NexteraPE-PE.fa:2:30:10:8:true, SLIDINGWINDOW:4:20, MINLEN:20, SLIDINGWINDOW:4:20) for ATAC-seq and using Trim Galore! (Galaxy Version 0.4.3.1 with automatic adaptor detection, Trim low-quality ends from threshold: 20, Overlap with adapter sequence required to trim a sequence: 2, Maximum allowed error rate: 0.1, reads becoming shorter than 20 were discarded) for ChIP-seq (retrieved from https://www.bioinformatics.babraham.ac.uk/projects/trim_galore/.

The remainder of the workflow is common to ATAC-seq and ChIP-seq. Trimmed reads were mapped to the *Drosophila* reference genome dm6 version (the Berkeley *Drosophila* Genome Project; BDGP) with Bowtie2 (Langmead and Salzberg, 2012; Galaxy Version 2.3.4.3+galaxy0, with options –fr -I 0 -X 2000 –sensitive –dovetail). Mapped reads were then filtered using Filter SAM or BAM, output SAM or BAM files on FLAG MAPQ RG LN or by region (Galaxy Version 1.1.2) to only keep read mapped in a proper pair with MAPQ>19 (which eliminates multi-mapping reads). We also only kept read pairs that mapped to major chromosomes (chr2L, chr2R, chr3L, chr3R, chr4 and chrX) and removed duplicate reads using Picard MarkDuplicates (Galaxy Version 2.7.1.1; retrieved from http://broadinstitute.github.io/picard/). We created ‘25 bp 1X-normalized’ BigWig Signal Files using DeepTools bamCoverage (Galaxy Version 3.0.2.0) at a 25 bp resolution with the normalized to 1× coverage option. Quality was assessed using different tools. FastQC (Galaxy Version 0.69; retrieved from http://www.bioinformatics.babraham.ac.uk/projects/fastqc/) was run at different steps of the workflow to check sequencing quality and monitor filtering step efficiency. Picard Collect Alignment Summary Metrics (Galaxy Version 2.7.1.1; retrieved from http://broadinstitute.github.io/picard/) was used to evaluate library quality across samples (read duplication and unmapped reads rates) and Picard CollectInsertSizeMetrics (Galaxy Version 2.7.1.0; retrieved from http://broadinstitute.github.io/picard/) to compare fragment length. Finally, Deeptools plotFingerprint (Galaxy Version 3.0.2.0) was used to check ChIP signal strength. The DiffBind R package (http://bioconductor.org/packages/release/bioc/vignettes/DiffBind/inst/doc/DiffBind.pdf) was used to perform differential binding analysis between controls and *salm/salr* knockdowns using ChIP-seq peaks called with MACS2 callpeak (Galaxy Version 2.1.1.20160309.5, with options: --call-summits, q-value 0.1. and default paired-end options) performed on the filtered immunoprecipitation BAM files (uniquely mapped reads) and their matched input BAM as control. Sequence comparisons between samples and replicates was carried out using the Galaxy platform (Galaxy Community, 2022). We used the databases ORegAnno (Lesurf et al., 2016), RefFlat (Universidad de California, Santa Cruz, CA, USA; http://genome.ucsc.edu/goldenPath), and the following ModENCODE datasets: ChIP-Seq H3K9me3 (Oregon L3, Karpen, G. Dataset ID: 4952 GSE47258) and ChIP-Seq HP1 (Oregon L3, Karpen, G. Dataset ID: 4936 GSE47243).

### Correspondence between epigenetic modifications observed in *salm/salr* knockdown discs and candidate affected genes

In order to associate the sequences enriched in ATAC-seq and H3K27ac ChIP-seq with genes, we first used the database ORegAnno (Lesurf et al., 2016), which includes 4242 regulatory regions associated to specific genes. For all those regions without ORegAnno annotations, we used a criterium of proximity (van Arensbergen et al., 2014; Zabidi and Stark, 2016). We classified our sequences as belonging to proximal or distal regions when they are located less or more than 5 Kb, respectively, to a TSS.

### Statistical analyses

Comparisons between measures were analyzed using a paired two-tailed Student's *t*-test. The *P*-values were grouped as indicating moderately significant difference (*P<0.05), significant (**P<0.01) and highly significant (***P<0.001 and ****P<0.0001), indicating confidence values of 95%, 99% and 99.9%, respectively. In all genomic analyses the *P*-values were corrected using false discovery rates (FDR). The values in Fig. 2 were analyzed by chi-squared (Table S2).

## Supplementary Material



10.1242/develop.204258_sup1 Supplementary information

Table S1. Genomic regions and genes identified in gene expression analyses

Table S2. Overlap between sequences and peaks identified in our ATAC and H3K27ac ChIP experiments and pericentromeric heterochromatin, HP1 binding and H3K9me3 modifications.

Table S3. Overlap between genes identified in sal RNAseq and irradiation in the wing disc
