# Supplementary material for: A function of Spalt proteins in heterochromatin organization and maintenance of genomic DNA integrity
Source: Development. 2025 May 16;152(10):dev204258. doi: 10.1242/dev.204258 (PMC12091872; doi:10.1242/dev.204258)
Supplement: Supplementary information [file develop-152-204258-s1.pdf]

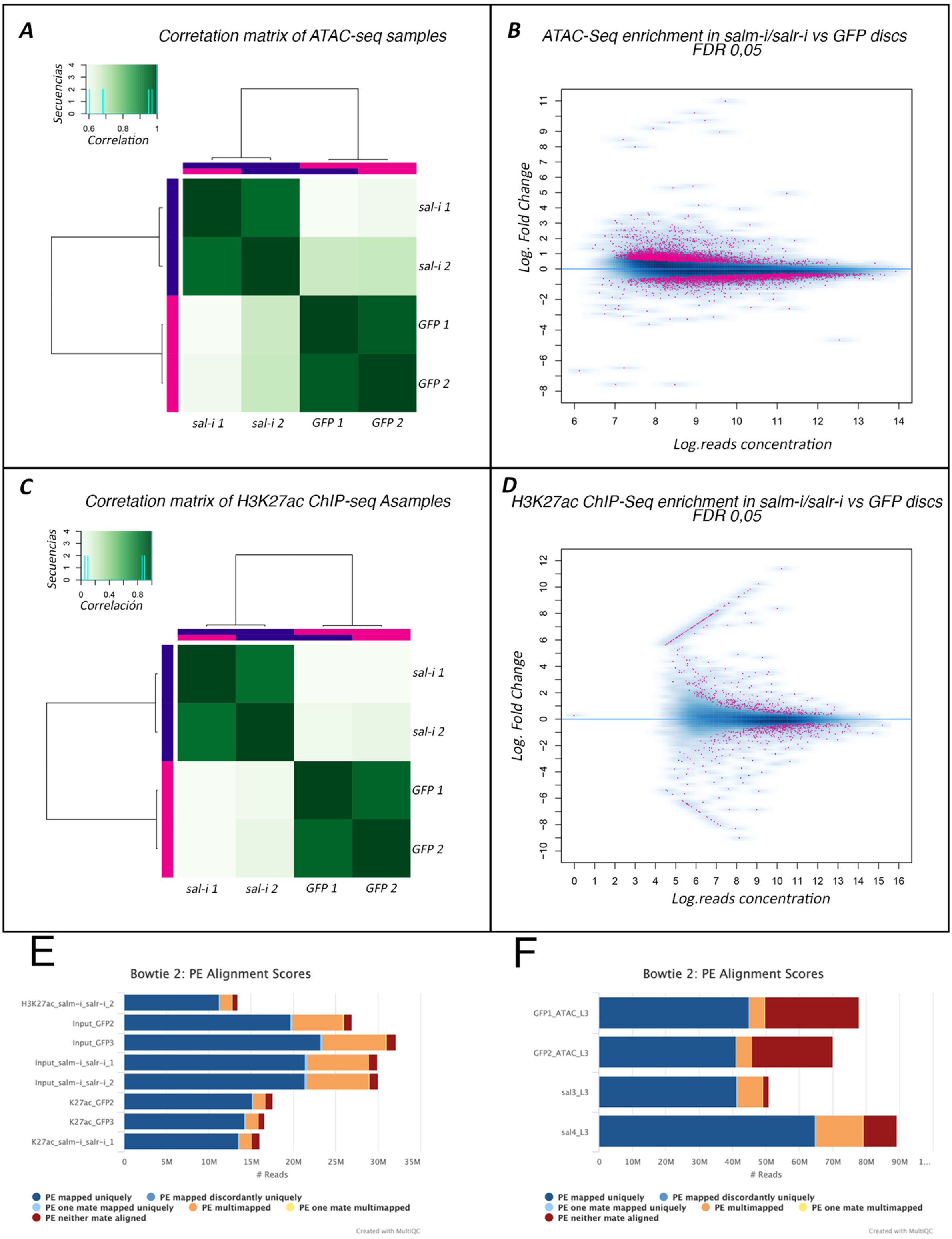

Fig. S1. (A, C) Correlation between replicates of ATAC-seq (A) and H3K27ac CHIP (C) in control (GFP 1 and GFP 2; genotype *sal^{EPv}-Gal4 UAS-GFP/+*) and *sal* mutant (sal-i 1 and sal-i 2; genotype *sal^{EPv}-Gal4 UAS-GFP/UAS-salm-RNAi; UAS-salr-RNAi/+*) samples. (B, D) MA graphics representing the correlation between the magnitudes log Fold Change and log number of reads in the genotype *sal^{EPv}-Gal4 UAS-GFP/UAS-salm-RNAi; UAS-salr-RNAi/+* compared to controls *sal^{EPv}-Gal4 UAS-GFP/+*. Blue and pink dots represent non- significant (blue) and significant (pink) changes. (E-F) Alignment scores for all H3K27ac seq (E) and ATAC-seq (F) experimental samples.

Development • Supplementary information

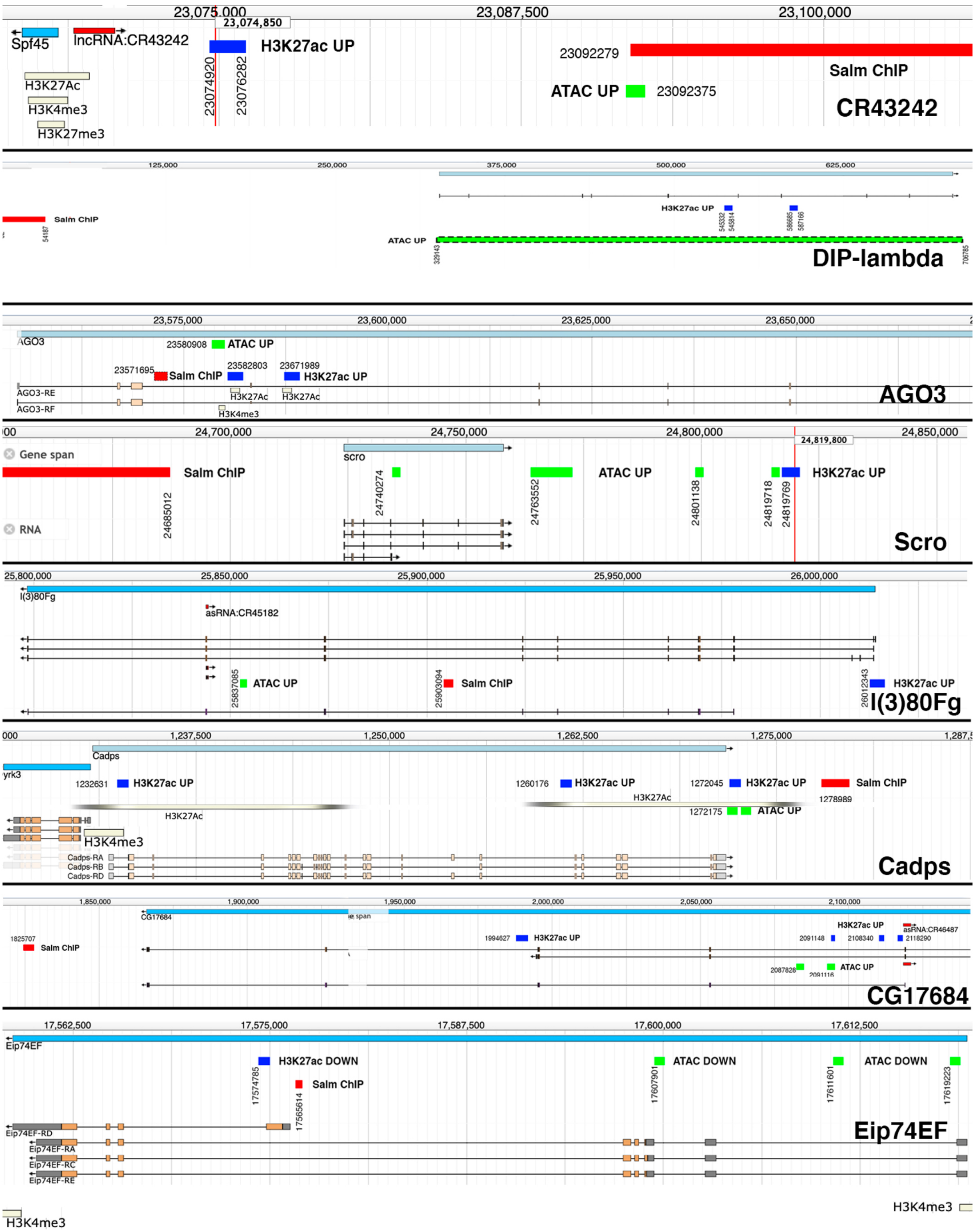

Development • Supplementary information

Fig. S2. (A) Examples of the 8 genes associated to binding of Salm (red bars) in wild type wing imaginal discs, and to changes in ATAC (green bars) and H3K27ac binding (blue bars) comparing *sal^{EPv}-Gal4 UAS-GFP/+* controls and *sal^{EPv}-Gal4 UAS-GFP/UAS-salm-RNAi; UAS-salr-RNAi/+* wing imaginal discs. The genomic maps were obtained from the flybase genomic browser.

Development • Supplementary information

Table S1. Genomic regions and genes iden-fied in gene expression analyses

Sal 24-48 tab: all genes showing significant change in expression levels; Organista et al., 2015)

sal 24-48 lgFC tab: all genes showing significant change in expression levels and logFC > 0.5; Organista et al., 2015).

ATAC_UP tab: all genomic regions identified as over-represented in the ATAC experiments (FDR<0.001).

ATAC_UP genes: all genes associated to at least one ATAC_UP sequence.

ATAC_DOWN tab: all genomic regions identified as under-represented in the ATAC experiments (FDR<0.001).

ATAC_DOWN genes: all genes associated to at least one ATAC_DOWN sequence.

H3K27ac_UP tab: all genomic regions identified as over-represented in the H3K27ac chromatin immunoprecipitation experiments (FDR<0.05).

H3K27ac_UP genes: all genes associated to at least one H3K27ac_UP sequence.

H3K27ac-DOWN tab: all genomic regions identified as under-represented in the H3K27ac chromatin immunoprecipitation experiments (FDR<0.05).

H3K27ac-DOWN genes tab: all genes associated to at least one H3K27ac_DOWN sequence (FDR<0.05).

salm ChIP tab: all genomic regions identified in the Salm chromatin immunoprecipitati on experiments Ostalé et al., 2024).

salm ChIP genes tab: all genes associated to at least one salm ChIP sequence.

Development • Supplementary information

Other symbols are:

Chr: Chromosomic arm

TSS (kb): Distance in bp to the nearest transcription start site

Gen: More proximal gene

Exp: Gene expression in the third instar disc positive (Y), negative (N) or not evaluated (NC) following RNA-seq data (Flegel et al. 2016; reads from run SRR3478156) and microarray data (GeneChip™ Drosophila Genome 2.0 Array; Affymetrix) obtained from mRNA extracted from dissected third instar wing imaginal discs (Organista et al. 2015). ATAC: Genes identified in the ATAC experiments as underrepresented (DOWN) or unrepresented (UP). HP1a and H3K9me3: Overlap for the chromosomal marks HP1a and H3K9me3.

Available for download at
https://journals.biologists.com/dev/article-lookup/doi/10.1242/dev.204258#supplementary-data

Table S2. Overlap between sequences and peaks identified in our ATAC and H3K27ac ChIP experiments and pericentromeric heterochromatin, HP1 binding and H3K9me3 modifications.

Peaks: number of peaks identified for each experiment

Bp: Total number of bp

Pericentromeric peaks: number of peaks located in pericentromeric heterochromatin

Bp pericentromeric: Total number of bp located in pericentromeric heterochromatin

The comparisons between different values was made by squared Chi test.

Available for download at
https://journals.biologists.com/dev/article-lookup/doi/10.1242/dev.204258#supplementary-data

Table S3. Overlap between genes identified in sal RNAseq and irradiation in the wing disc Data from Organista et al., 2015 and van Bergeijk et al., 2012

SAL_UP and SAL_DOWN tabs: List of genes whose expression changes in salm/salr knockdown discs compared to normal discs. We considered changes of expression detected 24 h (SAL_UP 24) and 48 hours (SAL_UP 48) after the transfer to 29ºC of controls (tub-Gal80^{ts}/+; sal^{EPV}-Gal4 UAS-GFP/UAS-GFP) and salm/salr knockdown (tub-Gal80^{ts}/+; sal^{EPv}-Gal4 UAS-GFP/UAS-salm-RNAi; UAS-salr-RNAi/+). Genes which expression increases or decreases in salm/salr knockdown discs compared to normal discs are in the tabs SAL_UP and SAL_DOWN, respectively. These data are from Organista et al., 2015.

RX_UP and RX_DOWN tabs: List of genes which expression increases (RX_UP) or decreases (RX_DOWN) in the wing disc as a consequence of DNA damage induced after irradiation (van Bergeijk et al., 2012). Columns B and C are changes 2h after irradiation and columns D and E 18 h after irradiation. Data taken from van Bergeijk et al., 2012 and provided to us by Dr Tin Tin Su.

RX P53 (van Bergeijk et al., 2012) tab: List of genes which expression changes in irradiated wild type wing disc after 2 h (RX 2) and 18 h (RX 18), and in irradiated *p53* mutant wing discs (p53 RX 2 and p53 RX 18). Expression changes are expressed in LOG(2)FC values, and significant changes (p<0.005, +/-1.5 LOG(2)FC) are indicated by asterisks.

Available for download at
https://journals.biologists.com/dev/article-lookup/doi/10.1242/dev.204258#supplementary-data

Development • Supplementary information

References

Flegel, K., Grushko, O., Bolin, K., Griggs, E. and Bulma, L., 2016. Roles for the Histone modifying and exchange complex NuA4 in cell cycle progression in *Drosophila melanogaster*. *Genetics* 203(3): 1265-1281.

Organista, M. F., Marnn, M., de Celis, J. M., Barrio, R., López-Varea, A., Esteban, N., Casado, M. and de Celis, J. F., 2015. The Spalt transcription factors generate the transcriptional landscape of the *Drosophila melanogaster* wing pouch central region. *PLOS Genetics* 11(8), e1005370.

Ostalé, CM., del Prado, A., Martín, M., Esteban, N., López-Varea, A. and de Celis, J.F. (2024). A function of spalt major as a sequence-specific DNA binding transcription factor mediates repression of *knirps* in the *Drosophila* wing imaginal disc. *Developmental Biology* 510: 40- 49.

van Bergeijk, P., Heimiller, J., Uyetake, L. and Su, T. T., 2012. Genome-wide expression analysis identifies a modulator of ionizing radiation-induced p53-independent apoptosis in *Drosophila melanogaster*. *PLoS One* 7, e36539.
