# Supplementary material for: A function of Spalt proteins in heterochromatin organization and maintenance of genomic DNA integrity
Source: Development. 2025 May 16;152(10):dev204258. doi: 10.1242/dev.204258 (PMC12091872; doi:10.1242/dev.204258)
Supplement: Supplementary file 1 [file develop-152-204258_review_history.pdf]

A function of Spalt proteins in heterochromatin organization and maintenance of genomic DNA integrity

Cristina M. Ostalé, Natalia Azpiazu, Ana Peropadre, Mercedes Martín, Mireya Ruiz-Losada, Ana López-Varea, Rebecca R. Viales, Charles Girardot, Eileen EM Furlong and Jos F. de Celis
DOI: 10.1242/dev.204258

Editor: Haruhiko Koseki

Review timeline

Original submission:	18 July 2024
Editorial decision:	15 August 2024
First revision received:	3 January 2025
Editorial decision:	14 February 2025
Second revision received:	5 March 2025
Editorial decision:	31 March 2025
Third revision received:	7 April 2025
Accepted:	15 April 2025

Original submission

First decision letter

MS ID#: dev.204258

MS TITLE: A function of Spalt proteins in heterochromatin organization and maintenance of genomic DNA integrit

AUTHORS: Cristina M. Ostalé, Natalia Azpiazu, Ana Peropadre, Mercedes Martín, Mireya Ruiz-Losada, Ana López-Varea, Rebecca R. Viales, Charles Girardot, Eileen EM Furlong and Jos F. de Celis

Dear Dr de Celis,

I have now received all the referees' reports on the above manuscript, and have reached a decision. The referees' comments are appended below, or you can access them online: please go to:

As you will see, the referees express considerable interest in your work, but have some significant criticisms and recommend a substantial revision of your manuscript before we can consider publication. If you are able to revise the manuscript along the lines suggested, which may involve further experiments, I will be happy receive a revised version of the manuscript. Your revised paper will be re-reviewed by one or more of the original referees, and acceptance of your manuscript will depend on your addressing satisfactorily the reviewers' major concerns. Please also note that Development will normally permit only one round of major revision. If it would be helpful, you are welcome to contact us to discuss your revision in greater detail. Please send us a point-by-point response indicating your plans for addressing the referees' comments, and we will look over this and provide further guidance.

Reviewer 1

© 2025. Published by The Company of Biologists under the terms of the Creative Commons Attribution License (https://creativecommons.org/licenses/by/4.0/).

Advance summary and potential significance to field

In this manuscript, Ostalé et al. assess the impact of a loss of function of Spalt transcription factors on different aspects of Drosophila development. This includes changes in chromatin accessibility and active chromatin marks (H3K27ac) as well as an upregulation of DNA damage genes in developing wings, activation of normally silenced pericentromeric chromatin in eyes and during embryogenesis, mitotic defects and changes to chromatin marks in early embryonic development, and an aberrant nuclear morphology in salivary glands. There is also a comparison of previously published datasets in an effort to relate the observed developmental defects to the DNA damage machinery. While the described developmental defects are certainly interesting and appear correlated, the authors fail, in my view, to explain them mechanistichally or to draw clear connections between them. Hence, it remains unclear how exactly Spalt transcription factors contribute to the described phenotypes, and whether they are a direct or a secondary effect due to the mis-regulation of target genes.

Comments for the author

I would suggest substantial reanalysis of some of the data and inclusion of several discussion points. To be clear, I am not suggesting any new experiments.

- I can't see the supplemental tables, so am unable to review them or aspects of the manuscript relating direclty to them.

- ATAC-seq and H3K27ac data analysis and interpretation:
The bioinformatic methods are not described in enough detail to allow interpretation of the data. For example, what were the parameters for alignment and peak calling? Were peaks called on individual replicates or on both replicates together?
The authors state that they "detected considerable chromatin regions with accessibility changes" (page 7), but it is unclear what this actually means. This should be expanded. What percentage of loci do change accessibility (i.e., how many regions are accessible in control discs and what is the fraction that changes)? Are these changes mainly qualitative or quantitative (i.e., do regions become completely inaccessible/newly accessible or is there mainly a reduction/gain in the level of accessibility of loci that are normally accessible, which would indicate an "accessible in fewer/more cells" phenotype)? It would be helpful to show examples of loci to illustrate these changes (also in correlation to changes of H3K27 acetylation and actual Spalt binding).
In figure 1D, the numbers for the "ATAC down" analysis don't add up. The total number should be 2416, but 1709+32+146=1887
It would also be interesting to test how well native binding of Spalt-m and Spalt-r (data from the same group, Ostalé et al., 2023) is correlated with loci actually changing levels of accessibility or H3K27ac. The fact that this is not mentioned (as far as I can see) makes me think that there is no strong correlation, which would indicate that these changes are due to a secondary mechanism (e.g., involving Spalt target genes).
The authors state that they "found a large number of enriched sequences located more than 5 Kb from the nearest transcription start site" (page 8). In fact, more than half of the sequences for most conditions are propoter-proximal. To really judge these values, it would be necessary to know how much of the genome is promoter-distal vs. proximal. I assume that the fraction of proximal is much lower than distal, given that the Drosophila genome is gene-dense and often the promoter-proximal regions of different genes will occupy the same sequence space, so I wonder if there is really an enrichment of regions changing levels of accessiblity or H3K27ac in promoter-distal sequences. This would of course require statistical testing as well - I am not an absolute expert here, but I think a chi-squared test is appropriate.
I was a bit confused by the fact that the authors first state the pericentromeric chromatin is 30 %, then 14.1 % (page 8). If they only analyse the latter, I would remove the statement of 30 %.
The authors state that they "found a particular enrichment in pericentromeric areas within the regions that gain accessibility in salm/salr knockdown discs (22.1% of total bp whose accessibility is increased [...]" (p.8/9). I don't understand where the "total bp" come from, and it is not clear in the methods how this value was calculated. Is this the range of peaks that gain accessibility? And then always the larger value (e.g., a region gains accessibility and is then called as a larger peak)? Or did the authors generate a set of all peaks (e.g., using bedtools), then tested which ones gain

© 2025. Published by The Company of Biologists under the terms of the Creative Commons Attribution License (https://creativecommons.org/licenses/by/4.0/).

accessibility, then tested what fraction of the total bp (why not fraction of peaks?) belong to peaks that change accessibility?
The reference to Fig. 2F at the beginning of page 8 should be Fig. 1F.
The authors state that they "found a consistent overlap between HP1-bound fragments and all regions whose open state or H3K27ac abundance is modified after salm and salr RNAi expression" (page 9). Again, it would be great if an example locus could be shown here. They go on to say that "the proportion of nucleotides sequenced in each set that is also sequenced after HP1 chromatin immunoprecipitation is significantly higher than the 11.8 % of bp bound by HP1 in the genome (values of 15 % to 22 %". Again, I don't understand where these values come from. Is this the overlap between peaks of each dataset? Would it not be more meaningful to say what percentage of peaks overlaps? Also, the graphs and the statement that the proportion is significantly higher should include some statistical testing (e.g., chi-squared).
I am not entirely sure what the message of the first paragraph on page 10 is. I didn't quite understand where the "genes most likely affected by these epigenetic modifications" come from - again, the methods are a bit thin here. Maybe this can be explained in the main text in one or two sentences. Athough, I think that the next section (comparison to genes that actually change expression after salm and salr RNAi) is much more meaningful.
The comparison to the 1622 genes that change expression needs the in-text citation of the paper where these were analysed. Could the lack of significant overlap between these genes and the changes in chromatin accessibility and H3K27ac levels be explained by the different times of RNAi knockdown in both studies? For RNAseq, salm and salr were only knocked down in the 24 or 48 hours before analysis, while in this study they were knocked down throughout development, and there may be early effects of this knockdown that influence later accessibility/H3K27ac. If this possibility can be excluded, it needs to be discussed. The authors also state that "Genes associated to regions of higher chromatin accessibility in salm/salr knock-down discs generally correspond to genes that are not expressed in wild-type wing discs". Where do these genes come from? How is "not expressed" defined?

- maintenance of heterochromatin
The eye colour assay (page 11) should be explained in more detail. I know many Drosophilists who would not know how this works, let alone people who don't work with Drosophila. I presume that Df(2L)32FP-5/CyO flies are completely white-eyed? A picture showing this would be helpful.
For the qPCR of retrotransposon expression, is it possible that the presence of CyO in the Df(2L)32FP-5 flies is the cause for higher expression? The ideal control would have been yw flies crossed to CyO, but maybe this has been done in another study that can be cited? To be clear, I am not suggesting novel experiments, only that the authors discuss this.
Regarding the localisation of HP1 in the nuclei of salm/salr knockdown salivary glands, the nuclei in Figure 2F have in general a different morphology, which could influence the observation of HP1 localisation. This morphology is described in detail later, but here the nuclei look in fact different from Figure 4. Is this just due to a different staining?

- DNA damage
The authors compare their salm/salr knockdown cells to cells treated with H_2O_2. As far as I know, the latter induces apoptosis. The authors mention in the discussion that there is low level of apoptosis in salm/salr mutant discs, but the paper they cite (Organista & De Celis, 2013) shows a substantial increase in activation of Caspase 3 - the title of the paper even mentions that Spalt transcription factors regulate cell survival. So I am wondering if the DNA damage they detect here comes in fact from dying cells? This should be discussed in more detail.

- nuclear morphology
The authors state that they "focused our analysis in the salivary glands of salm/salr hippomorphic conditions (weak knockdown)" (page 15). This should read "hypomorphic ". It also needs an explanation how this weak knockdown was achieved.
They go on to say that "Salm/salr salivary glands have smaller cells". However, they only show images of nuclei. Have they measured cell sizes?
An inlcusion of scale bars would be helpful. I presume that images in B-E are the same scale but D and E are only half the size (presumably due to smaller cell/tissue size).
Has the difference in nuclear size been quantified in any way? While the cells certainly appear smaller in the knockdown condition, it also appears that Figure 4I shows fewer planes as a z stack

© 2025. Published by The Company of Biologists under the terms of the Creative Commons Attribution License (https://creativecommons.org/licenses/by/4.0/).

than Figure 4H (the image in I is less blurry). There could be other factors contributing to this blurriness, but I think a quantification of nuclear size would be useful.

- irradiation
The authors state that they "searched for links between the transcriptional changes observed in salm/salr mutant discs" (p. 16), but it should be "RNAi discs".
The plots in Fig. 5A would work better as pie charts and venn diagrams as in Fig. 1.
I am not entirely sure about the logFC values given by the authors - could this please be double checked? The paper they are referring to for the irradiation-induced gene expression changes (van Bergeijk et al., 2012) seems to give fold changes, not logFC. So "genes overexpressed after irradiation with logFC in the range of 1 to 6" (page 16) would actually be a 0 to 0.8 logFC if it is "genes overexpressed after irradiation with a fold change in the range of 1 to 6". Otherwise, a logFC of 6 would mean a 1,000,000 fold change in expression, which seems very high. In general, I am more accustomed to changes in expression being given as log(2)FC, but this may be dependent on the data. In any case, if it is indeed logFC, this also needs to be changed in Figure 5B.
The authors need to cite the publication where they mention the "two cases where we identified the enhancer region of the corresponding gene that became active upon a loss of salm/salr function" (page 16).
They also state that "the same enhancers also became activated after irradiation". Has this been done as a part of this study? I don't think it is mentioned in the methods.
Finally, they state that "The increase in expression levels observed after irradiation depends on the transcriptional regulator Dp53". I am not sure what they mean here. Is this an experiment they did? Is this activation of Dp35 independent from Spalt function? How is it related to Spalt?
Citations are missing from the Figure 5 legend. "salm/salr mutant discs showing ectopic expression in the central domain of the wing disc" (Organista et al., 2015) and citation for the images of upregulated genes. Is this form of republication of figures that have already been published (even if only in a supplementary figure) allowed?

In the discussion (page 19), the authors state that "changes in chromatin accessibility are poorly correlated to the changes in gene expression detected in salm/salr mutant wing discs." How can those two seemingly independent effects of Spalt be reconciled? What is the relationship to native Spalt binding? Can this be an effect of the different experimental setup (very precisely timed RNAi in the expresion analysis)? Or does it come down to secondary effects of a gene regulated by Spalt? Are any of the mis-regulated genes known to be involved in the regulation of chromatin accessibility?

In Materials & Methods/Gene expression analyses (page 23), the number of genes with increased expression must be 922, not 822 (265+60+597). Again, the logFC values are a bit odd. 0.22 equates to 1.66-fold change, which seems very arbitrary. Why was this chosen as a cutoff? Also, a logFC of 5.26 equates to a 182,000-fold change in expression, which seems exceptionally high. Can this please be double checked?

In Materials & Methods/Transposable elements expression analysis (page 24), the genotypes of the embryos need to be stated.

In Materials & Methods/Quantification of eye pigment (page 24), the authors state that "Three different measurements are performed". I presume they mean extractions?

In Materials & Methods/ATAC-Seq (page 25), the authors need to give more detail about the stage at which imaginal discs were dissected. Also, the genotypes have to be corrected - they are different from the description in the rest of the manuscript.
There seems to be a doubling of the 30 m incubation at 4 C.
Size-selection with AMPure XP beads using 0.5 - 1.8x - does this mean two steps? First 0.5 to remove large fragments, then 1.8 to retain small ones?
A depth of 50000 reads would be very shallow. I presume this is a typo and should be 50,000,000 (or at least 5,000,000)?

There is no mention of sequencing depth for H3K27ac ChIP-seq.

Materials & Methods/Comet assay: How many replicates were performed?

© 2025. Published by The Company of Biologists under the terms of the Creative Commons Attribution License (https://creativecommons.org/licenses/by/4.0/).

Materials & Methods/Bioinformatic analyses: As mentioned above, this needs more detail (programs, versions, parameters where non-standard).

Materials & Methods/Statistical analyses: Most plots show no significance values. For some charts chi-squared test may be better (e.g., Fig. 1E, F)?

minor comments/typos:
page 3: "tracheal dorsal trunk formation" - might be better to say "formation of dorsal trunk trachea" (if that is what the authors mean here)
page 4: "The multiplicity of functional requirements [...] underly the association of [...]" The sentence doesn't really make sense and should be rewritten.
page 4: "located 11 Kb from the kni transcription start site (Ostalé et al., 2024) and the C. elegans" would red better as two sentences: "located 11 Kb from the kni transcription start site (Ostalé et al., 2024). Likewise, the C. elegans"
page 5: "cell-type specific gene target promotors" should be "cell-type specific target gene promotors"
page 7: "We found that loss of salm/salr function also has a broad impact on the presence of the H3K27ac mark in imaginal cells. Thus, ChIP-seq ("Chromatin immunoprecipitation followed by sequencing") experiments with anti-H3K27ac antibodies in control and salm/salr knock-down discs reveal a similar number of chromatin regions showing increased or decreased H3K27 acetylation" - These two sentences appear the wrong way round?
page 8: "Analyzing the sets of regions that suffer epigenetic modification" - I would suggest to write "whose accessibility changes" or so.
page 16: "reasonably overlap" should be "reasonable overlap"
page 17: "presumptive longitudinal L2 vein" should be "future longitudinal L2 vein"
page 18: "It was also unknown" should probably be "It is also unknown"?
page 23: AB1-Gal4 and UAS-mCD8-GFP don't have a stock number.
page 28: "Inmunocitochemistry" should be "Immunohistochemistry"
page 28: "A total of 40 wing imaginal discs cells per replicate" should be "A total of 40 wing imaginal discs per replicate"
page 41: "salm/salr mutant wing imaginal discs" should be "salm/salr RNAi/knockdown wing imaginal discs"

<u>Reviewer 2</u>

Advance summary and potential significance to field

In this manuscript, Ostale et al. investigate the dual roles of phylogenetically conserved Spalt proteins in Drosophila development. These proteins are known to regulate gene expression and influence cell fate by acting as transcriptional repressors within gene regulatory networks. Interestingly, Spalt proteins also localize to heterochromatic regions, suggesting a role in epigenetic regulation. In this study, the authors describe the impact of Spalt protein loss in Drosophila cells, noting changes in chromatin accessibility, DNA damage, chromosome localization in polyploid salivary gland cells, and misexpression of transposable elements. They propose that Spalt proteins function both as sequence-specific transcriptional repressors and as regulators of global gene silencing through heterochromatin formation.

In principle, the topic of this study is of general interest, as sequence-specific transcription factors like Spalt are just starting to be recognized as organizers of heterochromatin. However, this manuscript is currently not in a state to be published in Development due to several major and minor issues.

Comments for the author

Major Issues:

1. In the abstract, the authors highlight the interaction of Spalt with specific epigenetic regulators like NuRD or LSD1, which is not the topic of the paper. This should be toned down, as one expects that this interaction is at least partly analyzed in this research.

© 2025. Published by The Company of Biologists under the terms of the Creative Commons Attribution License (https://creativecommons.org/licenses/by/4.0/).

2. The language is sometimes not correct and should be checked.
3. Has the interaction of Drosophila Spalt with heterochromatin been shown before, as on page 5 only vertebrate Spalt is mentioned? The same is true for the interaction with heterochromatic proteins.
4. A major claim of the paper is that there is only a minor overlap of changes in chromatin accessibility and changes in gene expression in wing discs after spalt knock down. Where is this data shown? It would be important to backup this statement with real data. Is this the case for both activated and repressed genes by Spalt?
5. How many of the observed changes in chromatin accessibility are a direct consequence of loss of Spalt binding? In other words: How many of the identified ATAC regions in control are bound by Spalt (according to the recent published paper from this lab)? How many of these Spalt bound regions show changes in chromatin accessibility in Spalt knock down conditions in comparison to all the changes in chromatin accessibility observed?
6. One histone mark enriched in pericentromeric heterochromatin regions is H3K27me3, which should be reduced in Spalt knock down conditions. The authors should backup their study with profiling H3K27me3 in spalt knock down wing discs.
7. The author found that chromatin regions that change their accessibility after spalt knockdown are bound by HP1. What is the overlap of Spalt and HP1 binding in wild type wing discs? How much of HP1 binding is lost in Spalt KD conditions? Do Spalt and HP1 interact with each other?
8. The information contained in Fig. 1G - K is unclear.
9. The authors jump between cellular models, from wing disc, to eyes, to embryos, to salivary glands, without stating why these particular systems were chosen for the specific experiment. Why were for example 0-2 h embryos taken to study retrotransposon activation in embryos (Page 12, first paragraph)? In the figure showing the result (Fig. 2F), the x-axis is not labelled and it is only clear from the Materials and Methods that the expression of retrotransposon activation was measured by qPCR.
10. The authors then jump from embryos to salivary glands to look at the nuclear localization of HP1 after spalt knockdown. The authors claim that HP1 localizes here (Fig. 1G, G') to a single spot which corresponds to the chromocenter. There is no co-labelling nor a citation that supports this claim. In Fig. 2H, H', DAPI staining is different compared to the control (2G, G'), as the center of the nucleus is free of DAPI. What does this mean? Changes in distribution of chromatin?
11. The authors then jump again to blastoderm embryos to study mitotic defects in spalt heterozygous mutants. A brief statement why this model now would be very helpful. The authors claim to see the formation of anaphase bridges from DAPI stainings. Additional stainings of the microtubule would be necessary to substantiate this claim. What is the amount of these defects? Quantifications are missing.
12. In all figures, scale bars are missing.
13. The authors then perform DNA damage analysis in wing discs. Why this model for this question? The authors should perform gammaH2Av stainings, which is a marker for DNA damage to directly visualize DNA damage in the wing disc. It is unclear what Fig 3B-C should show. What does DNA in tails and Tail moment (Fig. 4) mean? It is unclear what is shown in this figure.
14. Again, no scale bars in Fig 4, so it is unclear whether there are any changes in the size of the salivary gland. For a non-expert, it is impossible to judge how a salivary gland looks like and what to expect to see.
15. What are "hippomorphic conditions" (page 15)?

Additional comments:

1. Page 5: It would be nice if authors can cite or mention few interactors or a study that probes the interaction partners of sal proteins to give more context splat functionality.
2. Page 7: How efficient is the salm/salr-RNAi lines? Is there already a validation present in previous research work and if not, did the authors validate the extent of RNAi? The choice of RNAi over knockout can be explained for this context as well. Is the knockout of salm/salr embryonic lethal?
3. Page 8: I think authors can mention here that, both distal intragenic and between gene regions have been indicated as distal regions in the figure. Page 8: The x-axis labelling in figure F (referred from this page number) is confusing. The figure legend says the order is ATAC-UP, ATAC-DOWN, H3K27ac-UP, H3k27ac-DOWN, whereas on x-axis in the figure it is H3K27AC-DOWN before H3K27ac-UP.
4. Page 9: This should be Figure 1F and not figure 2F (labelling/reference mistake).

© 2025. Published by The Company of Biologists under the terms of the Creative Commons Attribution License (https://creativecommons.org/licenses/by/4.0/).

5. Page 9: Figure F' and Figure F'' instead of Figure 1F in line 22 and 24
6. Page 10: the figure says H3K29ac, should not it be H3K27ac?
7. Page: 10 Paragraph second: This is very confusing to read, should be rephrased or made simpler.
8. Page 10: Can the authors give a rationale behind choosing significant expression change using p-value over fold change in gene expression? Could it be related to the fact that the knockdown, rnai is inefficient?
9. Page 10 last paragraph/11 first paragraph: The authors should discuss possible limitations that could have affected the approach to this conclusion. Like the the gene association by the window of distal regions and incomplete effects of rnai. That would also strengthen the conclusions which right now seems very broad.
10. Page 14: Tail moment seem similar in salm salr rnai vs wt discs from the plot and seem significantly different in h2o2 discs? Should not the plot look similar to % DNA plot (in context of wt vs mutant vs H2O2 discs)?
11. Page 14 (Figure C G-H'): There should be quantifications for these images. It seems pretty clear but could be misleading because of the background in the images.
12. Page 15: Is the weak knockdown that authors talk about because of the RNAi used specifically in this case?
13. Page 16 (Figure 5C): Was there an in-situ done for gadd45 and ver as well in case of salm/salr rnai? Because that would to more concretely connect the xray irradiation response and sal deficiency response.
14. No scale bars in images: Specifically, required in figure 4 for the context of size of salivary gland. Figure H and I seems to be taken at different magnification.

In summary, the manuscript presents a collection of descriptive findings without pursuing the intriguing results further, leaving significant gaps in the research narrative. The rationale for selecting specific models remains unarticulated, and the figures are assembled with a lack of precision and coherence. The overall impression is one of disparate research elements compiled into a single manuscript, rather than a cohesive and well-integrated study. Consequently, I find myself unable to recommend this manuscript for publication in Development.

Reviewer 3

Advance summary and potential significance to field

In this manuscript, the authors provide evidence that in addition to their function as sequence-specific transcriptional repressors, Spalt proteins also regulate gene expression more globally by generation or maintenance of heterochromatic domains. The data suggest that loss of Spalt function results in changes in chromatin organization. The claim that Spalt proteins are required for maintenance of genomic DNA integrity is strongly supported by the data provided. Loss of spalt function could lead to mitotic errors in mutant diploid cells resulting in a delay of cell cycle progression and to morphological changes in mutant polyploid cells. Interestingly, in addition to heterochromatic regions that become more accessible upon salm/salr knockdown, a similarly high number of genomic regions becomes less accessible suggesting that Spalt proteins also have other functions not discussed in this paper. Thus, the data generated in this paper could provide a base for further research on the role of Spalt proteins not only in flies, but also in mammalian systems.

Comments for the author

Essential revisions

1) Given the multitude of genes and genomic regions affected by the loss of Spalt proteins and the severe changes in nuclear organization observed in cells after salm/salr knockdown, it is surprising that Spalt expression is not required in all cells. How is DNA damage and chromatin mis-organization prevented in the cells of the wing disc that do not express Spalt? Or, in other words, why do the cells in the center of the wing disc require Spalt proteins, while those in the periphery do not?

© 2025. Published by The Company of Biologists under the terms of the Creative Commons Attribution License (https://creativecommons.org/licenses/by/4.0/).

2) On page 15, the authors claim that there is a high variability in salivary gland size between the different genotypes they looked at (loss of salm/salr function in these cells causes a variety of phenotypes, ranging from rudimentary salivary glands to morphologically normal glands, depending on the particular Gal4/UAS-RNAi combination). It would be helpful to show this for example with box plots of salivary gland length (and width) and/or of nuclear diameters for the different genotypes.

3) I could not find a reference for the Gadd45 and ver reporter constructs in the manuscript, nor where they described in the materials and methods section. Were the enhancer regions of Gadd45 and ver identified in this study? Are the Gadd45 RR-GFP and ver RR-GFP GFP reporters mis-expressed in salm/salr knockout cells? Is their expression in irradiated cells dependent on the presence of P53 protein?

Additional minor comments

1) In figure 1 panel F, the order of the conditions (in the x-axis) is switched between A3K27ac DOWN and UP as compared to panel E (and G, J, and K). Is this just an error in the labeling of the axis or is there really a smaller coverage of pericentric heterochromatin in the cases where acetylation goes up?

2) In the pie-charts in figure 1 panels H and I, the absolute numbers are given with the percentages in brackets (some brackets are missing in panel I), while in panels J and K the percentages are given first with the absolute numbers written below in a smaller font (n=). It would be nicer to have those displayed in a more consistent way throughout the entire figure.

3) Figure 2 panels A to E: in the main text (page 11) the authors state that the pictures are showing female eyes, whereas in the figure legend they claim that these are male eyes. In the materials and methods section (page 24), they just say they used 15 heads of each genotype for the quantification of eye pigment without specifying if these were males or females (or both). In addition, in the main text: "…we found a consistent increase in eye pigmentation comparing P{hsp26-p-T} w[67c23] expression…", the w[67c23] is a mutation in the endogenous white gene on the X-chromosome, it is not expressed in these flies. The expression they compare is due to a mini-white marker in the inserted P-elements. In the interest of consistency, it would also be nicer to have the results in panel E in the same order as in panels A to D (first the CyO in black, then the Df5 in grey). This would also be more consistent with panel F where the control (wt) conditions are shown in black and the Df5 conditions are shown in grey.

4) I would assume, that the images in figure 2 panels M and N were taken with the same settings for the different channels, but this is nowhere stated in the text. Are the nuclei bigger in panel N than in panel M? Are they in the same replication phase?

5) In the figure legend for panel A of figure 3 (and 4) the authors write: "The expression of DAPI is shown in blue". DAPI is not a protein that is expressed, but a staining for DNA.

6) In figure 3 panels G' and H' I can see a higher P53 expression in the sal-domain, but the P53 levels are also increased outside of the central area in panel H as compared to G, and the cellular pattern looks different (less punctate). Where the images (especially the red channel) taken with the same settings? In the materials and methods section there is nothing written about these particular stainings or the acquisition of the images.

7) Scalebars are missing in the entire paper. This is particularly irritating in figure 4 panels B to E, which are supposed to display differences in the size of the salivary glands of the different genotypes. Even in the materials and methods section there is nothing written about the acquisition of these images or how they were processed (e.g. cropped).

8) For the TEM images in figure 4 panels F and G, the genotypes are not clear. In the figure legend the authors state that they used wt salivary glands (in panel F) and salEPv-Gal4 to express the RNAi constructs (in panel G), whereas in the materials and methods section (page 27) they mention 3 different genotypes ("Third instar larvae of phm-Gal4 UAS-GFP UAS-GFP (25 °C), phm-Gal4 UAS-GFP Gal80ts UAS-salm-i UAS-salr-i (2 days at 25 °C, 5 days at 17 °C and 5 days at 29 °C)

© 2025. Published by The Company of Biologists under the terms of the Creative Commons Attribution License (https://creativecommons.org/licenses/by/4.0/).

and UAS-salmi/+; AB1-Gal4/UAS-salr-i (25°C) genotype") for this experiment, where none of them contains salEPv-Gal4.

9) In figure 4 panels H and I: The authors state that "the size of the nucleolus is much larger in salm/salr mutants compared to normal glands, and the chromosomes have an abnormal disposition within the nuclei". Does the nucleolus only appear bigger due to the smaller size of the nucleus? The authors should also mention somewhere that Fibrillarin is localizing to the nucleolus.

10) In figure 5 panel A, the first 2 columns are both labeled "SAL UP". So, what is the difference there? Or is one of them supposed to display SAL DOWN?

11) In figure 5 panels H and I, where the discs imaged with the same settings? Panel H would seem to be a better image to compare the even P53 expression pattern with the enhanced expression in the salm/salr knockdown disc in figure 3H.

12) There is no reference to Supplementary figure 1 anywhere in the manuscript. The numbers in suppl. Table 1 do not exactly correspond to the numbers in figure 1 panels A and B. For example: there are 910 hits with an FDR below 0.001 listed in the ATAC_UP tab of the table. But only 899 hits given in the plot in panel A and only 73 of them are also UP in H3K27ac while only 5 are also DOWN in contrast to the 111 UP and 11 DOWN overlaps in panel C. Please explain.

13) On page 15 the authors wrote: "We focused our analysis in the salivary glands of salm/salr hippomorphic conditions (weak knockdown)". The correct word would be hypomorphic. I found some more spelling and grammatical errors, but this is the most relevant one, because it changes the meaning of the word.

First revision

<u>Author response to reviewers' comments</u>

Reviewer 1
SUMMARY OF THE ADVANCE MADE IN THIS PAPER AND ITS POTENTIAL SIGNIFICANCE TO THE FIELD

In this manuscript, Ostalé et al. assess the impact of a loss of function of Spalt transcription factors on different aspects of Drosophila development. This includes changes in chromatin accessibility and active chromatin marks (H3K27ac) as well as an upregulation of DNA damage genes in developing wings, activation of normally silenced pericentromeric chromatin in eyes and during embryogenesis, mitotic defects and changes to chromatin marks in early embryonic development, and an aberrant nuclear morphology in salivary glands. There is also a comparison of previously published datasets in an effort to relate the observed developmental defects to the DNA damage machinery. While the described developmental defects are certainly interesting and appear correlated, the authors fail, in my view, to explain them mechanistically or to draw clear connections between them. Hence, it remains unclear how exactly Spalt transcription factors contribute to the described phenotypes, and whether they are a direct or a secondary effect due to the mis-regulation of target genes.

We fully understand the reviewer criticism concerning the lack of a detailed mechanistical explanation accounting for all our observations. We consider that at this point our manuscript mostly provides a plausible scenario where to frame the developmental and cellular consequences of loss of Spalt function, which we think is a necessary step to test mechanistic explanations. Although we cannot entirely answer this key criticism, we have included in Figure 6 a cartoon addressing the connection between our observations and what we consider the most likely affected biochemical mechanisms. Of course, at this point this proposal should be considered as a working hypothesis, but at least is a reasonable framework to connect the alterations observed in *spalt* mutants under a molecular mechanism.

© 2025. Published by The Company of Biologists under the terms of the Creative Commons Attribution License (https://creativecommons.org/licenses/by/4.0/).

SUGGESTIONS TO AUTHORS

I would suggest substantial reanalysis of some of the data and inclusion of several discussion points. To be clear, I am not suggesting any new experiments.

We have reworked our analysis of the curated data and changed the narrative of the corresponding sections of the manuscripts. We have included a new Figures 1 and 2, and made considerable changes to the section "Loss of *spalt* function has a profound impact on chromatin accessibility" (pages 7-11), which has been rewritten to improve accuracy, order and clarity.

1) I can't see the supplemental tables, so am unable to review them or aspects of the manuscript relating directly to them.

We apologize to the reviewer for this mistake, and will make sure that the supplementary tables are included during resubmission.

2) ATAC-seq and H3K27ac data analysis and interpretation:The bioinformatic methods are not described in enough detail to allow interpretation of the data. For example, what were the parameters for alignment and peak calling? Were peaks called on individual replicates or on both replicates together?

The authors state that they "detected considerable chromatin regions with accessibility changes" (page 7), but it is unclear what this actually means. This should be expanded. What percentage of loci do change accessibility (i.e., how many regions are accessible in control discs and what is the fraction that changes)? Are these changes mainly qualitative or quantitative (i.e., do regions become completely inaccessible/newly accessible or is there mainly a reduction/gain in the level of accessibility of loci that are normally accessible, which would indicate an "accessible in fewer/more cells" phenotype)? It would be helpful to show examples of loci to illustrate these changes (also in correlation to changes of H3K27 acetylation and actual Spalt binding).

In figure 1D, the numbers for the "ATAC down" analysis don't add up. The total number should be 2416, but 1709+32+146=188.

We have included now a detailed description of the bioinformatic methods that should answer the reviewer criticism. Peaks were individually called, as required by the DiffBind package.

<u>Page 34-35; Added</u>:

"The analysis of H3K27ac and ATAC analysis was performed using the Galaxy platform. Paired-end reads were cleaned for Nextera adaptors using *Trimmomatic* (Bolger et al., 2014; Galaxy Version 0.36.6, with options ILLUMINACLIP: NexteraPE-PE.fa:2:30:10:8:true, SLIDINGWINDOW:4:20, MINLEN:20, SLIDINGWINDOW:4:20) for ATAC-seq and using Trim Galore! (Galaxy Version 0.4.3.1 with automatic adaptor detection, Trim low-quality ends from threshold: 20, Overlap with adapter sequence required to trim a sequence: 2, Maximum allowed error rate: 0.1, reads becoming shorter than 20 were discarded) for ChIP-seq (retrieved from: https://www.bioinformatics.babraham.ac.uk/projects/trim_galore/.
The remaining of the workflow is common to ATAC-seq and ChIP-seq. Trimmed reads were mapped to the *Drosophila* reference genome dm6 version (The Berkeley *Drosophila* Genome Project BDGP) with Bowtie2 (Langmead and Salzberg, 2012; Galaxy Version 2.3.4.3+galaxy0, with options –fr -I 0 -X 2000 –sensitive –dovetail). Mapped reads were then filtered using *Filter SAM or BAM, output SAM or BAM files on FLAG MAPQ RG LN or by region* (Li et al., 2009; *Galaxy Version 1.1.2)* to only keep read mapped in a proper pair with MAPQ > 19 (which eliminates multi-mapping reads). We also only kept read pairs that mapped to major chromosomes (chr2L, chr2R, chr3L, chr3R, chr4 and chrX) and removed duplicate reads using *Picard MarkDuplicates (Galaxy Version 2.7.1.1; retrieved from http://broadinstitute.github.io/picard/).* "25bp 1X-normalized" BigWig Signal Files were created using *DeepTools bamCoverage (Ramirez et al., 2016; Galaxy Version 3.0.2.0)* at a 25bp resolution with the normalized to 1x coverage option. Quality was assessed using different tools. *FastQC (Galaxy Version 0.69, retrieved from http://www.bioinformatics.babraham.ac.uk/projects/fastqc/)* was run at different steps of the workflow to check sequencing quality and monitor filtering step efficiency. *Picard Collect*

© 2025. Published by The Company of Biologists under the terms of the Creative Commons Attribution License (https://creativecommons.org/licenses/by/4.0/).

Alignment Summary Metrics (Galaxy Version 2.7.1.1; retrieved from http://broadinstitute.github.io/picard/) was used to evaluate library quality across samples (read duplication and unmapped reads rates) and *Picard CollectInsertSizeMetrics (Galaxy Version 2.7.1.0; retrieved from http://broadinstitute.github.io/picard/)* to compare fragment length. Finally, *Deeptools plotFingerprint (Ramirez et al., 2016; Galaxy Version 3.0.2.0)* was used to check ChIP signal strength. The DiffBind R package (Stark R, Brown G (2011). DiffBind: differential binding analysis of ChIP-Seq peak data. http://bioconductor.org/packages/release/bioc/vignettes/DiffBind/inst/doc/DiffBind.pdf) was used to perform differential binding analysis between controls and *salm/salr* knockdowns using ChIP-seq peaks called with *MACS2 callpeak* (Zhang et al., 2008, Galaxy Version 2.1.1.20160309.5, with options: -call-summits, q-value 0.1. and default paired-end options) performed on the filtered IP BAM files (uniquely mapped reads) and their matched input BAM as control. Sequence comparisons between samples and replicates was carried out using the Galaxy platform (The Galaxy Community et al., 2022). We used the databases OregAnno (Lesurf et al., 2016), RefFlat (Universidad de California, Santa Cruz, http://genome.ucsc.edu/goldenPath), and the following ModENCODE datasets: ChIP-Seq H3K9me3 (Oregon L3, Karpen, G. Dataset ID: 4952) and ChIP-Seq HP1 (Oregon L3, Karpen, G. Dataset ID: 4936).”

We measured changes in a *salm/salr* knockdown background compared to a wild type control, and therefore the changes we identify are quantitative. Furthermore, because the driver we used to direct RNAi is expressed only in a subset of cells, we expect that most cells in the control and experimental populations are genetically identical. We cannot distinguish between similar changers in the level of accessibility in all cells or some cells with very large changes. Our assumption is that all affected cells (those in the central part of the wing blade) change in a similar manner. We have included a Supplementary Figure 3 showing graphical examples of the 8 genes that were associated to Salm binding and with changes in both accessibility and H3K27ac.

The correlation between actual binding of Salm (the only one for which we have data) and changes in ATAC or H3K27ac is very low. We have now included such data in the Supplementary Table 1 and in a new Supplementary Figure 3. We agree that many changes could due to a secondary mechanism, perhaps involving Sal direct target genes or perhaps involving indirect chromosomal effects of Sal loss. We have added the following paragraph to clarify and point out this fact:

Page 10, added: “However, a large fraction of changes in ATACseq and H3K27Ac are not correlated with the changes in gene expression observed in *salm/salr* knockdown wing imaginal discs, suggesting that these changes could be caused by indirect mechanisms triggered by *salm/salr* loss.”

4) *The authors state that they "found a large number of enriched sequences located more than 5 Kb from the nearest transcription start site" (page 8). In fact, more than half of the sequences for most conditions are propoter-proximal. To really judge these values, it would be necessary to know how much of the genome is promoter-distal vs. proximal. I assume that the fraction of proximal is much lower than distal, given that the Drosophila genome is gene-dense and often the promoter-proximal regions of different genes will occupy the same sequence space, so I wonder if there is really an enrichment of regions changing levels of accessibility or H3K27ac in promoter-distal sequences. This would of course require statistical testing as well - I am not an absolute expert here, but I think a chi-squared test is appropriate.*

We have included the values of distances to TSS for all datasets (ATAC-UP, ATAC-DOWN, H3K27ac-UP and H3K27ac-DOWN) in the new Fig. 1 (Fig. 1B, G). Comparing these values to each other, as we do not have the value for the genome, we can see clear differences in the enrichment of distal sequences (ATAC-UP; Fig. 1C) and proximal sequences (H3K27ac-DOWN; Fig. 1G).

5) *I was a bit confused by the fact that the authors first state the pericentromeric chromatin is 30 %, then 14.1 % (page 8). If they only analyse the latter, I would remove the statement of 30 %.*

It was a mistake. Now the data is corrected to 14% and explained in more detail in material and methods:

Pages 30-31, added:

© 2025. Published by The Company of Biologists under the terms of the Creative Commons Attribution License (https://creativecommons.org/licenses/by/4.0/).

“Determination of pericentromeric regions

The extent of pericentromeric regions within the entire *Drosophila* genome was based on the epigenetic boundaries defined by Riddle et al. (2011) between euchromatin and heterochromatin, according to the locations of abrupt transitions in H3K9 dimethylation (H3K9me2) on the X chromosome and autosomes 1, 2, and 3. We considered the entire 4th chromosome as formed by pericentromeric chromatin throughout its length. For the 3R chromosome arm, which lacks an H3K9me2 epigenetic barrier, we used the cytological barrier established by Hoskins et al. (2007). Given that the annotated length for these heterochromatin sequences (annotation release 6, dm6) is approximately 19 million bases (19Mb), and the total genomic length with annotated sequences is approximately 134Mb (133,880,608 bp, excluding the Y chromosome since we did not distinguish by sex in the experimental design), we determined that 14.1% of the total genome length corresponds to pericentromeric chromatin.”

6) The authors state that they "found a particular enrichment in pericentromeric areas within the regions that gain accessibility in salm/salr knockdown discs (22.1% of total bp whose accessibility is increased [...]" (p.8/9). I don't understand where the "total bp" come from, and it is not clear in the methods how this value was calculated. Is this the range of peaks that gain accessibility? And then always the larger value (e.g., a region gains accessibility and is then called as a larger peak)? Or did the authors generate a set of all peaks (e.g., using bedtools), then tested which ones gain accessibility, then tested what fraction of the total bp (why not fraction of peaks?) belong to peaks that change accessibility?

We have now made both calculations, the fraction of bp with overlap to pericentromeric heterochromatin sequences and the fraction of peaks included within pericentromeric heterochromatin sequences (new Fig. 2 and Supple. Table 3). Using the ATAC-Seq data, we defined the sequences in an open chromatin state under wild-type conditions and in *spalt*-knockdown conditions. We utilized the Galaxy platform and the DiffBind (DeSeq) tool to compare these conditions, identifying peaks that significantly gained (ATAC_UP) or lost (ATAC_DOWN) conformational openness in spalt-deficient discs compared to wild-type discs. We also examined which sequences within our *sal*-modified peak sets fell within pericentromeric chromatin regions and calculated the percentage of the total length corresponding to pericentromeric chromatin within each sequence set. These analyses are now presented in the new Figure 2, and the numeric values used to make these calculations are included in the new Supplementary Table 3. We also include the overlap in bp to provide a more informative context for the reader, because it allows for a more direct comparison with the proportion of pericentromeric chromatin in the whole genome.

To clarify this issue, we have added the following paragraphs to material and methods:

<u>Page 31, inserted</u>:

“Calculation of the overlap between the ATAC and H3K27ac datasets with pericentromeric chromatin and with HP1a and H3K9me3 ChIP datasets

The coordinates for the sequences bound to HP1 or modified with H3K9me3 were obtained from chromatin immunoprecipitation experiments conducted by Karpen, G. (modENCODE ID 4936 and 4952, respectively). We used the total sequences obtained in these experiments to establish the percentage of the total sequenced genome that is bound to HP1 (11.8%) or modified with H3K9me3 (14.5%), the sequences and peaks included within pericentromeric heterochromatin (Supple, Table 3) and the overlap between the ATAC and H3K27ac datasets with the HP1 and H3K9me3 datasets (Supple. Table 3).”

7) The reference to Fig. 2F at the beginning of page 8 should be Fig. 1F.
The authors state that they "found a consistent overlap between HP1-bound fragments and all regions whose open state or H3K27ac abundance is modified after salm and salr RNAi expression" (page 9). Again, it would be great if an example locus could be shown here.

We have included the data of HP1 and H3K9me3 binding in Supplementary Table 3 and Figure 2.

© 2025. Published by The Company of Biologists under the terms of the Creative Commons Attribution License (https://creativecommons.org/licenses/by/4.0/).

8) They go on to say that "the proportion of nucleotides sequenced in each set that is also sequenced after HP1 chromatin immunoprecipitation is significantly higher than the 11.8 % of bp bound by HP1 in the genome (values of 15 % to 22 %". Again, I don't understand where these values come from. Is this the overlap between peaks of each dataset? Would it not be more meaningful to say what percentage of peaks overlaps? Also, the graphs and the statement that the proportion is significantly higher should include some statistical testing (e.g., chi-squared).

The coordinates for the sequences bound to HP1 were obtained from the chromatin immunoprecipitation experiment conducted by Karpen, G. (modENCODE ID 4936). This allowed us to determine the total length sequenced in this experiment, enabling us to establish the percentage of the total sequenced genome (approximately 134Mb, excluding the Y chromosome) that is bound to HP1, which is 11.8%. We analyzed the overlap between the sequences bound to HP1 and those in our sets of sequences that undergo modification due to spalt deficiency. We find more informative to provide the percentage of overlap in base pairs rather than in the number of peaks, allowing direct comparison to percentage of coverage to the whole genome. Additionally, this decision is also based on the fact that peaks vary significantly in size, with some being very large, and not always overlapping completely in their entire length. By presenting the data as the percentage of total base pairs, we aimed to offer a clearer and more meaningful comparison for the reader. These data are now in Figure 2 and Supple. Table 3

9) I am not entirely sure what the message of the first paragraph on page 10 is. I didn't quite understand where the "genes most likely affected by these epigenetic modifications" come from - again, the methods are a bit thin here. Maybe this can be explained in the main text in one or two sentences. Athough, I think that the next section (comparison to genes that actually change expression after salm and salr RNAi) is much more meaningful.

We have rewritten all the section related to ATAC and H3K27ac experiments

10) The comparison to the 1622 genes that change expression needs the in-text citation of the paper where these were analysed. Could the lack of significant overlap between these genes and the changes in chromatin accessibility and H3K27ac levels be explained by the different times of RNAi knockdown in both studies? For RNAseq, salm and salr were only knocked down in the 24 or 48 hours before analysis, while in this study they were knocked down throughout development, and there may be early effects of this knockdown that influence later accessibility/H3K27ac. If this possibility can be excluded, it needs to be discussed. The authors also state that "Genes associated to regions of higher chromatin accessibility in salm/salr knock-down discs generally correspond to genes that are not expressed in wild-type wing discs". Where do these genes come from? How is "not expressed" defined?

We have rephrased this section according to the new analysis, in which we only included genes whose expression changes not only significantly, but also by a greater magnitude (logFC of +/- 0.5). The genes that we defined as expressed or not expressed in the wing disc come from RNAseq data previously published (Flegel et al. 2016). We have revised that the references to this work is now included.

<u>Page 24, added</u>: “Finally, to determine gene expression in wing type wing discs, we used RNA-Seq data from wing imaginal discs expressing Gal4/GFP (run SRR3478156; Flegel et al. 2016) quantified using Sailfish 0.7.6.0 running at the Galaxy platform.”

The reviewer is correct in that the experiments we are comparing are not identical, but differ in the knockdown regime. However, the RNAseq data we have at 24 and 48 h after *salm/salr* knockdown are very consistent, and this is the best comparison we can make at this stage. Similarly, the changes in expression as a result of irradiation that we used for comparisons correspond to a very different experimental setup, but still can be compared with the *salm/salr* knockdown RNAseq. Of course, we cannot exclude the possibility of late changes in accessibility or H3K27ac modifications caused by earlier knockdowns in these experiments as a contributor to the discrepancies between these two datasets. We have now included this caveat in the manuscript.

© 2025. Published by The Company of Biologists under the terms of the Creative Commons Attribution License (https://creativecommons.org/licenses/by/4.0/).

Page 24, added: "All these datasets (*salm/salr* knockdown RNAseq, Xray RNAseq and the *salm/salr* knockdowns ATAC and H3K29ac ChIP) were compared with each other looking for similarities, even though they were extracted in different experimental settings."

11) The eye colour assay (page 11) should be explained in more detail. I know many Drosophilists who would not know how this works, let alone people who don't work with Drosophila. I presume that Df(2L)32FP-5/CyO flies are completely white-eyed? A picture showing this would be helpful.

We have modified the corresponding section in Material and methods to explain the bases of this assay:

Page 24-25, modified and added:

"Eye color assays and quantification of eye pigment

To analyze gene expression in heterochromatic regions we monitored the expression of the gene *white* (*w*) present in *P(w+)* subtelomeric insertions located in the 2R and 3R chromosomes. *w-; Df(2L)32FP-5/CyO* flies are white-eyed. When crossed with *w; P(w+)* flies, the red eye color in the *w; Df(2L)32FP-5/P(w+)* or *w; CyO/P(w+)* progeny comes exclusively from the *white* mini gene expressed from the P(w+) insertion. This means that the intensity of red color observed in each genotype depends on the levels of *white* expressed from the P element. We measured pigments from 15 female heads of each genotype. The heads were frozen at -20 for 5 days and then homogenized in methanol acidified with 0,1M HCl (1:9) to extract the pigment. The extracted pigment was measured at 480nm. Three independent extractions were performed for each genotype.

12) For the qPCR of retrotransposon expression, is it possible that the presence of CyO in the Df(2L)32FP-5 flies is the cause for higher expression? The ideal control would have been yw flies crossed to CyO, but maybe this has been done in another study that can be cited? To be clear, I am not suggesting novel experiments, only that the authors discuss this.

We are not aware of any report suggesting effects of Balancer chromosomes on the levels of expression of retrotransposons. We have included a sentence to discuss this possibility, which we have not considered as likely.

Page 26, modified:

"RNA from 0-2 hours old embryos from Df(2L)32FP5)/CyO parents was extracted using the GE Healthcare extraction kit. We used wt embryos as controls, as there is no likely effect of balancer chromosomes (CyO) on the expression of retrotransposons (Ghavi-Helm et al., 2020)."

13) Regarding the localisation of HP1 in the nuclei of salm/salr knockdown salivary glands, the nuclei in Figure 2F have in general a different morphology, which could influence the observation of HP1 localisation. This morphology is described in detail later, but here the nuclei look in fact different from Figure 4. Is this just due to a different staining?

The nuclei in Figure 3H should be compared with those shown in Figure 5C and 5O, and at the resolution offered by confocal pictures they look similar to us, with enlarged nucleoli and DNA displaced towards the periphery of the nuclear envelope:

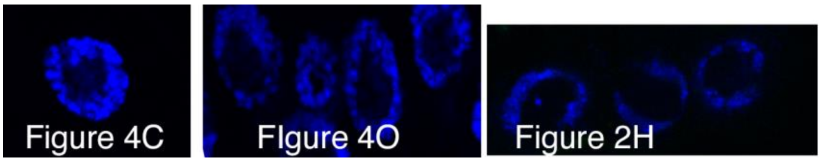

14) *The authors compare their salm/salr knockdown cells to cells treated with H2O2. As far as I know, the latter induces apoptosis. The authors mention in the discussion that there is low level of apoptosis in salm/salr mutant discs, but the paper they cite (Organista & De Celis, 2013) shows*

© 2025. Published by The Company of Biologists under the terms of the Creative Commons Attribution License (https://creativecommons.org/licenses/by/4.0/).

a substantial increase in activation of Caspase 3 - the title of the paper even mentions that Spalt transcription factors regulate cell survival. So I am wondering if the DNA damage they detect here comes in fact from dying cells? This should be discussed in more detail.

The reviewer is correct, and in the Organista and de Celis (2013) paper we describe a modest increase of Cas3 activation in *salm/salr* mutant discs. It has been proposed that cell death could result in comets with over 90% tail DNA, often referred to as 'hedgehogs', (Olive et al., 1993). We have added the following paragraph to the material and methods section to discuss the possible contribution of dying cells to the DNA comet results and to better clarify the use of our positive control. We point out that the exposure to H_2O_2 is made by treating gel-embedded cells (not the wing disc) during 10 minutes.

<u>Pages 33, added</u>:

"We compared control discs (*sal*^{EPv}*-Gal4/UAS-GFP*) with *sal* mutant discs (*sal*^{EPv}*-Gal4/UAS-salm-RNAi; UAS-salr-RNAi/+*) and used as positive controls slides of gel-embedded *sal*^{EPv}*-Gal4/UAS-GFP* wing disc nuclei submerged into H_2O_2 50 □M for 10 min. In these conditions, H_2O_2 induces strand breaks to create DNA comet tails of suitable size. Because loss of *sal* function in the wing blade causes a low level of apoptosis (Organista and de Celis, 2013), we conducted preliminary trypan blue assays to confirm that the proportion of positive cells did not exceed 10%. Additionally, comets showing "hedgehogs", "clouds" or "ghosts", which are generally associated to dying cells (Olive et al., 1993), were routinely excluded during scoring to prevent biases related to cell death."

15) *The authors state that they "focused our analysis in the salivary glands of salm/salr hippomorphic conditions (weak knockdown)" (page 15). This should read "hypomorphic ". It also needs an explanation how this weak knockdown was achieved.*

They go on to say that "Salm/salr salivary glands have smaller cells". However, they only show images of nuclei. Have they measured cell sizes? An inclusion of scale bars would be helpful. I presume that images in B-E are the same scale but D and E are only half the size (presumably due to smaller cell/tissue size).
Has the difference in nuclear size been quantified in any way? While the cells certainly appear smaller in the knockdown condition, it also appears that Figure 4I shows fewer planes as a z stack than Figure 4H (the image in I is less blurry). There could be other factors contributing to this blurriness, but I think a quantification of nuclear size would be useful.

All pictures were shown at the same magnification, and we have now included the scale bars, we apologize for this mistake in our previous submission. We agree with the reviewer, and have quantified the effects on salivary gland size and nuclear size in two different *salm/salr* knockdown genotypes. These data are included in Figure 5I-K. We have also rephrased the description of the mutant phenotypes for clarity:

<u>Page 16, added</u>: The combination *AB1-Gal4/UAS-salm-RNAi; UAS-salr-RNAi* causes the stronger phenotype, in which salivary glands are very small and are formed by fewer and smaller cells than normal glands (Fig. 5D; G-H; I-K). A weaker version of this phenotype is observed in the combination *sal*^{EPv}*-Gal4/UAS-salm-RNAi; UAS-salr-RNAi* (Fig. 5C; F; I-K).

16) *The authors state that they "searched for links between the transcriptional changes observed in salm/salr mutant discs" (p. 16), but it should be "RNAi discs".*

Changed "mutant" for "knockdown" in page 16.

17) The plots in Fig. 5A would work better as pie charts and venn diagrams as in Fig. 1.
I am not entirely sure about the logFC values given by the authors - could this please be double checked? The paper they are referring to for the irradiation-induced gene expression changes (van Bergeijk et al., 2012) seems to give fold changes, not logFC. So "genes overexpressed after irradiation with logFC in the range of 1 to 6" (page 16) would actually be a 0 to 0.8 logFC if it is "genes overexpressed after irradiation with a fold change in the range of 1 to 6". Otherwise, a logFC of 6 would mean a 1,000,000 fold change in expression, which seems very high. In general, I

© 2025. Published by The Company of Biologists under the terms of the Creative Commons Attribution License (https://creativecommons.org/licenses/by/4.0/).

am more accustomed to changes in expression being given as log(2)FC, but this may be dependent on the data. In any case, if it is indeed logFC, this also needs to be changed in Figure 5B.

We have changed the data representation in Fig. 5A to venn diagrams. For consistency with the rest of the manuscript, we have considered in these analyses the number of genes increasing or decreasing their expression with a logFC of 0.5 in the *spalt* RNA seq experiments. The results obtained with this data set compared with the one we first submitted are very similar, and show the best overlap for genes selected as increasing their expression in *spalt* knockdowns and after irradiation. The reviewer is correct in that the Bergeijk et al., 2012 paper uses fold changes in the tables included in the manuscript. However, they use *logFC* in their Supplementary Tables, which are the source of the data we used for the comparisons. In fact, the link to Supplementary Table 1, which is the one showing all differences they found in their experiments, is wrong in the journal web page, and the corresponding author (Dr Tin Tin Su) was kind enough to send us the correct version of this table, which is the one we used to extract the data displayed in our supplementary Table 2. If I am correct, a logFC of 6 (the highest in their experiments for the gene *Corp*) actually corresponds to a fold change of 70, which is more reasonable. A fold change of 1.000.000 would give a logFC of 19,93.

We have added the following sentence to material and methods:

<u>Page 25, added</u>: "We also selected a set of genes whose expression in the wing disc changes ±1.5-fold (pvalue<0.005; log(2)FC ±0.585) 2 or 18 h after 4000R irradiation (van Bergeijk et al., 2012). This set of genes was taken from Table S1 of Bergeijk et al. (2012), and was kindly provided by Dr Tin Tin Su. We selected 894 and 1021 genes as overexpressed or repressed, respectively after irradiation. The changes in gene expression observed after irradiation for about 80% of these genes depend on the presence of p53 (van Bergeijk et al., 2012)."

17) *The authors need to cite the publication where they mention the "two cases where we identified the enhancer region of the corresponding gene that became active upon a loss of salm/salr function" (page 16). They also state that "the same enhancers also became activated after irradiation". Has this been done as a part of this study? I don't think it is mentioned in the methods.*

We have included the reference to the identification of regulatory regions and reporter constructs, and the irradiation conditions in material and methods:

<u>Page 23, added</u>: "We also used a chromosomal deficiency for the *salm* and *salr* genes (Df(2L)32FP5; Barrio et al., 1999) and the reporter constructs *Gadd45 pHP-Dest-eGFP* and *ver pHP-Dest-eGFP* (Ostalé et al., 2024).

Irradiation of third instar larvae

Gadd45 pHP-Dest-eGFP, ver pHP-Dest-eGFP and wild type third instar larvae were irradiated (4000R) in a Phillips MG102 X-ray machine, and the wing discs were dissected 2 hours after irradiation and the expression of GFP and Dp53 was visualized in the confocal microscope with the same settings of Intensity levels and Pinhole for control and irradiated discs."

18) *Finally, they state that "The increase in expression levels observed after irradiation depends on the transcriptional regulator Dp53". I am not sure what they mean here. Is this an experiment they did? Is this activation of Dp35 independent from Spalt function? How is it related to Spalt?*
This conclusion is based on the data presented by van Bergeijk et al. (2012), where they study gene expression in response to irradiation in a *p53* mutant background. They describe that most changes observed after irradiation are lost in a *p53* mutant background. We have made the following changes to clarify this point:

<u>Page 25, added</u>: "We also selected a set of genes whose expression in the wing disc changes ±1.5-fold (pvalue<0.005; log(2)FC ±0.585) 2 or 18 hr after 4000R irradiation (van Bergeijk et al., 2012). This set of genes was taken from Table S1 and was kindly provided by Dr Tin Tin Su. We selected 894 and 1021 genes as overexpressed or repressed, respectively after irradiation (Supple. Table 2).

© 2025. Published by The Company of Biologists under the terms of the Creative Commons Attribution License (https://creativecommons.org/licenses/by/4.0/).

The changes in gene expression observed after irradiation for about 80% of the genes depend on the presence of p53 (van Bergeijk et al., 2012).”

19) Citations are missing from the Figure 5 legend. "salm/salr mutant discs showing ectopic expression in the central domain of the wing disc" (Organista et al., 2015) and citation for the images of upregulated genes. Is this form of republication of figures that have already been published (even if only in a supplementary figure) allowed?

We have included the reference to Organista et al., 2015 in the legend to Figure 5 (now Figure 6). We are aware that we are using data previously presented as supplementary information in a compilation of all genes we studied by *in situ* hybridization (see material and methods). We decided to use these selected data here because they are very much related to the topic under discussion in this work. However, we will of course abide to any editorial decision against the use of these data in this form in Figure 6.

20) In the discussion (page 19), the authors state that "changes in chromatin accessibility are poorly correlated to the changes in gene expression detected in salm/salr mutant wing discs." How can those two seemingly independent effects of Spalt be reconciled? What is the relationship to native Spalt binding? Can this be an effect of the different experimental setup (very precisely timed RNAi in the expresion analysis)? Or does it come down to secondary effects of a gene regulated by Spalt? Are any of the mis-regulated genes known to be involved in the regulation of chromatin accessibility?

We have made use of the Sal binding data to illustrate the discrepancies between binding with chromatin accessibility and gene expression (see new Fig. 1 and Supple Table 1). We have also added more discussion to this issue in page 20.

<u>Page 20, added</u>: “The poor correlation observed between global gene expression with changes in chromatin accessibility in *salm/salr* knockdown conditions suggest that loss of Sal function indirectly triggers multitude of gene expression alterations that are not caused by direct regulation of target genes by Salm/Salr binding.”

21) In Materials & Methods/Gene expression analyses (page 23), the number of genes with increased expression must be 922, not 822 (265+60+597). Again, the logFC values are a bit odd. 0.22 equates to 1.66-fold change, which seems very arbitrary. Why was this chosen as a cutoff? Also, a logFC of 5.26 equates to a 182,000-fold change in expression, which seems exceptionally high. Can this please be double checked?

We have set new cutoffs values for the expression data, selecting only those genes showing pvalue below 0.003 and log(2)Fold change equal or larger than 0.5. In our previous selection genes were only selected by pvalue. These changes affect Fig. 1K and L, Fig 6A, Supplementary Tables 1 and 2 and the material and methods section. The new selection criteria reduce the number of genes used in the comparisons, but still the percentages of coincidences between the different datasets are very similar.

22) In Materials & Methods/Transposable elements expression analysis (page 24), the genotypes of the embryos need to be stated.

We included the genotypes in page 26 (material and methods):

<u>Page 26, added</u>: “RNA from 0-2 hours old embryos from *Df(2L)32FP5)/CyO* and wild type parents were extracted using the GE Healthcare extraction kit. We used wild type embryos as controls, as there is no likely effect of balancer chromosomes (*CyO*) on the expression of retrotransposons (Ghavi-Helm et al., 2020).”

23) In Materials & Methods/Quantification of eye pigment (page 24), the authors state that "Three different measurements are performed". I presume they mean extractions?

Three independent extractions were made and measured. We changed the corresponding sentence in page 27.

© 2025. Published by The Company of Biologists under the terms of the Creative Commons Attribution License (https://creativecommons.org/licenses/by/4.0/).

24) In Materials & Methods/ATAC-Seq (page 25), the authors need to give more detail about the stage at which imaginal discs were dissected. Also, the genotypes have to be corrected - they are different from the description in the rest of the manuscript.

Corrected in pages 28 and 29

25) There seems to be a doubling of the 30 m incubation at 4 C.

Size-selection with AMPure XP beads using 0.5 - 1.8x - does this mean two steps? First 0.5 to remove large fragments, then 1.8 to retain small ones?
A depth of 50000 reads would be very shallow. I presume this is a typo and should be 50,000,000 (or at least 5,000,000)?

We are submitting as Supplementary Figure 1 the mapping stats (bowtie2). This data show that we had between ~11 and 15 million uniquely mapping reads per experimental sample and 19-23 reads for input controls.

We have also corrected in the ATACseq protocol:

Page 26, corrected: "The DNA fragments were size-selected with AMPure XP beads using a double-sided size selection with a right-side clean-up ratio of 0.5x, and a left-side clean-up ratio of 1.8x (Beckman Coulter, A63881). The two steps clean-up was performed as described by the manufacturer."

25) There is no mention of sequencing depth for H3K27ac ChIP-seq.

See above.

26) Materials & Methods/Comet assay: How many replicates were performed?
Materials & Methods/Bioinformatic analyses: As mentioned above, this needs more detail (programs, versions, parameters where non-standard).

Added to page 30: "We conducted two independent experiments that included all analyzed conditions, with each sample containing at least 40 wing imaginal discs. Additionally, two technical replicates were performed per sample, scoring a minimum of 100 comets per gel, for a total of 200 comets per slide."

27) Materials & Methods/Statistical analyses: Most plots show no significance values. For some charts chi-squared test may be better (e.g., Fig. 1E, F)?

The numbers in Figures 1E and F for samples of similar size are so different that we considered no necessary to make statistical analyses.

28) Minor comments/typos:

-page 3: "tracheal dorsal trunk formation" - might be better to say "formation of dorsal trunk trachea" (if that is what the authors mean here).

Changed

-page 4: "The multiplicity of functional requirements [...] underly the association of [...]" The sentence doesn't really make sense and should be rewritten.

Changed to: "The multiple functional requirements for Sal proteins in the assignation of cell fates can be related to the association of human SALL1 and SALL4 with the genetic syndromes Townes-Brocks (SALL1; Kohlhase, 2000; Kohlhase et al., 1999) and Okihiro (SALL4; Borozdin et al., 2004), which are characterized by a variety of malformations in the limbs and different internal organs."

© 2025. Published by The Company of Biologists under the terms of the Creative Commons Attribution License (https://creativecommons.org/licenses/by/4.0/).

-page 4: "located 11 Kb from the kni transcription start site (Ostalé et al., 2024) and the C. elegans" would red better as two sentences: "located 11 Kb from the kni transcription start site (Ostalé et al., 2024). Likewise, the C. elegans"

Done

-page 5: "cell-type specific gene target promotors" should be "cell-type specific target gene promotors"

Done

-page 7: "We found that loss of salm/salr function also has a broad impact on the presence of the H3K27ac mark in imaginal cells. Thus, ChIP-seq ("Chromatin immunoprecipitation followed by sequencing") experiments with anti-H3K27ac antibodies in control and salm/salr knock-down discs reveal a similar number of chromatin regions showing increased or decreased H3K27 acetylation" - These two sentences appear the wrong way round?

We changed the sentence for clarity to:

Page 7, changed: "We found that loss of *salm/salr* function also has a broad impact on the presence of the H3K27ac mark in imaginal cells. Thus, ChIP-seq ("Chromatin immunoprecipitation followed by sequencing") experiments with anti-H3K27ac antibodies in control and *salm/salr* knock-down discs reveal chromatin regions showing increased (446 peaks, FDR=0.05) or decreased (585 peaks, FDR=0.05) H3K27 acetylation"

-page 8: "Analyzing the sets of regions that suffer epigenetic modification" - I would suggest to write "whose accessibility changes" or so.

Done

-page16: "reasonably overlap" should be "reasonable overlap"

Done

-page 17: "presumptive longitudinal L2 vein" should be "future longitudinal L2 vein"

Done

-page 18: "It was also unknown" should probably be "It is also unknown"?

Done

-page 23: AB1-Gal4 and UAS-mCD8-GFP don't have a stock number.

Done

-page 28: "Inmunocitochemistry" should be "Immunohistochemistry"

Done

-page 28: "A total of 40 wing imaginal discs cells per replicate" should be "A total of 40 wing imaginal discs per replicate"

Done

-page 41: "salm/salr mutant wing imaginal discs" should be "salm/salr RNAi/knockdown wing imaginal discs"

Done

© 2025. Published by The Company of Biologists under the terms of the Creative Commons Attribution License (https://creativecommons.org/licenses/by/4.0/).

Reviewer 2

SUMMARY OF THE ADVANCE MADE IN THIS PAPER AND ITS POTENTIAL SIGNIFICANCE TO THE FIELD

In this manuscript, Ostale et al. investigate the dual roles of phylogenetically conserved Spalt proteins in Drosophila development. These proteins are known to regulate gene expression and influence cell fate by acting as transcriptional repressors within gene regulatory networks. Interestingly, Spalt proteins also localize to heterochromatic regions, suggesting a role in epigenetic regulation. In this study, the authors describe the impact of Spalt protein loss in Drosophila cells, noting changes in chromatin accessibility, DNA damage, chromosome localization in polyploid salivary gland cells, and misexpression of transposable elements. They propose that Spalt proteins function both as sequence-specific transcriptional repressors and as regulators of global gene silencing through heterochromatin formation. In principle, the topic of this study is of general interest, as sequence-specific transcription factors like Spalt are just starting to be recognized as organizers of heterochromatin. However, this manuscript is currently not in a state to be published in Development due to several major and minor issues.

SUGGESTIONS TO AUTHORS

Major Issues:

1. In the abstract, the authors highlight the interaction of Spalt with specific epigenetic regulators like NuRD or LSD1, which is not the topic of the paper. This should be toned down, as one expects that this interaction is at least partly analyzed in this research.

We have modified the sentence referring to NuRD and LSD1 in the summary to restrict its application to vertebrate Spalt proteins, where these interactions have been thoroughly analyzed.

<u>Page 2, added</u>: “Vertebrate Spalt-like proteins can…”

2. The language is sometimes not correct and should be checked.

We have checked the language to the best of our ability.

3. Has the interaction of Drosophila Spalt with heterochromatin been shown before, as on page 5 only vertebrate Spalt is mentioned? The same is true for the interaction with heterochromatic proteins.

The interaction of *Drosophila* Spalt major with heterochromatin, to our knowledge, has only been previously reported through Salm chromatin immunoprecipitation experiments in the paper Ostalé et al., 2024 (https://doi.org/10.1016/j.ydbio.2024.03.004). We have rewritten this sentence for clarity:

<u>Page 5. Changed to</u>: “On the other hand, Salm in *Drosophila* (Ostalé et al., 2024) and SALL1 and 4 in vertebrates (Netzer et al., 2001; Netzer et al., 2006; Sakaki-Yumoto et al., 2006) preferentially locate to heterochromatic regions, and SALL1 interact with several heterochromatic proteins (Netzer et al., 2001).”

4. A major claim of the paper is that there is only a minor overlap of changes in chromatin accessibility and changes in gene expression in wing discs after spalt knock down. Where is this data shown? It would be important to backup this statement with real data. Is this the case for both activated and repressed genes by Spalt?

We have considerably modified the first section of the manuscript following the suggestions of reviewer 1, and we explain how these calculations were made. The data comparing gene expression in *salm/salr* knockdown conditions and the results of ATAC-seq and H3H27Kac ChIP are now shown in Figure 1K. This Figure panel includes genes up and down regulated in *spalt* knockdown conditions (sal-UP and sal-DOWN) as well as genes with increased or decreased accessibility (ATAC-UP and ATAC-DOWN or with increased or decreased H3K27 acetilation (H3K27ac-UP and H3K27ac-DOWN). To do these comparisons we first have to associate changes in accessibility and H3K27ac to the

© 2025. Published by The Company of Biologists under the terms of the Creative Commons Attribution License (https://creativecommons.org/licenses/by/4.0/).

likely associated gene (using as criterium genomic proximity). The numerical data supporting these calculations are presented in Suppl. Table 1.

5. How many of the observed changes in chromatin accessibility are a direct consequence of loss of Spalt binding? In other words: How many of the identified ATAC regions in control are bound by Spalt (according to the recent published paper from this lab)? How many of these Spalt bound regions show changes in chromatin accessibility in Spalt knock down conditions in comparison to all the changes in chromatin accessibility observed?

The answer to this question is that very few changes in chromatin accessibility or H3K27 acetylation are associated to Salm binding. The actual numbers are:

-22 genes associated to sequences with increased accessibility are also associated to Salm binding

-4 genes associated to sequences with reduced accessibility are associated to Salm binding.

We have included this data in Supplementary Table 1, and include a Supplementary Figure 3 showing the localization of ATAC (ATAC-UP), Salm ChIP and H3K27ac (H3K27ac-UP) sequences in the 7 cases where we found a coincidence. With this data, we have to suggest that most of the changes in chromatin accessibility caused by loss of *salm/salr* function are likely indirect.

<u>Page 20, added</u>: “The poor correlation observed between global gene expression with changes in chromatin accessibility in *salm/salr* knockdown conditions suggest that loss of Sal function indirectly triggers multitude of gene expression alterations that are not caused by direct regulation of target genes by Salm/Salr binding.”

6. One histone mark enriched in pericentromeric heterochromatin regions is H3K27me3, which should be reduced in Spalt knock down conditions. The authors should backup their study with profiling H3K27me3 in spalt knock down wing discs.

This is a very relevant experiment, and belong to a more in-depth analysis of the relationships between Spalt proteins and chromatin marks. While we are now in the process of starting these analyses, we ask for the reviewer understanding that the suggested experiment (profiling H3K27me3 in *spalt* knockdown wing discs) while very relevant, falls outside of the scope of the present manuscript.

7. The author found that chromatin regions that change their accessibility after spalt knockdown are bound by HP1. What is the overlap of Spalt and HP1 binding in wild type wing discs? How much of HP1 binding is lost in Spalt KD conditions? Do Spalt and HP1 interact with each other?

We are not aware of the existence of protein interactions between HP1 and Spalt. We have now included the relationships between the ATAC and H3K27ac data with HP1 (and H3K9me3) ChIP datasets previously published (see new Figure 2). We only see some clear overlaps between sequences identified as both ATAC-UP and H3K27ac-UP with HP1 and H3K9me3, and a less strong overlap between sequences identified as both ATAC-DOWN and H3K27ac-DOWN with HP1 and H3K9me3 (see new Fig. 2). It is not known whether binding of HP1 is dependent on Spalt function.

8. The information contained in Fig. 1G - K is unclear.

We have made an entirely new Figure 1, which we hope reflects better the overlaps we can detect between different datasets.

9. The authors jump between cellular models, from wing disc, to eyes, to embryos, to salivary glands, without stating why these particular systems were chosen for the specific experiment. Why were for example 0-2 h embryos taken to study retrotransposon activation in embryos (Page 12, first paragraph)? In the figure showing the result (Fig. 2F), the x-axis is not labelled and it is only clear from the Materials and Methods that the expression of retrotransposon activation was measured by qPCR.

© 2025. Published by The Company of Biologists under the terms of the Creative Commons Attribution License (https://creativecommons.org/licenses/by/4.0/).

We used different cellular models by reasons of experimental convenience to study the effects of *salm/salr* mutants and knockdowns. For example, early embryos allow the use of a *salm/salr* deficiency, salivary glands combined with *salm/salr* knockdown are much more convenient to study nuclear morphology, eyes to study the expression of heterochromatic *Pw+* insertions and the imaginal disc to compare different datasets related to gene expression and chromatin modifications. We have now included a sentence at the end of the introduction to better justify the use of different experimental systems that only have in common the expression of the *salm* and *salr* genes.

Page 6, modified: "Here we analyzed in different *Drosophila* tissues that have in common the expression of *salm* and *salr* the possible contributions of Sal proteins to chromatin organization as a mechanism to account for the variety of *sal* developmental roles."

We inserted the label in Fig. 2F (now 3F)

We also changed the initial paragraph of this section of material and methods to:

Page 25, inserted: "We used real time PCR, performed with a Bio-Rad CFX Opus 384 machine, to quantify the amount of F-element, TART-A, Het-A and Rpl32 mRNA in normal and salm/salr deficient embryos."

10. The authors then jump from embryos to salivary glands to look at the nuclear localization of HP1 after spalt knockdown. The authors claim that HP1 localizes here (Fig. 1G, G') to a single spot which corresponds to the chromocenter. There is no co-labelling nor a citation that supports this claim. In Fig. 2H, H', DAPI staining is different compared to the control (2G, G'), as the center of the nucleus is free of DAPI. What does this mean? Changes in distribution of chromatin?

-We now included a citation to a manuscript describing the accumulation of HP1 in the chromocenter of salivary glands nuclei (page 37; https://doi.org/10.1002/dvdy.20310).

-The reviewer is correct, and we argued that one of the consequences of *salm/salr* knockdown is the formation of larger size nucleoli and the peripheral localization of the DNA within the nucleus (see cartoon in Fig 6A-B, now Fig. 7A-B).

11. The authors then jump again to blastoderm embryos to study mitotic defects in spalt heterozygous mutants. A brief statement why this model now would be very helpful. The authors claim to see the formation of anaphase bridges from DAPI stainings. Additional stainings of the microtubule would be necessary to substantiate this claim. What is the amount of these defects? Quantifications are missing.

We inserted the following sentence:

Page 12, inserted and modified: "The *Drosophila* blastoderm is a very convenient system to analyze mitotic defects, because in normal embryos nuclear divisions are synchronic (Foe, 1989)."

We also quantified the number of mitotic errors observed in mutant *salm/salr* embryos, and this quantification is now shown in Fig. 3L.

We understand that conventional anaphase bridges are formed by chromatin and can be visualized with DNA dyes such as DAPI. There are other types of bridges ("ultra-fine anaphase DNA bridges") that require the use of antibodies against proteins that bind to then, but we are limiting our observations to DAPI-positive conventional anaphase bridges.

12. In all figures, scale bars are missing.

We apologize for this mistake, and have now included the scale bars in all figures.

13. The authors then perform DNA damage analysis in wing discs. Why this model for this question? The authors should perform gammaH2Av stainings, which is a marker for DNA damage to directly

© 2025. Published by The Company of Biologists under the terms of the Creative Commons Attribution License (https://creativecommons.org/licenses/by/4.0/).

visualize DNA damage in the wing disc. It is unclear what Fig 3B-C should show. What does DNA in tails and Tail moment (Fig. 4) mean? It is unclear what is shown in this figure.

A) The wing imaginal disc is constituted by diploid cells that exhibits a canonical response to DNA damage caused by irradiation, triggering the DDR pathway and eventually apoptosis. The disc can be easily extracted from L3 larvae, and its cells can be isolated through enzymatic digestion without compromising the integrity of the genetic material. All these features make it an ideal model for the evaluation of DNA integrity. The wing disc is also the tissue where we can make all the genomic comparisons related to gene expression.

We have added the following sentences:

Page 13 added: ·The wing imaginal disc is a proliferative epithelial tissue constituted by diploid cells that exhibits a canonical response to DNA damage caused by irradiation, triggering the DDR pathway and eventually apoptosis (Baonza et al., 2022)."

B) We have included a staining with H2Av, which is included in Figure 4 I-J, and have added the following paragraphs to the text to comment on these results:

Pages 14-15, added: "The incorporation of γ-H2AX (H2Av in *Drosophila*) to chromatin is a primary response to DNA double-stranded breaks (Rogakou et al., 1998). We found that *salm/salr* knockdown wing imaginal discs display ectopic P-H2Av in a small fraction of wing cells (8 +/-2 cells per wing disc, a situation never detected in wild type wing discs of the same age (Fig. 4I-J). Because the results of the comet assay show extensive DNA damage, we suggest that most of these alterations should correspond to single-strand DNA breaks."

C) The level of DNA damage is inferred from the extent of DNA migration. After scoring, image analysis can be plotted according to various descriptors; tail length, tail intensity (also referred to as percentage of DNA in tail) and tail moment. They give rise to results expressed in different units. The tail length is proportional to the extent of DNA damage but reaches its maximum at a relatively low level of damage. Tail intensity is expressed as percentage of total DNA fluorescence in the tail of the comet. Tail moment is calculated as the product of the tail length and the fraction of total DNA in the comet tail. The tail intensity is currently recommended by the OECD as the best descriptor for DNA break frequencies since it uses a quantitative measure of damage (from 0% to 100%) (Vähäkangas et al., 2014). However, several researchers still tend to use tail moment, since it takes into account both the length and DNA content of the comet tail. Tail moment has the disadvantage of not having standard units. Due to these factors, we chose to represent DNA damage using both tail intensity and tail moment to provide a comprehensive assessment. To improve clarity, we have made the following changes to material and methods:

Page 33, modified: "We represented DNA damage using both tail intensity (percentage of DNA in comet tails) and tail moment (the product of tail length and tail intensity), to provide a comprehensive assessment of the changes observed in DNA migration."

14. Again, no scale bars in Fig 4, so it is unclear whether there are any changes in the size of the salivary gland. For a non-expert, it is impossible to judge how a salivary gland looks like and what to expect to see.

We have made considerable changes to Figure 4 (now Figure 5), adding the scale bars and quantifying salivary gland size, nuclear size and number of cells in wild type and *salm/salr* knockdown salivary glands.

15. What are "hippomorphic conditions" (page 15)?

We have modified the description of the *salm/salr* knockdown phenotypes in the salivary glands to:

Page 16, added and modified: "The combination *AB1-Gal4/UAS-salm-RNAi; UAS-salr-RNAi* causes the stronger phenotype, in which salivary glands are very small and are formed by fewer and smaller cells than normal glands (Fig. 5D; G-H; I-K). A weaker version of this phenotype is observed in the combination *sal^{EPv}-Gal4/UAS-salm-RNAi; UAS-salr-RNAi* (Fig. 5C; F; I-K)."

© 2025. Published by The Company of Biologists under the terms of the Creative Commons Attribution License (https://creativecommons.org/licenses/by/4.0/).

Additional comments:

1. Page 5: It would be nice if authors can cite or mention few interactors or a study that probes the interaction partners of sal proteins to give more context splat functionality.

We have made minor changes to include the best characterized interactions of SALL proteins.

Page 5, modified: “In addition, numerous studies identified interactions between SALL1 and SALL4 proteins and the Nucleosome Remodeling and Deacetylase (NuRD) complex, suggesting a pivotal involvement of NuRD as a central facilitator in the repression mediated by SALL1 and SALL4 (Kiefer et al., 2002; Lauberth and Rauchman, 2006; Lauberth et al., 2007; Wang et al., 2023).”

Page 5, modified: “Finally, Sall4’s role as a transcriptional repressor has also been linked to its interaction and recruitment of the lysine-specific histone demethylase LSD1”

2. Page 7: How efficient is the salm/salr-RNAi lines? Is there already a validation present in previous research work and if not, did the authors validate the extent of RNAi? The choice of RNAi over knockout can be explained for this context as well. Is the knockout of salm/salr embryonic lethal?

We have used in several previous manuscripts the *UAS-salm* and *UAS-salr RNAi* lines, but we have never quantified by real time PCR the extent of *salm* or *salr* transcripts reduction. The only evidence we have to suggest that these lines are effective is that they strongly reduce Sal protein expression (Ostalé et al., 2024), and that the resulting phenotype of *salm/salr* mosaic mutant wings and *salm/salr* doble knockdown are very similar (Organista and de Celis, 2013). We have included the following statement to emphasize this issue:

Pages 24-25, added: “The expression of *UAS-salm-RNAi* strongly reduces Salm expression (Ostalé et al., 2024), and the knockdown of *salm* and *salr* in the wing disc results in wing phenotypes similar to those associated to a *salm/salr* deficiency (Organista and de Celis, 2013), indicating that these lines are very effective to generate *salm* and *salr* knockdowns.”

3. Page 8: I think authors can mention here that, both distal intragenic and between gene regions have been indicated as distal regions in the figure.

Pages 7-8, modified: “we found a large number of enriched sequences located more than 5 Kb from the nearest transcription start site (TSS), including those located within large introns (distal intragenic) or between genes (distal intergenic).”

4) Page 8: The x-axis labelling in figure F (referred from this page number) is confusing. The figure legend says the order is ATAC-UP, ATAC-DOWN, H3K27ac-UP, H3k27ac-DOWN, whereas on x-axis in the figure it is H3K27AC-DOWN before H3K27ac-UP.

We have made many changes to the narrative of this section of the manuscript, and we expect that these mistakes have been corrected.

5) Page 9: This should be Figure 1F and not figure 2F (labelling/reference mistake).

Corrected

6) Page 9: Figure F' and Figure F" instead of Figure 1F in line 22 and 24

Corrected

7) Page 10: the figure says H3K29ac, should not it be H3K27ac?

Corrected

8) Page: 10 Paragraph second: This is very confusing to read, should be rephrased or made simpler.

© 2025. Published by The Company of Biologists under the terms of the Creative Commons Attribution License (https://creativecommons.org/licenses/by/4.0/).

This section has been extensively modified and we hope that is clear now.

9) Page 10: Can the authors give a rationale behind choosing significant expression change using p-value over fold change in gene expression? Could it be related to the fact that the knockdown, rnai is inefficient?

Our rationale was not related to the efficiency of the RNAi. We selected the set of genes displaying significant changes, irrespectively of the magnitude of change, because we though this set might better reflect relationships between changes in gene expression and chromatin modifications. We have entirely remade this section and the comparisons between datasets, and now are using the Fold change as a selection criterion. In general, we observe very similar relative relationships between different datasets when comparing *salm/salr* RNAseq, expression after irradiation, or modifications in ATACseq or H3K27ac ChIPseq data.

10) Page 10 last paragraph/11 first paragraph: The authors should discuss possible limitations that could have affected the approach to this conclusion. Like the gene association by the window of distal regions and incomplete effects of rnai. That would also strengthen the conclusions which right now seems very broad.

Page 29, added: "A caveat to this conclusion is that our analyses have been carried out in knockdown conditions, which likely represent a partial loss of gene function, as well as the criterion of proximity to the nearest TSS used to associate the sequences identified in the ATAC and H3K27ac IP experiments to the affected transcript."

11) Page 14: Tail moment seem similar in salm salr rnai vs wt discs from the plot and seem significantly different in h2o2 discs? Should not the plot look similar to % DNA plot (in context of wt vs mutant vs H2O2 discs)?

Tail movement shows that cells in the *salm/salr* knockdown discs fall in two populations, one with DNA damage (upper values) and one with very similar values to cells obtained from wild type discs. This is expected, because only a fraction of the cells in *salm/salr* mutant discs are expected to display DNA damage. These cells are those expressing the Salm/Salr proteins included in the domain of Gal4 expression where we direct the knockdown. We describe this fact in this paragraph:

Page 14: "The expression of *sal*^{EPv}*-Gal4* is restricted to the central domain of the wing blade (Fig. 4A). Consequently, cells dissociated from these discs contains *salm/salr* knockdown cells (wing cells from the domain of *salm/salr* expression), normal wing cells lacking expression of the *salm/salr* genes, and cells from the wing hinge and thorax that express *salm/salr* but do not express the corresponding RNAis and therefore have normal Salm and Salr function."

12) Page 14 (Figure C G-H'): There should be quantifications for these images. It seems pretty clear but could be misleading because of the background in the images.

We made the quantification in Fiji and include the values in page 15:

Page 15, modified: "The expression of P53 is detected at similar levels in all cells of the wildtype wing disc (average intensity levels/area of 13,9 +/- 3.1; Fig. 3G-G'). In contrast, we found that the levels of P53 protein are increased in the central region of the wing blade in salm/salr mutant discs (average intensity levels/area of 20.0 +/- 4.7; Fig. 3H-H')."

13) Page 15: Is the weak knockdown that authors talk about because of the RNAi used specifically in this case?

No, this is because the Gal4 driver we used. It should be clearer in the new writing:

Page 16, added and modified: "The combination *AB1-Gal4/UAS-salm-RNAi; UAS-salr-RNAi* causes the stronger phenotype, in which salivary glands are very small and are formed by fewer and smaller cells than normal glands (Fig. 4D; G-H; I-K). A weaker version of this phenotype is observed in the combination *sal*^{EPv}*-Gal4/UAS-salm-RNAi; UAS-salr-RNAi* (Fig. 4C; F; I-K)."

© 2025. Published by The Company of Biologists under the terms of the Creative Commons Attribution License (https://creativecommons.org/licenses/by/4.0/).

14) Page 16 (Figure 5C): Was there an in-situ done for gadd45 and ver as well in case of salm/salr rnai? Because that would to more concretely connect the xray irradiation response and sal deficiency response.

Gadd45 and *ver* in situ hybridization in wild type and *salm/salr* mutant discs are published in Ostalé et al., 2024. We have made the following addition to this issue:

Page 17, added: "The mRNA expression of these genes is detected at high levels in the central region of the wing disc in *salm/salr* knockdown conditions (Ostalé et al., 2024)."

15) No scale bars in images: Specifically, required in figure 4 for the context of size of salivary gland. Figure H and I seems to be taken at different magnification.

We apologize for this mistake, and have now added the corresponding scale bars to the figures. In addition, we have quantified the phenotype of salm/salr knockdown salivary glands (Fig. 5I-K).

In summary, the manuscript presents a collection of descriptive findings without pursuing the intriguing results further, leaving significant gaps in the research narrative. The rationale for selecting specific models remains unarticulated, and the figures are assembled with a lack of precision and coherence. The overall impression is one of disparate research elements compiled into a single manuscript, rather than a cohesive and well-integrated study. Consequently, I find myself unable to recommend this manuscript for publication in Development.

We value the reviewer view, and have made our best efforts to reinforce the rationale behind the selection of different experimental models and the integration of the different results. We also hope that we have managed to produce a better-integrated study that, even though remaining mostly descriptive, contains enough information and proposals to direct the research on *spalt* genes into new directions.

Reviewer 3

SUMMARY OF THE ADVANCE MADE IN THIS PAPER AND ITS POTENTIAL SIGNIFICANCE TO THE FIELD

In this manuscript, the authors provide evidence that in addition to their function as sequence-specific transcriptional repressors, Spalt proteins also regulate gene expression more globally by generation or maintenance of heterochromatic domains. The data suggest that loss of Spalt function results in changes in chromatin organization. The claim that Spalt proteins are required for maintenance of genomic DNA integrity is strongly supported by the data provided. Loss of spalt function could lead to mitotic errors in mutant diploid cells resulting in a delay of cell cycle progression and to morphological changes in mutant polyploid cells. Interestingly, in addition to heterochromatic regions that become more accessible upon salm/salr knockdown, a similarly high number of genomic regions becomes less accessible suggesting that Spalt proteins also have other functions not discussed in this paper. Thus, the data generated in this paper could provide a base for further research on the role of Spalt proteins not only in flies, but also in mammalian systems.

SUGGESTIONS TO AUTHORS

Essential revisions

1) Given the multitude of genes and genomic regions affected by the loss of Spalt proteins and the severe changes in nuclear organization observed in cells after salm/salr knockdown, it is surprising that Spalt expression is not required in all cells. How is DNA damage and chromatin mis-organization prevented in the cells of the wing disc that do not express Spalt? Or, in other words, why do the cells in the center of the wing disc require Spalt proteins, while those in the periphery do not?

This is a key point that we didn't comment in the previous submission because at this stage we can only offer speculate. Thanks to the reviewer question, we are now including the following

© 2025. Published by The Company of Biologists under the terms of the Creative Commons Attribution License (https://creativecommons.org/licenses/by/4.0/).

sentences in the discussion, to at least comment on the possibility we consider more plausible to account for this caveat.

Page 24, added: "The novel functions we are suggesting for *Drosophila* Sal proteins in the maintenance of heterochromatic domains and genome stability are likely required in all cells. However, the expression of the *sal* genes in the wing disc is restricted to particular territories, such as the central region of the wing pouch, the wing hinge and the presumptive lateral thorax. This raises an intriguing caveat, related to how the functions of Sal proteins are exerted in cells where the corresponding genes are not expressed. At this time, we can only speculate that perhaps other DNA binding proteins might be able to perform similar molecular functions related to chromatin biology in cells where Sal proteins are not expressed."

2) On page 15, the authors claim that there is a high variability in salivary gland size between the different genotypes they looked at (loss of salm/salr function in these cells causes a variety of phenotypes, ranging from rudimentary salivary glands to morphologically normal glands, depending on the particular Gal4/UAS-RNAi combination). It would be helpful to show this for example with box plots of salivary gland length (and width) and/or of nuclear diameters for the different genotypes.

We agree with the reviewer and have now quantified salivary gland size, as well as nuclear size and estimated number of cells in different genotypes (see Fig. 5 I-K).

3) I could not find a reference for the Gadd45 and ver reporter constructs in the manuscript, nor where they described in the materials and methods section. Were the enhancer regions of Gadd45 and ver identified in this study? Are the Gadd45 RR-GFP and ver RR-GFP GFP reporters mis-expressed in salm/salr knockout cells? Is their expression in irradiated cells dependent on the presence of P53 protein?

These constructs are ectopically expressed in *salm/salr* mutant wing disc cells, and we don't know whether their expression depends on P53, although we expect this to be the case. We have now included the appropriate references to these constructs.

Page 24, inserted: We also used a chromosomal deficiency for the salm and salr genes (*Df(2L)32FP5*) (Barrio et al., 1999) the reporter constructs *Gadd45 pHP-Dest-eGFP* and *ver pHP-Dest-eGFP* (Ostalé et al., 2024).

Additional minor comments

1) In figure 1 panel F, the order of the conditions (in the x-axis) is switched between A3K27ac DOWN and UP as compared to panel E (and G, J, and K). Is this just an error in the labeling of the axis or is there really a smaller coverage of pericentric heterochromatin in the cases where acetylation goes up?

We have extensively changed Figure 1 and its description, and expect that now the accompanying narrative is clearer.

2) In the pie-charts in figure 1 panels H and I, the absolute numbers are given with the percentages in brackets (some brackets are missing in panel I), while in panels J and K the percentages are given first with the absolute numbers written below in a smaller font (n=). It would be nicer to have those displayed in a more consistent way throughout the entire figure.

See previous answer

3) Figure 2 panels A to E: in the main text (page 11) the authors state that the pictures are showing female eyes, whereas in the figure legend they claim that these are male eyes. In the materials and methods section (page 24), they just say they used 15 heads of each genotype for the quantification of eye pigment without specifying if these were males or females (or both).

We used female heads, and now mention this in the main text and figure legends.

© 2025. Published by The Company of Biologists under the terms of the Creative Commons Attribution License (https://creativecommons.org/licenses/by/4.0/).

In addition, in the main text: "...we found a consistent increase in eye pigmentation (P{hsp26-p-T} w[67c23] expression...", the w[67c23] is a mutation in the endogenous white gene on the X-chromosome, it is not expressed in these flies. The expression they compare is due to a mini-white marker in the inserted P-elements.

Changed in page 11: “a consistent increase in eye pigmentation (expression of the *miniwhite* gene present in *P{hsp26-pt-T}*) in *white* mutant females heterozygous….”

In the interest of consistency, it would also be nicer to have the results in panel E in the same order as in panels A to D (first the CyO in black, then the Df5 in grey). This would also be more consistent with panel F where the control (wt) conditions are shown in black and the Df5 conditions are shown in grey.

Done

4) I would assume, that the images in figure 2 panels M and N were taken with the same settings for the different channels, but this is nowhere stated in the text. Are the nuclei bigger in panel N than in panel M? Are they in the same replication phase?

All images were taken with the same confocal settings and the embryos are of the same age (cycle 12 blastoderms) and taken at the same magnification. We added the following sentence:

Page 49, added: “The confocal settings were the same in the pictures shown in panels M-N.”

5) In the figure legend for panel A of figure 3 (and 4) the authors write: "The expression of DAPI is shown in blue". DAPI is not a protein that is expressed, but a staining for DNA.

Changed

6) In figure 3 panels G' and H' I can see a higher P53 expression in the sal-domain, but the P53 levels are also increased outside of the central area in panel H as compared to G, and the cellular pattern looks different (less punctate). Where the images (especially the red channel) taken with the same settings? In the materials and methods section there is nothing written about these particular stainings or the acquisition of the images.

Page 50, added: “The confocal settings were the same in the pictures shown in panels I-J.”

7) Scalebars are missing in the entire paper. This is particularly irritating in figure 4 panels B to E, which are supposed to display differences in the size of the salivary glands of the different genotypes. Even in the materials and methods section there is nothing written about the acquisition of these images or how they were processed (e.g. cropped).

We apologize for this mistake, and have now added scale bars in all confocal pictures. In the particular case of the salivary glands shown in the new Fig. 5 E-H, the differences in size between the different genotypes was so manifest that we did not consider necessary to quantify. After the comments of all reviewers, we have now included the quantifications of gland area, nucleus area and estimated number of cells (Fig. 5 I-K).

8) For the TEM images in figure 4 panels F and G, the genotypes are not clear. In the figure legend the authors state that they used wt salivary glands (in panel F) and salEPv-Gal4 to express the RNAi constructs (in panel G), whereas in the materials and methods section (page 27) they mention 3 different genotypes ("Third instar larvae of phm-Gal4 UAS-GFP UAS-GFP (25 °C), phm-Gal4 UAS-GFP Gal80ts UAS-salm-i UAS-salr-i (2 days at 25 °C, 5 days at 17 °C and 5 days at 29 °C) and UAS-salmi/+; AB1-Gal4/UAS-salr-i (25°C) genotype") for this experiment, where none of them contains salEPv-Gal4.

This was our mistake. We have now corrected the genotypes in the material and methods and in the figure legends.

© 2025. Published by The Company of Biologists under the terms of the Creative Commons Attribution License (https://creativecommons.org/licenses/by/4.0/).

Page 32, changed: “Third instar larvae of *sal*^{EPv}*-Gal4 UAS-GFP/+* (25 °C) and *sal*^{EPv}*-Gal4 UAS-GFP/UAS-salm-i; UAS-salr-i/+* (25°C) genotype.”

9) In figure 4 panels H and I: The authors state that "the size of the nucleolus is much larger in salm/salr mutants compared to normal glands, and the chromosomes have an abnormal disposition within the nuclei". Does the nucleolus only appear bigger due to the smaller size of the nucleus? The authors should also mention somewhere that Fibrillarin is localizing to the nucleolus.

Page 54, changed: “the expression of the nucleolar marker Fibrillarin.”

We think the nucleolus is bigger in the mutants. In this particular case, the genotype we are using reduces nuclear size (see Fig. 5K), but still nucleoli are much bigger than in control cells.

10) In figure 5 panel A, the first 2 columns are both labeled "SAL UP". So, what is the difference there? Or is one of them supposed to display SAL DOWN?

Yes, it was an error. We have now changed this figure following the advice of reviewer 1 to use venn diagrams.

11) In figure 5 panels H and I, where the discs imaged with the same settings? Panel H would seem to be a better image to compare the even P53 expression pattern with the enhanced expression in the salm/salr knockdown disc in figure 3H.

Page 52,added: “The confocal settings were the same in the pictures shown in panels D-G and H-I.”

12) There is no reference to Supplementary figure 1 anywhere in the manuscript.

Page 29, added: “The correlation matrix of ATAC-seq samples, the overall enrichment of sequences in *sal*^{EPv}*-Gal4 UAS-GFP/UAS-salm-i; UAS-salr-i/+* versus *sal*^{EPv}*-Gal4 UAS-GFP/+*, as well as the sequence alignments of all ATAC-seq and H3K27ac Ch IP-seq experiments are shown in Supple. Fig. 1..”

The numbers in suppl. Table 1 do not exactly correspond to the numbers in figure 1 panels A and B. For example: there are 910 hits with an FDR below 0.001 listed in the ATAC_UP tab of the table. But only 899 hits given in the plot in panel A and only 73 of them are also UP in H3K27ac while only 5 are also DOWN in contrast to the 111 UP and 11 DOWN overlaps in panel C. Please explain.

We have now double-checked that the data in the Supplementary Tables 1-3 are accurately translated into the graphical representations shown in Figures 1, 2 and 6. In those cases where we used different numbers for particular comparisons, for example to calculate the fraction of genes in different datasets expressed or not in wild type discs, we state the actual numbers used. In this example, we only used those genes for which we have available the data of expression. We have remade most of Figure 1, and expect that the errors displayed before are now corrected.

13) On page 15 the authors wrote: "We focused our analysis in the salivary glands of salm/salr hippomorphic conditions (weak knockdown)". The correct word would be hypomorphic. I found some more spelling and grammatical errors, but this is the most relevant one, because it changes the meaning of the word.

We changed this sentence to:

Page 16, changed: “The combination AB1-Gal4/UAS-salm-RNAi; UAS-salr-RNAi causes the stronger phenotype, in which salivary glands are very small and are formed by fewer and smaller cells than normal glands (Fig. 4D; G-H; I-K). A weaker version of this phenotype is observed in the combination salEPv-Gal4/UAS-salm-RNAi; UAS-salr-RNAi (Fig. 4C; F; I-K).”

© 2025. Published by The Company of Biologists under the terms of the Creative Commons Attribution License (https://creativecommons.org/licenses/by/4.0/).

Second decision letter

MS ID#: dev.204258R1

MS TITLE: A function of Spalt proteins in heterochromatin organization and maintenance of genomic DNA integrit

AUTHORS: Cristina M. Ostalé, Natalia Azpiazu, Ana Peropadre, Mercedes Martín, Mireya Ruiz-Losada, Ana López-Varea, Rebecca R. Viales, Charles Girardot, Eileen EM Furlong and Jos F. de Celis

Dear Dr de Celis,

I have now received all the referees' reports on the above manuscript, and have reached a decision. The referees' comments are appended below, or you can access them online: please go to:

As you will see, the referees express considerable interest in your work, but still have some significant criticisms and recommend a substantial revision of your manuscript before we can consider publication. If you are able to revise the manuscript along the lines suggested, which may involve further experiments, I will be happy receive a revised version of the manuscript. Your revised paper will be re-reviewed by one or more of the original referees, and acceptance of your manuscript will depend on your addressing satisfactorily the reviewers' major concerns. Please also note that Development will normally permit only one round of major revision. If it would be helpful, you are welcome to contact us to discuss your revision in greater detail. Please send us a point-by-point response indicating your plans for addressing the referees’ comments, and we will look over this and provide further guidance.

Reviewer 1

Advance summary and potential significance to field

In this manuscript, Ostalé et al. assess the impact of a loss of function of Spalt transcription factors on different aspects of Drosophila development. This includes changes in chromatin accessibility and active chromatin marks (H3K27ac) as well as an upregulation of DNA damage genes in developing wings, activation of normally silenced pericentromeric chromatin in eyes and during embryogenesis, mitotic defects and changes to chromatin marks in early embryonic development, and an aberrant nuclear morphology in salivary glands. There is also a comparison of previously published datasets in an effort to relate the observed developmental defects to the DNA damage machinery. This is the second time I am reviewing this paper and it has improved from the first time, but there are, in my opinion, still analyses missing that would support the authors claims better.

Comments for the author

- The first part of the results ("Loss of spalt function has a profound impact on chromatin accessibility") remains rather vague without giving actual numbers: "considerable chromatin regions that increase or reduce their accessibility", "a large number of sequences with accessibility", "enrichment
for genes normally expressed in the wing disc". Wile these numbers are given in the figure, it would be good to also state them in text.
- Related to my first point, it is unclear how many peaks were found in total in the data set (i.e., what fraction changes accessibility). It would be good to to state this, to support the claim of "considerable" chromatin regions changing accessibility and also to give a better feel for the data set. The same goes for H3K27Ac.
- There are several issues with the corresponding figure. Panel G is missing the labels for distance from TSS. In panel L the numbers in the pie charts are all wrong. Numbers and percentages don't match, and sometimes the larger numbers are in the smaller fractions of the chart.

© 2025. Published by The Company of Biologists under the terms of the Creative Commons Attribution License (https://creativecommons.org/licenses/by/4.0/).

- I still find it puzzling that the authors don't show a general comparison of Spalt binding (from Ostalé et al., 2024) and changes in accessibility and H3K27Ac. The authors mention in the discussion that there is no great overlap, but this should be shown in the results. I believe this is something any reader would be interested in seeing.

- Echoing reviewer 2, the second section of results ("Requirements of salm/salr in the maintenance of heterochromatic regions") could start with a better explanation of the different develeopmental stages used in the heterochromatin maintenance experiments. Maybe a sentence or two briefly explaining the models.
- The paper referring to the deficiency line should be cited in the results section (page 10 first paragraph).
- In Fig.2, the eyes of flies with the deficiency and P element on 3rd look much darker than those of flies with the P element on 2nd, but the measurements show higher absorption in the latter. Is there an easy explanation for this?
- Regarding experiments with embryos from heterozygous Df parents: Can the authors exclude the possibility that they didn't just analyze dying embryos since a quarter of the embryos are homozygous for the deficiency? It might be necessary to quantify numbers of embryos that show the respective phenotype - has this been done? If not, then this should be stated as a caveat.
- I still think there should be statistical testing of the values shown in the bar graphs in Fig.2 (e.g., chi-squared).
- For Fig.2 G&H, the changes in nucleus morphology need at least be briefly mentioned here with the note that they are described in more detail later.

- In the last section of results ("Irradiation and loss of Salm/Salr cause overlapping changes in gene expression in the wing disc"), the authors still write "logFC", which is usually used to indicate log(10)FC. What they mean is log(2)FC. This should be changed.
- The text in this section is again a bit vague. The authors write of a "reasonable overlap between the two datasets", which could be further explained.

- Materials & Methods:
- ATAC-seq is lacking information on how many discs were used per replicate.
- There are still two 30 min 4 C incubation steps - I believe there should be only one?
- The text still says that the ATAC libraries were sequenced to 50,000 reads (the correct numbers are now shown in supplementary figure 1).
- The H3K27ac ChIP-seq experiments don't mention the genotypes used for experiment and control (the same as ATAC-seq, but should still be mentioned).

- Typos etc:
- Page 6 end of paragraph 1: delete "is similar"
- Page 7 beginning of second paragraph: must be "posttranslational" instead of "posttranscriptional"
- Page 7 second paragraph: "on the presence of the H3K27ac mark in imaginal cells" change to "on the presence of the H3K27ac mark in imaginal wing disc cells"
- Page 7/8: "In general, the fraction of genes expressed in the wing disc is similar for genes associated by proximity to sequences showing either increases or decreases in H3K27 acetylation" - This sentence is too complicated and should be rewritten.
- Page 17: "pericentric heterochromatin" needs to be "pericentromeric heterochromatin"
- Page 18: "eucaryotic" must be "eukaryotic"
- Page 20: "stalked" must be "stalled"
- Page 23: "wing type wing discs" must be "wild type wing discs"
- Page 29: "Immunocytochemistry" must be "Immunohistochemistry"

Reviewer 2

Advance summary and potential significance to field

The manuscript by Ostale et al. has been revised. Although many of the requested changes by this reviewer have been addressed, there is still one major concern that still exists.

© 2025. Published by The Company of Biologists under the terms of the Creative Commons Attribution License (https://creativecommons.org/licenses/by/4.0/).

- In my last review I have asked for the overlap of Spalt binding and the regions that change their chromatin accessibility, in other words how many of the regions changed are a direct of Spalt binding. In total, it is only 26 out of the many regions that change their accessibility in the absence of Spalt. Although residual amounts of Spalt major protein might be present (due to the RNAi efficiency) , there is a high likelihood that most of the changes in chromatin accessibility and histone mark changes is due to indirect effects (mis-regulation of direct Spalt target genes that are responsible for these changes). One question to that: which genes are mis-regulated that are directly bound by Spalt, which could also explain the changes in chromatin accessibility etc? How many of the Spalt major
- Supplementary Figure 3 is missing.

The authors have now included the very low overlap between Spalt binding and chromatin accessibility changes and have stated that many of the effects could be indirect. But if that is the case, the whole story is way less interesting because Spalt would then NOT be a major regulator of heterochromatin organization and DNA integrity, as stated in the title. Thus, I am still not convinced that this story as written should be published in Development.

<u>Reviewer 3</u>

Advance summary and potential significance to field

as for the first version

Comments for the author

The reviewed version of the paper has clearly improved in terms of how the data is presented. However, there is still a number of small issues the authors should address:

1. On page 15 the authors state: "The increase in expression levels observed after irradiation depends on the transcriptional regulator Dp53 (van Bergeijk et al., 2012)". I am not sure if this is really shown in the cited paper, which lists at least Gadd45 in table S4 as a gene that becomes induced in p53 mutant discs after irradiation and thus, would be regulated by a p53-independent mechanism. This might also affect the discussion of the p53 dependence on pages 20 and 21.

2. In panels D and E of figure 1 the authors should explain that BLACK, BLUE and GREEN represent different types of heterochromatin, while RED and YELLOW are euchromatin types. What do the grey sectors represent?

3. In Fig.1 G the distances from the TSS are missing. Is there a reason why the authors use the 0.05 FDR results in 1G while in 1B they are using the 0.001 FDR list?

4. In Fig.1 L, the sections of the pie-chart are not correctly represented: in the second pie-chart (sal-DOWN) the nine genes represent the smaller (43%, blue) area while the 12 genes should be represented in the larger (57% red) section. In the third pie chart (sal-UP) the red section (26 genes 60%) should be larger than the blue section (17 genes 40%). In the fourth pie-chart (sal-DOWN) the 12 genes should be located in the smaller (blue, 38%) section and the 20 genes in the larger section.

5. Are the eyes shown in Figure 3 A-D representative for their respective genotype? The eye in panel D (3R insertion in Df5 background) shows the highest pigmentation level, but in the plot in panel E the average pigmentation levels of this genotype are lower than those of the 2R insertion in Df5.

6. In Fig.6 A: The authors should point out that the numbers in the pie-charts are percentages (and just happen to also be the absolute numbers of cells in the left chart).

© 2025. Published by The Company of Biologists under the terms of the Creative Commons Attribution License (https://creativecommons.org/licenses/by/4.0/).

7. The authors should check that the references to the figures are all updated in the main text. For example, on page 15: Fig.5 D-G should be corrected to Fig.6 D-G; on page 8: "… and enrichment in H3K27ac labeling (Fig. 1J)" should be Fig. 1 K or L

8. On page 9, the authors state: "More than 30% of the sequence coverage of H3K9me3 and HP1 ChIP-sequencing align to pericentromeric regions (Fig. 2D-E)". Are these the correct panels for this statement? None of the columns are higher than 30%. The statement seems rather to be reflected in panel B.

9. On page 11: the cell divisions in early embryos are synchronous (not synchronic). The authors have now written this statement twice in the same paragraph including the reference. The first time would be enough.

10. The authors switch between p53, P53 and Dp53 throughout the manuscript, figure legends and figures. This should be more consistent.

11. For the Immunocytochemistry: there is no information about the origin of the following primary antibodies in the material and methods section: p53, H2Av, PH3 and H4K12ac

12. For the ATAC-Seq experiment the authors should state in the methods section how many wing discs they used per sample

13. On page 5: "Here we analyzed in different Drosophila tissues that have in common the expression of salm and salr the possible contributions of Sal proteins to chromatin organization." Might be better to say: "We used different salm and salr expressing Drosophila tissues to analyse the possible contributions of Sal proteins to chromatin organization."

14. On page 6: "We detected considerable chromatin regions that increase or reduce their accessibility conformation in salm/salr knock-down conditions compared to wild type discs is similar" The last 2 words of this sentence should be deleted.

15. On page 12: " Fluorescent imaging of DNA from single nucleus showed the existence of a cell population of cells displaying DNA damage that is present in salm/salr knockdown discs but not in control salEPv-Gal4/UAS-GFP discs". It should read "from single nuclei" and "a population of cells" without the "cell" in front of "population".

16. On page 15: "We also searched for changes in expression levels after irradiation of a set of genes that became overexpressed in the central region of salm/salr mutant discs genes". The last word in this sentence should be deleted.

17. On page 23: "Finally, to determine gene expression in wing type wing discs". Shouldn't this be wild type?

Second revision

Author response to reviewers' comments

Reviewer 1

SUGGESTIONS TO AUTHORS

- The first part of the results ("Loss of spalt function has a profound impact on chromatin accessibility") remains rather vague without giving actual numbers: "considerable chromatin regions that increase or reduce their accessibility", "a large number of sequences with accessibility", "enrichment for genes normally expressed in the wing disc". Wile these numbers are given in the figure, it would be good to also state them in text.

© 2025. Published by The Company of Biologists under the terms of the Creative Commons Attribution License (https://creativecommons.org/licenses/by/4.0/).

We added these values in the text:

- page 6, added: "5177, 2994 and 1561 peaks at 0.05, 0.01 and 0.001 FDR, respectively"

- page 6, added and modified: The number of regions that increase or reduce their accessibility conformation in *salm/salr* knock-down conditions compared to wild type discs shows a ratio of 1.14, 1.26 and 1.39 at FDRs 0.05, 0.01 and 0.001, respectively (Fig. 1A; ATAC-UP/ATAC-DOWN values; Supple. Table 1).

- Related to my first point, it is unclear how many peaks were found in total in the data set (i.e., what fraction changes accessibility). It would be good to to state this, to support the claim of "considerable" chromatin regions changing accessibility and also to give a better feel for the data set. The same goes for H3K27Ac.

All the peaks were identified in the comparison between mutant *salm/salr* discs and control discs. In this sense, the number of peaks is shown in Fig. 1A (ATAC peaks) and Fig. 1F (H3K27ac peaks).

- There are several issues with the corresponding figure. Panel G is missing the labels for distance from TSS. In panel L the numbers in the pie charts are all wrong. Numbers and percentages don't match, and sometimes the larger numbers are in the smaller fractions of the chart.

We added the labels to Panel G. We checked the percentages in all panels and corrected Panel L, where we mistakenly placed the numbers in the wrong sector in two panels.

- I still find it puzzling that the authors don't show a general comparison of Spalt binding (from Ostalé et al., 2024) and changes in accessibility and H3K27Ac. The authors mention in the discussion that there is no great overlap, but this should be shown in the results. I believe this is something any reader would be interested in seeing.

We agree with the reviewer, and added a summary of the correlation between Salm binding and the ATAC and H3K27ac datasets:

Page 9, added: "Besides heterochromatin binding, we could only associate 113 genes with Salm binding to either promoter or distal regions (Ostalé et al., 2024; Supple. Table 1). Only 20% of these genes were also associated to increased (*Tim23*, *CG17715*, *CG41520*, *UQCR-11*, *CG40228*, *Myo81F*, *Pzl*, *CR40454*, *CR43242*, *CR45864*, *DIP-lambda*, *AGO3*, *scro*, *l(3)80Fg*, *Cadps*, *CG17684*, *Hsp708a*, *side*, *bru3*, *flam*, *CG41562*, *CG14621*) or reduced (*tyn*, *Eip74EF*, *kirre* and *gpp*) chromatin accessibility (Supple. Table 1). The association between Salm binding and H3K27ac was even lower, and only 11% of genes associated to Salm binding display increased or reduced H3K27ac enrichment (Supple. Table 1)."

- Echoing reviewer 2, the second section of results ("Requirements of salm/salr in the maintenance of heterochromatic regions") could start with a better explanation of the different develeopmental stages used in the heterochromatin maintenance experiments. Maybe a sentence or two briefly explaining the models.

We added the sentences:

Page 10, added: "This effect has been studied mostly using the suppression of the white eye phenotype by transposable elements carrying white+ gene insertions (Schotta et al., 2003)."

Page 10-11, added: "*Drosophila* telomers are maintained by retrotransposons whose transcription is regulated by small RNAs and heterochromatin from adjacent Telomeric Associated Sequences (TAS). Consequently, changes in heterochromatin organization in TAS are correlated with alterations in the expression of HeT-A and TART elements (Capkova Frydrychova et al., 2008)."

- The paper referring to the deficiency line should be cited in the results section (page 10 first paragraph).

© 2025. Published by The Company of Biologists under the terms of the Creative Commons Attribution License (https://creativecommons.org/licenses/by/4.0/).

Page 10, added: (*Df(2L)32FP-5*; Barrio et al., 1999)

- In Fig.2, the eyes of flies with the deficiency and P element on 3rd look much darker than those of flies with the P element on 2nd, but the measurements show higher absorption in the latter. Is there an easy explanation for this?

The reviewer is correct, and this discrepancy correspond to my mistake with the labeling of the graphics, where 3R and 2R were inverted in Fig. 3E. We apologize for this mistake that has now been corrected.

- Regarding experiments with embryos from heterozygous Df parents: Can the authors exclude the possibility that they didn't just analyze dying embryos since a quarter of the embryos are homozygous for the deficiency? It might be necessary to quantify numbers of embryos that show the respective phenotype - has this been done? If not, then this should be stated as a caveat.

All the experiments with embryos derived from *Df(2L)32FP*-5 heterozygous parents were carried out in very early embryos (0-2 hours for experiments involving PCRs; Fig. 3F and mitotic cycle 12-13 for immunological staining; Fig. 3I-N). As the *spalt* deficiency is only lethal at the end of embryogenesis, the early embryonic phenotypes can be assumed as corresponding to homozygous embryos that at this stage are viable. We now discuss this point in material and methods.

Page 22, changed: “We used a chromosomal deficiency for the *salm* and *salr* genes (*Df(2L)32FP5*) (Barrio et al., 1999). This deficiency is a late embryonic and early L1 larval lethal in homozygosity (Ferreiro et al., 2012).”

- I still think there should be statistical testing of the values shown in the bar graphs in Fig.2 (e.g., chi-squared).

We made the chi-squared calculation, which are included in Supplementary Table 2) and referred in the results (p values in pages 9 and 10) and in the material and methods section (page 34).

- For Fig.2 G&H, the changes in nucleus morphology need at least be briefly mentioned here with the note that they are described in more detail later.

I understand the reviewer refers to Fig. 5.

Page 14, changed: “The *spalt* genes are expressed in the nuclei of all salivary gland cells (Fig. 5A), and loss of *salm/salr* function in these cells causes a variety of phenotypes, ranging from rudimentary salivary glands to morphologically normal but smaller glands showing alterations in nucleolar size and nuclear envelope morphology, depending on the particular Gal4/UAS-RNAi combination (Fig. 5).

- In the last section of results ("Irradiation and loss of Salm/Salr cause overlapping changes in gene expression in the wing disc"), the authors still write "logFC", which is usually used to indicate log(10)FC. What they mean is log(2)FC. This should be changed.

We now use log(2)FC through the manuscript.

- The text in this section is again a bit vague. The authors write of a "reasonable overlap between the two datasets", which could be further explained.

Page 15, changed: “We noticed a particular overlap for genes which expression increases in *salm/salr* knockdown wing discs and after irradiation. Thus 18% of genes with increased expression in *salm/salr* knockdown discs (n=78/435) were also identified as displaying increased expression after irradiation (Fig. 6A and Supple Table 2).

- Materials & Methods:

- ATAC-seq is lacking information on how many discs were used per replicate.

© 2025. Published by The Company of Biologists under the terms of the Creative Commons Attribution License (https://creativecommons.org/licenses/by/4.0/).

Page 25, added: “For ATAC-Seq (“Assay for Transposase-Accessible Chromatin using sequencing”) experiments, two replicates of 20 imaginal wing discs from late LIII larvae were used per sample..”

- There are still two 30 min 4 C incubation steps - I believe there should be only one?

Page 25, deleted: “after a 30 m incubation at 4 C”

- The text still says that the ATAC libraries were sequenced to 50,000 reads (the correct numbers are now shown in supplementary figure 1).

Corrected to 50M-90M in page 26

- The H3K27ac ChIP-seq experiments don't mention the genotypes used for experiment and control (the same as ATAC-seq, but should still be mentioned).

Page 26, added: “The larval genotypes were *sal*^{EPV}*-Gal4 UAS-GFP/UAS-salmi-; UAS-salr-i/+* and *sal*^{EPV}*-Gal4 UAS-GFP/+*.”

- Typos etc:

- Page 6 end of paragraph 1: delete "is similar"

Done

- Page 7 beginning of second paragraph: must be "posttranslational" instead of "posttranscriptional"
Done in page 7

- Page 7 second paragraph: "on the presence of the H3K27ac mark in imaginal cells" change to "on the presence of the H3K27ac mark in imaginal wing disc cells"

Done
- Page 7/8: "In general, the fraction of genes expressed in the wing disc is similar for genes associated by proximity to sequences showing either increases or decreases in H3K27 acetylation" - This sentence is too complicated and should be rewritten.

Page 8, changed to: “In general, genes associated by proximity to sequences showing either increases or decreases in H3K27 acetylation are expressed in the wing disc (Fig. 1H).”

- Page 17: "pericentric heterochromatin" needs to be "pericentromeric heterochromatin"

Changed

- Page 18: "eucaryotic" must be "eukaryotic"

Changed

- Page 20: "stalked" must be "stalled"

Changed

- Page 23: "wing type wing discs" must be "wild type wing discs"

Changed

- Page 29: "Immunocytochemistry" must be "Immunohistochemistry"

Changed

Reviewer 2

© 2025. Published by The Company of Biologists under the terms of the Creative Commons Attribution License (https://creativecommons.org/licenses/by/4.0/).

The manuscript by Ostale et al. has been revised. Although many of the requested changes by this reviewer have been addressed, there is still one major concern that still exists.

- In my last review I have asked for the overlap of Spalt binding and the regions that change their chromatin accessibility, in other words how many of the regions changed are a direct of Spalt binding. In total, it is only 26 out of the many regions that change their accessibility in the absence of Spalt. Although residual amounts of Spalt major protein might be present (due to the RNAi efficiency), there is a high likelihood that most of the changes in chromatin accessibility and histone mark changes is due to indirect effects (mis-regulation of direct Spalt target genes that are responsible for these changes). One question to that: which genes are mis-regulated that are directly bound by Spalt, which could also explain the changes in chromatin accessibility etc? How many of the Spalt major

-The authors have now included the very low overlap between Spalt binding and chromatin accessibility changes and have stated that many of the effects could be indirect. But if that is the case, the whole story is way less interesting because Spalt would then NOT be a major regulator of heterochromatin organization and DNA integrity, as stated in the title. Thus, I am still not convinced that this story as written should be published in Development.

We agree with the reviewer that at this stage it is not possible for us without a much further elaborated experimental approach to provide a univocal linear chain of events that applies to every gene that is miss-expressed in *Salm/Salr* mutant conditions, or to every change in chromatin accessibility or Histone mark changing in *Salm/Salr* mutant conditions. Indirect effects on chromatin accessibility and histone mark changes triggered by miss-expression of direct or indirect Salm/Salr target genes can always be an explanation for a fraction of the alterations that we describe. We acknowledge this in page 9 ("*However, a large fraction of changes in ATACseq and H3K27Ac binding are not correlated with the changes in gene expression observed in salm/salr knockdown wing imaginal discs, suggesting that these changes could be caused by indirect mechanisms triggered by salm/salr loss.*") and in page 20 ("*The poor correlation observed between global gene expression with changes in chromatin accessibility in salm/salr knockdown conditions suggest that loss of Sal function indirectly triggers multitude of gene expression alterations that are not caused by direct regulation of target genes by Salm/Salr binding.*"). Furthermore, we are explicitly proposing that a fraction of Salm/Salr effects are indirectly caused by DNA damage and changes in P53 expression/activity. In fact, indirect effects caused by Salm/Salr loss as a consequence of alterations in heterochromatin maintenance and DNA damage response are an integral part to the model we are proposing, and we believe this is clearly stated through the manuscript and in our proposed mechanism discussed in Fig. 7. For these reasons we cannot agree with the reviewer in one of his/her critical points: the low overlap between Salm binding and chromatin accessibility changes implies that Spalt is NOT a major regulator of heterochromatin organization and DNA integrity. Our reasoning is that the roles of Sal proteins as regulators of heterochromatin organization and DNA integrity are stablished through experiments that directly (Figs 3 and 4) or indirectly (Figs 5 and 6) address these aspects of Spalt function. Consequently, we also disagree to the statement that "the whole story is way less interesting" because of the very low overlap between Salm binding and chromatin accessibility changes.

We understand that the main difficulties are to connect the functions of Spalt proteins in heterochromatin organization and DNA integrity with other descriptions of Spalt effects in chromatin accessibility, histone marks and gene expression observed in *sal* hippomorphic conditions, and to connect changes in chromatin accessibility, histone marks and gene expression with Salm binding to the DNA. We believe that to make these connections we should involve mechanisms besides those involving "direct" effects of Salm, specifically if we consider as direct only those changes resulting through a mechanism involving direct binding of Salm to a promoter or regulatory DNA present in a target gene. Our proposal (Fig. 7) is that a novel effect of Sal function involves heterochromatin formation, and participates in long-range effects on chromosomal topology and gene expression.

We apologize for this lengthy explanation, but we thought it was important to somehow share and reduce the concern of the reviewer.

We have tried to clarify our reasoning by introducing small changes to the manuscript:

© 2025. Published by The Company of Biologists under the terms of the Creative Commons Attribution License (https://creativecommons.org/licenses/by/4.0/).

Page 10, added: “The pattern of Salm binding to pericentromeric DNA as well as the alterations in chromatin conformation affecting pericentromeric chromatin, suggest a role for Sal proteins in the generation and/or maintenance of heterochromatic regions that might lead to global effects in gene expression.”

- Supplementary Figure 3 is missing.

Supplementary Figure 3 was only mentioned by mistake in our first response to the reviewer. It should have been Supplementary Figure 2.

Reviewer 3

The reviewed version of the paper has clearly improved in terms of how the data is presented. However, there is still a number of small issues the authors should address:

1. On page 15 the authors state: "The increase in expression levels observed after irradiation depends on the transcriptional regulator Dp53 (van Bergeijk et al., 2012)". I am not sure if this is really shown in the cited paper, which lists at least Gadd45 in table S4 as a gene that becomes induced in p53 mutant discs after irradiation and thus, would be regulated by a p53-independent mechanism. This might also affect the discussion of the p53 dependence on pages 20 and 21.

The values we have from van Bergeijk et al., 2012 in the case of *Gadd45* are:

Gadd45 (2 hours after irradiation):	LOG(2)FC: 4.59 ($p<0.005$)
Gadd45 (2 hours after irradiation in p53 mutant):	LOG(2)FC: 1.266 ($p<0.005$)
Gadd45 (18 hours after irradiation):	LOG(2)FC: 3.026 ($p<0.005$)
Gadd45 (2 hours after irradiation in p53 mutant):	LOG(2)FC: 1.579 ($p<0.005$)

These data suggest that *Gadd45* induction by irradiation depends, at least partially, on p53 activity both early (2 h) and late (18 h). In order to clarify this issue, we have modified Supple. Table 2 to add a new sheet (RX P53 (van Bergeijk, 2012)) including the LOG(2)FC values after irradiation of wild type and p53 mutant wing imaginal discs. Using these data, we can confirm that for genes over-expressed in *salm/salr* knockdown discs and after irradiation, most of this last over-expression depends on p53 function (97% of genes). This general dependance on p53 after irradiation also occurs for genes identified in the classes Sal UP / X-ray down (100%), Sal DOWN / X-Ray UP (91%) and SAL DOWN / X-Ray Down (93%). For this reason, we have not modified the statements made in pages 20 and 21.

2. In panels D and E of figure 1 the authors should explain that BLACK, BLUE and GREEN represent different types of heterochromatin, while RED and YELLOW are euchromatin types. What do the grey sectors represent?

Page 46, added: “The Black, Blue and Green states represent different types of heterochromatin, and the Red and Yellow states correspond to euchromatin. The grey sector represents sequences not associated to any of these stages.”. We also added this information to the main text (page 7).

3. In Fig.1 G the distances from the TSS are missing. Is there a reason why the authors use the 0.05 FDR results in 1G while in 1B they are using the 0.001 FDR list?

We added the distances to TSS in Fig. 1G. The only reason is to include in the comparisons between datasets a similar number of genes. Because at 0.001 FDR we only identified 396 genes in the H3K27ac dataset, we decided to use the 0.05 FDR set, which includes 1031 genes.

4. In Fig.1 L, the sections of the pie-chart are not correctly represented: in the second pie-chart (sal-DOWN) the nine genes represent the smaller (43%, blue) area while the 12 genes should be represented in the larger (57% red) section. In the third pie chart (sal-UP) the red section (26 genes 60%) should be larger than the blue section (17 genes 40%). In the fourth pie-chart (sal-DOWN) the 12 genes should be located in the smaller (blue, 38%) section and the 20 genes in the larger section.

In Panel L we mistakenly placed the numbers in the wrong sectors. We have now corrected panel L.

© 2025. Published by The Company of Biologists under the terms of the Creative Commons Attribution License (https://creativecommons.org/licenses/by/4.0/).

5. Are the eyes shown in Figure 3 A-D representative for their respective genotype? The eye in panel D (3R insertion in Df5 background) shows the highest pigmentation level, but in the plot in panel E the average pigmentation levels of this genotype are lower than those of the 2R insertion in Df5.

The reviewer is correct, and this discrepancy correspond to my mistake with the labeling of the graphics, where 3R and 2R were inverted in Fig. 3E. We apologize for this mistake that has now been corrected.

6. In Fig.6 A: The authors should point out that the numbers in the pie-charts are percentages (and just happen to also be the absolute numbers of cells in the left chart).

We now include this information in Fig. 6A and in the figure legend ("The percentage of overlapping genes is shown in the smaller bottom diagrams (Up/Down, Up/Up; left, and Down/Down and Down/Up; right.").

7. The authors should check that the references to the figures are all updated in the main text. For example, on page 15: Fig.5 D-G should be corrected to Fig.6 D-G; on page 8: "... and enrichment in H3K27ac labeling (Fig. 1J)" should be Fig. 1 K or L.

Checked and corrected.

8. On page 9, the authors state: "More than 30% of the sequence coverage of H3K9me3 and HP1 ChIP-sequencing align to pericentromeric regions (Fig. 2D-E)¬". Are these the correct panels for this statement? None of the columns are higher than 30%. The statement seems rather to be reflected in panel B.

Corrected and changed.

9. On page 11: the cell divisions in early embryos are synchronous (not synchronic). The authors have now written this statement twice in the same paragraph including the reference. The first time would be enough.

Done

10. The authors switch between p53, P53 and Dp53 throughout the manuscript, figure legends and figures. This should be more consistent.

Changed to p53 (Flybase nomenclature) through the manuscript.

11. For the Immunocytochemistry: there is no information about the origin of the following primary antibodies in the material and methods section: p53, H2Av, PH3 and H4K12ac

<u>Changed in page 30</u>: "mouse anti-Fibrilarin (Sigma), anti-HP1 and anti-H2Av (Hybridoma bank at Iowa University), anti-p53 (Santa Cruz Biotechnology; sc-25767), anti-pH3 (Merck: 06-570), anti-H4K12ac (Active Motif: 61527)".

12. For the ATAC-Seq experiment the authors should state in the methods section how many wing discs they used per sample.

<u>Page 25, added</u>: "For ATAC-Seq ("Assay for Transposase-Accessible Chromatin using sequencing") experiments, two replicates of 20 imaginal wing discs from late LIII larvae were used per sample.."

13. On page 5: "Here we analyzed in different Drosophila tissues that have in common the expression of salm and salr the possible contributions of Sal proteins to chromatin organization." Might be better to say: "We used different salm and salr expressing Drosophila tissues to analyse the possible contributions of Sal proteins to chromatin organization."

<u>Page 5; changed to</u>: "Here we analyzed different *Drosophila* tissues expressing the *salm* and *salr* genes to investigate the possible contributions of Sal proteins to chromatin organization."

© 2025. Published by The Company of Biologists under the terms of the Creative Commons Attribution License (https://creativecommons.org/licenses/by/4.0/).

14. On page 6: "We detected considerable chromatin regions that increase or reduce their accessibility conformation in salm/salr knock-down conditions compared to wild type discs is similar" The last 2 words of this sentence should be deleted.

Done

15. On page 12: " Fluorescent imaging of DNA from single nucleus showed the existence of a cell population of cells displaying DNA damage that is present in salm/salr knockdown discs but not in control salEPv-Gal4/UAS-GFP discs". It should read "from single nuclei" and "a population of cells" without the "cell" in front of "population".

Done

16. On page 15: "We also searched for changes in expression levels after irradiation of a set of genes that became overexpressed in the central region of salm/salr mutant discs genes". The last word in this sentence should be deleted.

Done

17. On page 23: "Finally, to determine gene expression in wing type wing discs". Shouldn't this be wild type?

Corrected

Third decision letter

MS ID#: dev.204258R2

MS TITLE: A function of Spalt proteins in heterochromatin organization and maintenance of genomic DNA integrit

AUTHORS: Cristina M. Ostalé, Natalia Azpiazu, Ana Peropadre, Mercedes Martín, Mireya Ruiz-Losada, Ana López-Varea, Rebecca R. Viales, Charles Girardot, Eileen EM Furlong and Jos F. de Celis

Dear Dr de Celis,

I have now received all the referees reports on the above manuscript, and have reached a decision. The referees' comments are appended below, or you can access them online: please go to .

The overall evaluation is positive and we would like to publish a revised manuscript in Development, provided that the referees' comments can be satisfactorily addressed. Please editorially attend to the reviewers' comments in your revised manuscript and detail them in your point-by-point response. If you do not agree with any of their criticisms or suggestions explain clearly why this is so. If it would be helpful, you are welcome to contact us to discuss your revision in greater detail. Please send us a point-by-point response indicating your plans for addressing the referees’ comments, and we will look over this and provide further guidance.

Reviewer 1

Advance summary and potential significance to field

Ostalé et al. show the consequences of loss of function of Sal transcription factors in different Drosophila tissues, which include changes in chromatin accessibility, Histone marks, and nuclear morphology, and the induction of DNA damage (or failure to repair it). Whether the observed phenotypes are a direct effect of the loss of Sal protein function or result from the mis-regulation

© 2025. Published by The Company of Biologists under the terms of the Creative Commons Attribution License (https://creativecommons.org/licenses/by/4.0/).

of one or several Sal target genes is currently unclear, but the authors now mention this caveat in a sufficient way.

Comments for the author

no further suggestions

Reviewer 2

Advance summary and potential significance to field

I have one last comment: the data show that in the wing disc the major function of Spalt is not as a transcriptional regulator but more in chromatin organization, which might be a general feature. This could be stated a bit more clearly, also in the abstract.

Besides that I have no further comments.

Reviewer 3

Advance summary and potential significance to field

In their manuscript, the authors propose a new function for the Drosophila Spalt proteins Salm and Salr. Besides their role as transcription factors acting directly on specific target genes, Salm/Salr also bind to heterochromatic regions of the genome. The authors show, that loss of salm/salr function leads to global changes in chromatin accessibility including heterochromatic regions, and to H3K27 acetylation. They describe changes in chromatin organisation, cell divisions, DNA stability, nuclear envelope shape, and nucleolar size in different cell types upon salm/salr depletion and propose that these changes are a result of Spalt proteins functioning on heterochromatin stability rather than transcriptional repression of specific target genes. However, not all cells express Salm/Salr. Thus, while Spalt proteins are essential for chromatin organisation in some cells, other cells do not require Spalt for this function. This paper provides a starting point for further research on the role of Spalt proteins in Drosophila as well as in vertebrates.

Comments for the author

The desired changes were made satisfactorily.
If possible, the following two small issues should still be addressed:

Page 15: Thus 18% of genes with increased expression in salm/salr knockdown discs were also identified as displaying increased expression after irradiation
Shouldn't this be 15% (78 of 535)?

Supplementary Table 1 legend: ATAC: Genes identified in the ATAC experiments as underrepresented (DOWN) or unrepresented (UP).
Why does UP stand for unrepresented genes?

Third revision

Author response to reviewers' comments

Reviewer 1: SUMMARY OF THE ADVANCE MADE IN THIS PAPER AND ITS POTENTIAL SIGNIFICANCE TO THE FIELD

Ostalé et al. show the consequences of loss of function of Sal transcription factors in different

© 2025. Published by The Company of Biologists under the terms of the Creative Commons Attribution License (https://creativecommons.org/licenses/by/4.0/).

Drosophila tissues, which include changes in chromatin accessibility, Histone marks, and nuclear morphology, and the induction of DNA damage (or failure to repair it). Whether the observed phenotypes are a direct effect of the loss of Sal protein function or result from the mis-regulation of one or several Sal target genes is currently unclear, but the authors now mention this caveat in a sufficient way.

SUGGESTIONS TO AUTHORS

no further suggestions

Reviewer 2:

I have one last comment: the data show that in the wing disc the major function of Spalt is not as a transcriptional regulator but more in chromatin organization, which might be a general feature. This could be stated a bit more clearly, also in the abstract. Besides that I have no further comments.

We added the following modification to the abstract:

"We suggest that these effects are related to roles of Spalt proteins in the regulation of heterochromatin formation and chromatin organization."

Reviewer 3: SUMMARY OF THE ADVANCE MADE IN THIS PAPER AND ITS POTENTIAL SIGNIFICANCE TO THE FIELD

In their manuscript, the authors propose a new function for the Drosophila Spalt proteins Salm and Salr. Besides their role as transcription factors acting directly on specific target genes, Salm/Salr also bind to heterochromatic regions of the genome. The authors show, that loss of salm/salr function leads to global changes in chromatin accessibility including heterochromatic regions, and to H3K27 acetylation. They describe changes in chromatin organisation, cell divisions, DNA stability, nuclear envelope shape, and nucleolar size in different cell types upon salm/salr depletion and propose that these changes are a result of Spalt proteins functioning on heterochromatin stability rather than transcriptional repression of specific target genes.
However, not all cells express Salm/Salr. Thus, while Spalt proteins are essential for chromatin organisation in some cells, other cells do not require Spalt for this function. This paper provides a starting point for further research on the role of Spalt proteins in Drosophila as well as in vertebrates.

SUGGESTIONS TO AUTHORS

The desired changes were made satisfactorily. If possible, the following two small issues should still be addressed:

Page 15: Thus 18% of genes with increased expression in salm/salr knockdown discs were also identified as displaying increased expression after irradiation. Shouldn't this be 15% (78 of 535)?

The number is (Fig. 6A) 79 out of 435, this gives a value of 17,93%, which we squared to 18%.
Supplementary Table 1 legend: ATAC: Genes identified in the ATAC experiments as underrepresented (DOWN) or unrepresented (UP). Why does UP stand for unrepresented genes?

We could have used UNDER and OVER for underrepresented and overrepresented, respectively, but we decided to used DOWN and UP to express the same concepts. The only reason is that in the former expression experiments we used UP and DOWN for genes upregulated or downregulated, respectively, and we wanted to maintain the same abbreviations. We have modified the legend to Supple. Table 1 for clarity.

© 2025. Published by The Company of Biologists under the terms of the Creative Commons Attribution License (https://creativecommons.org/licenses/by/4.0/).

Fourth decision letter

MS ID#: dev.204258R3

MS TITLE: A function of Spalt proteins in heterochromatin organization and maintenance of genomic DNA integrit

AUTHORS: Cristina M. Ostalé, Natalia Azpiazu, Ana Peropadre, Mercedes Martín, Mireya Ruiz-Losada, Ana López-Varea, Rebecca R. Viales, Charles Girardot, Eileen EM Furlong and Jos F. de Celis

Dear Dr de Celis,

I am happy to tell you that your manuscript has been accepted for publication in Development, pending our standard publication integrity checks.

© 2025. Published by The Company of Biologists under the terms of the Creative Commons Attribution License (https://creativecommons.org/licenses/by/4.0/).
